# A safe, effective and adaptable live-attenuated SARS-CoV-2 vaccine to reduce disease and transmission using one-to-stop genome modifications

**DOI:** 10.1038/s41564-024-01755-1

**Published:** 2024-07-12

**Authors:** Jacob Schön, G. Tuba Barut, Bettina Salome Trüeb, Nico Joel Halwe, Inês Berenguer Veiga, Annika Kratzel, Lorenz Ulrich, Jenna N. Kelly, Melanie Brügger, Claudia Wylezich, Adriano Taddeo, Etori Aguiar Moreira, Demeter Túrós, Llorenç Grau-Roma, Ann Kathrin Ahrens, Kore Schlottau, Tobias Britzke, Angele Breithaupt, Björn Corleis, Jana Kochmann, Blandina I. Oliveira Esteves, Lea Almeida, Lisa Thomann, Christelle Devisme, Hanspeter Stalder, Silvio Steiner, Sarah Ochsenbein, Kimberly Schmied, Fabien Labroussaa, Jörg Jores, Philip V’kovski, Vladimir Cmiljanovic, Marco P. Alves, Charaf Benarafa, Nadine Ebert, Donata Hoffmann, Martin Beer, Volker Thiel

**Affiliations:** 1 Institute of Diagnostic Virology, Friedrich-Loeffler-Institut, Greifswald-Insel Riems, Germany; 2 Institute of Virology and Immunology, Bern and Mittelhäusern, Bern, Switzerland; 3 Department of Infectious Diseases and Pathobiology, Vetsuisse Faculty, University of Bern, Bern, Switzerland; 4 Multidisciplinary Center for Infectious Diseases, University of Bern, Bern, Switzerland; 5 European Virus Bioinformatics Center, Jena, Germany; 6 Department of Experimental Animal Facilities and Biorisk Management, Friedrich-Loeffler-Institut, Greifswald-Insel Riems, Germany; 7 Graduate School for Cellular and Biomedical Sciences, University of Bern, Bern, Switzerland; 8 Institute of Immunology, Friedrich-Loeffler-Institut, Greifswald-Insel Riems, Germany; 9 Institute of Veterinary Bacteriology, Department of Infectious Diseases and Pathobiology, Vetsuisse Faculty, University of Bern, Bern, Switzerland; 10 RocketVax AG, Basel, Switzerland

**Keywords:** Live attenuated vaccines, Viral infection

## Abstract

Approved vaccines are effective against severe COVID-19, but broader immunity is needed against new variants and transmission. Therefore, we developed genome-modified live-attenuated vaccines (LAV) by recoding the SARS-CoV-2 genome, including ‘one-to-stop’ (OTS) codons, disabling Nsp1 translational repression and removing ORF6, 7ab and 8 to boost host immune responses, as well as the spike polybasic cleavage site to optimize the safety profile. The resulting OTS-modified SARS-CoV-2 LAVs, designated as OTS-206 and OTS-228, are genetically stable and can be intranasally administered, while being adjustable and sustainable regarding the level of attenuation. OTS-228 exhibits an optimal safety profile in preclinical animal models, with no side effects or detectable transmission. A single-dose vaccination induces a sterilizing immunity in vivo against homologous WT SARS-CoV-2 challenge infection and a broad protection against Omicron BA.2, BA.5 and XBB.1.5, with reduced transmission. Finally, this promising LAV approach could be applicable to other emerging viruses.

## Abstract (web summary)

A live-attenuated SARS-CoV-2 vaccine based on one-to-stop codon modifications can elicit broad, systemic and mucosal immunity, as well as reduced transmission of SARS-CoV-2 in vivo.

## Main

The emergence of severe acute respiratory syndrome coronavirus 2 (SARS-CoV-2) in 2019 spurred global spread and variant evolution^1^. Despite rapid messenger (m)RNA and viral vector vaccine development, current approved vaccines, administered intramuscularly, target the spike protein antigen, offering limited protection against new variants and transmission. Consequently, SARS-CoV-2 can evade immunity through spike gene mutations, hindering consistent infection control^1^. Hence, more robust vaccination strategies are urgently needed for broader immunogenicity. We introduced an alternative approach for developing SARS-CoV-2 live attenuated vaccines (LAVs) using the one-to-stop (OTS) method. This technique exploits RNA virus polymerase’s natural error rate to weaken the virus^2^. By making synonymous codon changes in ORF1ab, we maintain the same amino acid sequence as the wild-type (WT) virus but increase the chance of premature termination codons, reducing viral fitness and pathogenicity. We also enhance safety and antigenicity by mutating non-structural protein 1 (Nsp1) and deleting specific open reading frames (ORFs) (6–8) and the polybasic spike S1/S2 cleavage site (PCS). Eliminating Nsp1’s function aids viral attenuation^3^, while deleting ORFs associated with immune evasion mechanisms^4–7^ promotes early interferon responses, enhance LAV attenuation and improve immunogenicity^8–11^. Removal of the PRRAR motif from the PCS further contributes to attenuation and transmission efficiency reduction^12–14^.

We produced multiple vaccine candidates using the OTS method, adjusting their attenuation levels by modifying the genome. Enriching OTS codons made them more susceptible to mutagenic drugs. Combining Nsp1 (K164A/H165A) mutations with ORF6–8 knockout yielded a highly protective LAV ‘OTS-206’ against severe disease caused by different virus variants. Another candidate, OTS-228, with an additional PCS deletion, prevented LAV transmission without compromising its protective efficacy. Through in vitro and animal model testing with K18-hACE2 mice and Syrian hamsters, we showed that our LAV candidates are safe and provide long-lasting immunity. They offer sterilizing immunity against the original SARS-CoV-2 strain and protect against recent variants such as Omicron BA.2, BA.5 and XBB.1.5 after a single intranasal (i.n.) dose. Overall, our innovative LAV candidates based on the OTS approach present a robust and adaptable SARS-CoV-2 vaccination solution. They are easy to administer, induce strong protective responses, prevent severe illness and reduce viral spread and breakthrough infections. With exceptional safety and efficacy, these candidates stand as viable alternatives to current mRNA vaccines.

## Results

### Development of SARS-CoV-2 LAV candidates via the OTS approach

**Fig. 1:**
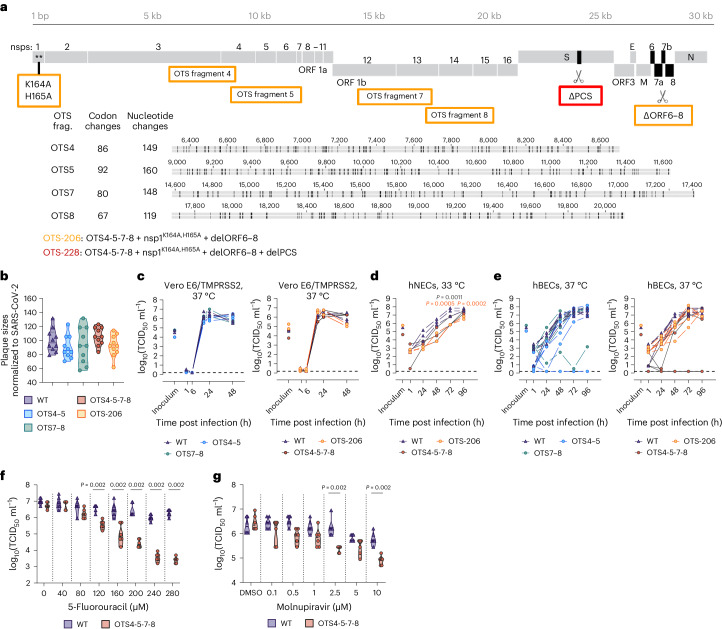
OTS constructs exhibit similar replication kinetics to WT in vitro but are more sensitive to mutagenic drugs. **a**, Overview of mutations introduced into the SARS-CoV-2 genome to generate LAVs. Fragments 4, 5, 7 and 8 were modified to generate one-to-stop codons. Specific changes are indicated for each fragment. OTS-206 also has additional Nsp1 mutations (K164A/H165A) and deletions of ORF6 to ORF8. OTS-228 is additionally missing the PCS. **b**, Violin plot of individual plaque sizes in Vero E6/TMPRSS2 cells. Mean plaque sizes (indicated by a line) were comparable between OTS and WT viruses (*n* = 10 plaques measured per group). No significant difference was found using ordinary one-way ANOVA and *P* values were adjusted using Tukey’s multiple-comparisons test. **c**–**e**, Growth kinetics of WT and OTS viruses in Vero E6/TMPRSS2 cells (**c**) (*n* = 3 independent biological replicates per group), hNECs (**d**) (*n* = 3 independent biological replicates per group) and hBECs (**e**) (*n* = 6; 3 independent biological replicates from 2 donors per group). Samples were collected at designated timepoints and assessed for infectious particle titres using TCID_50_. Graphs show each biological replicate. Statistical analysis was performed using two-way ANOVA. **f**,**g**, Treatment of Vero E6/TMPRSS2 cells with 5-fluorouracil (5-FU) and molnupiravir (*n* = 6 independent biological replicates per group), followed by infection with WT or OTS4-5-7-8, indicating a higher sensitivity of OTS-4-5-7-8 to 5-FU and molnupiravir. Statistical significance was assessed using two-sided, unpaired, non-parametric multiple *t*-test with Mann–Whitney test (compared ranks). *P < 0.05, **P < 0.01, ***P < 0.001, ****P < 0.0001. Additional data in Extended Data Fig. 1. Source data

**Extended Data Fig. 1:**
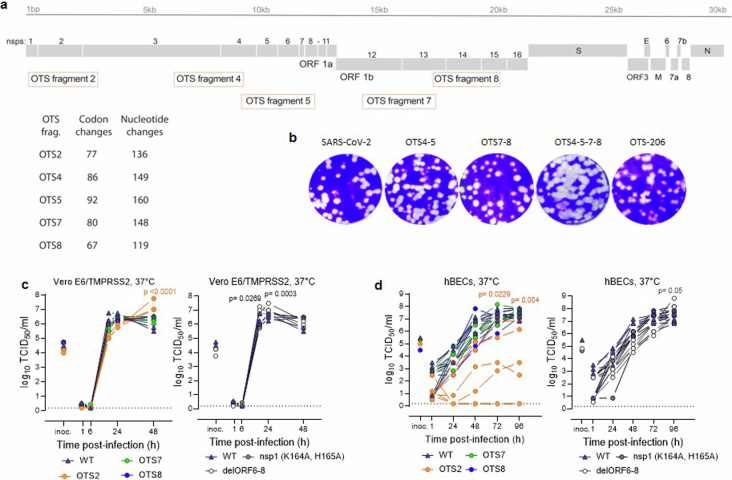
OTS constructs show comparable replication kinetics to WT in vitro, but higher sensitivity to treatment with antivirals. **a**, Schematic overview of the mutations introduced to SARS-CoV-2 genome to generate OTS codons. Fragments 2, 4, 5, 7, and 8, which are used for TAR cloning of recombinant SARS-CoV-2 clones have been modified to enrich the number of one-to-stop codons. The number of codons and nucleotides that have been changed are indicated for each fragment. For the OTS-206 construct, two additional point mutations were introduced in Nsp1 (K164A/H165A) and open reading frames ORF6 to ORF8 were deleted. The number of codons and nucleotides that have been changed in each fragment are listed in Supplementary Table 1. **b**, Representative pictures of the plaque sizes of viruses in 6-well plates 2 dpi. Virus growth kinetics assessed by TCID50 assay on **c**, Vero E6/TMPRSS2 cells (n=3) and **d**, human bronchial epithelial cell (hBECs) (n=6 (3 replicates from 2 donors)). Each line in the graphs shows the titers obtained from one individual sample. Statistical significance was determined using two-sided, two-way ANOVA and p-values were adjusted using Tukey’s multiple comparison test (CI: 95%); *P<0.05, **P<0.01, ***P<0.001, ****P<0.0001. Source data

We utilized yeast transformation-associated recombination (TAR) cloning to integrate OTS changes into the SARS-CoV-2 genome^15^, targeting specific serine and leucine codons in ORF1ab (Fig. 1a). This led to various mutants: OTS2, OTS4, OTS5, OTS7 and OTS8 (Fig. 1a, Extended Data Fig. 1a and Supplementary Table 1). Combinations of these fragments produced mutants such as OTS4–5, OTS7–8 and finally OTS4-5-7-8, with 576 mutations and 325 synonymous codon changes in recoded ORF1ab (Supplementary Table 1).

For subsequent OTS LAV candidates, we used extensively recoded ORF1ab from OTS4-5-7-8 as the base. OTS-206 combined these mutations with two amino acid changes (K164A, H165A) in Nsp1 and the deletion of accessory genes ORF6–8 (Fig. 1a). For the final candidate, OTS-228, we removed the polybasic spike S1/S2 cleavage site (ΔPRRAR) from OTS-206 (Fig. 1a).

### OTS constructs are more sensitive to mutagenic drugs

To assess the impact of the OTS modifications, we compared plaque sizes and replication kinetics of OTS viruses to WT SARS-CoV-2 in Vero E6/TMPRSS2 cells. Compared with WT, OTS4–5, OTS7–8 and OTS-206 had slightly smaller plaques, while OTS4-5-7-8 had larger ones (Fig. 1b and Extended Data Fig. 1b). We further assessed viral replication kinetics in Vero E6/TMPRSS2 cells, human nasal and bronchial epithelial cells (hNECs and hBECs). OTS4–5, OTS7–8, OTS4-5-7-8 and OTS-206 replicated similar to WT in Vero E6/TMPRSS2 cells but showed differences in hNECs and hBECs (Fig. 1c–e and Extended Data Fig. 1c,d). In hNECs, OTS4-5-7-8 and OTS-206 had lower titres up to 96 h post infection (hpi) (Fig. 1d). In hBECs, OTS4–5, OTS7–8 and OTS4-5-7-8 titres varied between donors, while OTS-206 reached similar titres as WT at 96 hpi (Fig. 1e). Controls for OTS-206 included recombinant viruses with Nsp1 mutation (K164A, H165A) or deletion of accessory ORFs 6–8 (delORF6–8) (Extended Data Fig. 1c,d). The Nsp1 mutant showed kinetics similar to WT, while delORF6–8 had increased titres at 24 hpi in Vero E6/TMPRSS2 and 96 hpi in hBECs (Extended Data Fig. 1c,d). Furthermore, we tested OTS4-5-7-8’s susceptibility to 5-fluorouracil (5-FU) and molnupiravir. OTS4-5-7-8 had significantly reduced titres in a dose-dependent manner with 5-FU compared with WT (Fig. 1f). Similarly with molnupiravir, OTS4-5-7-8 replication was lower than WT (Fig. 1g).

### Stability of OTS modifications

OTS4–5, OTS7–8, OTS-228 and WT SARS-CoV-2 were sequenced after 10 or 15 passages in Vero E6 cells. WT was passaged 15 times as a control. OTS4–5, OTS7–8 and WT lost the S1/S2 cleavage site (S 679-NSPRRAR-685), typical in TMPRSS2-deficient environments such as Vero E6 cells. However, OTS-206’s S1/S2 cleavage site and OTS-228’s PRRAR deletion remained unchanged in Vero E6/TMPRSS2 cells (Supplementary Table 5). Crucially, OTS codons did not revert to WT after 10 passages (OTS4–5, OTS7–8 and OTS-206) or 15 passages (OTS-228) in Vero E6 or Vero E6/TMPRSS2 cells. Nsp1 mutations (K164A, H165A) in OTS-206 and OTS-228, and ORF6–8 deletions remained unchanged.

### OTS adjustments influence in vivo attenuation

**Extended Data Fig. 2:**
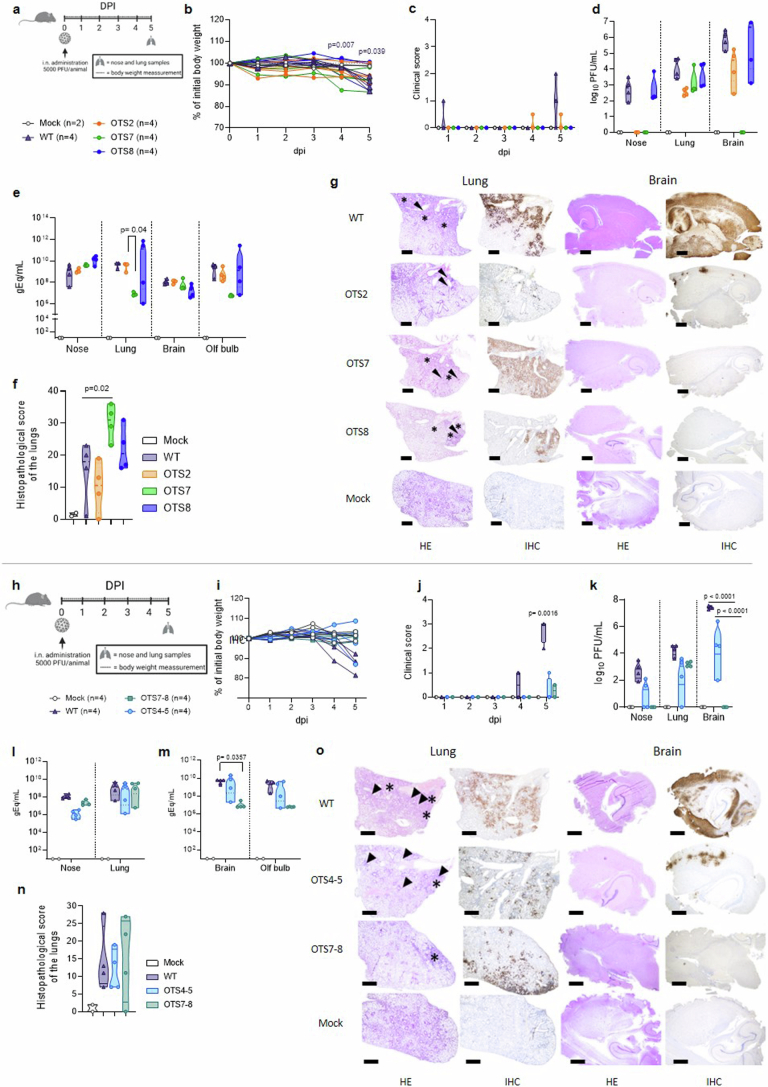
Attenuation of OTS2, OTS7, OTS8, OTS4-5 and OTS7-8 in K18-hACE2 mice. **a**, K18-hACE2 mice (7–16 weeks old, n=4 mice/group, n=2 mice/mock group) were infected with 5,000 PFU of either OTS2, OTS7, OTS8, or SARS-CoV-2 WT virus, or only with medium for 5 days. Overview created with BioRender.com. **b, c**, Mice were monitored for body weight change (mean ± SEM) (Mock vs WT, p=0.0442) and clinical symptoms over the 5-day course of infection. On day 5 post-infection (dpi), mice were euthanized, and samples from the nose, lungs, brain, and olfactory bulbs were collected for evaluation of infectious virus titers, viral genome copy numbers, and pathology. **d**, Infectious virus titers from the nose, lung, and brain samples were determined using plaque assays in VeroE6 cells. **e**, Genome copy numbers in the nose, lung, brain, and olfactory bulb samples of infected mice were quantified using probe-specific RT–qPCR. **f**, Histopathological lung score was given for characterization and comparison of the severity of lung lesions. **g**, Hematoxylin and eosin stain (left panel) and immunohistochemical analysis (right panel) specific for SARS-CoV-2 nucleocapsid protein of lung and brain sections. Scale bar, 500 μm. **h**, Experimental setup of comparison of OTS4-5, OTS7-8 to WT infection in short-term (7–16 weeks-old, n=4 mice/group). Overview created with BioRender.com. **i, j**, Mice were monitored for body weight (mean ± SEM) (Mock vs WT, p<0.0001) change and clinical symptoms over the 5-day course of infection. **k**, Infectious virus titers from the nose, lung, and brain samples were determined using plaque assays. **l, m**, Genome copy numbers in the nose, lung, brain, and olfactory bulb samples of infected mice were quantified using RT–qPCR. **n**, Histopathological lung score was given for characterization and comparison of the severity of lung lesions. **o**, Hematoxylin and eosin stain (left panel) and immunohistochemical analysis (right panel) specific for SARS-CoV-2 nucleocapsid protein of lung and brain sections (n=4 per group) (magnification 50x). Scale bar, 500 μm. Statistical significance was determined using two-sided, two-way ANOVA (b-f, i-m), and P values were adjusted using Tukey’s multiple-comparison test or was assessed by two-sided, unpaired, nonparametric multiple t-test with Mann-Whitney test (compared ranks, CI: 95%); *P < 0.05, **P < 0.01, ***P < 0.001, ****P < 0.0001. Data were obtained from one experiment, with each data point representing one biological replicate. Body weight changes, clinical scores, and histopathological scores of all K18-hACE2 mice experiments are shown in Supplementary Table 6. Source data

**Extended Data Fig. 3:**
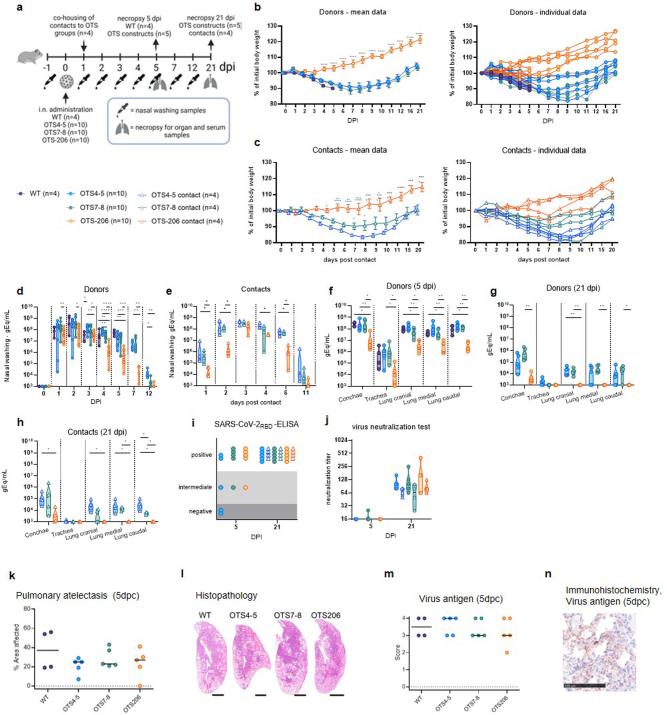
Safety study of OTS4-5, OTS7-8 and OTS-206 in Syrian hamster model. **a**, Experimental setup of intranasal inoculation of Syrian hamsters (*Mesocricetus auratus*, male, 12 weeks old (WT, OTS4-5 and OTS7-8) and 8 weeks old (OTS-206) (Donors) (n=10 hamsters/OTS-group until 5dpi, n=5 hamsters/OTS-group until 21dpi, n=4 hamster WT-group until 5dpi) with OTS4-5, OTS7-8, or OTS-206 SARS-CoV-2, including co-housing of naïve contact animals (Contacts) (n=4 hamsters/group until 21dpi) 1 day post inoculation (dpi). Data obtained from two independent experiments (experiment 1 (OTS4-5, OTS7-8, WT) and experiment 2 (OTS-206). Overview created with BioRender.com. **b, c**, Body weight changes of inoculated and contact hamsters in percent as mean data (+/- SEM) and on individual level. **d, e**, Virus genome copy numbers in nasal washings of donor and contact hamsters. **f, g**, Virus genome copy numbers in the organ samples of donors at 5 and 21 dpi. **h**, Virus genome copy numbers in the organ samples of contact hamsters at 21 dpi. **i**, Serum samples of 5 and 21 dpi analyzed by SARS-CoV-2RBD-ELISA. **j**, Serum samples were additionally analyzed in live virus neutralization assay (capacity to neutralize 100 TCID50) against ancestral WT SARS-CoV-2. **k**, Pneumonia-induced pulmonary atelectasis 5 dpi given in % affected area. **l**, Histopathology, lung whole slide images showing atelectasis, hematoxylin-eosin stain. Scale bar, 2.5 mm. **m**, Virus antigen score, 0= no antigen, 1 = focal, 2 = multifocal, 3 = coalescing, 4 = diffuse. **n**, Representative virus antigen imaging (WT group) by immunohistochemistry (SARS-CoV nucleocapsid protein detection), mainly in type-1 pneumocytes, bar 100 µm. Statistical significance was assessed by two-sided, unpaired, nonparametric multiple t-test with Mann-Whitney test (compared ranks, CI: 95%) (panel **b-h**). *P<0.05, **P < 0.01, ***P<0.001, ****P<0.0001. No asterisk indicates no statistical significance. Source data

Attenuation level of the OTS constructs was tested in K18-hACE2 mice (Extended Data Fig. 2a) and Syrian hamsters (Extended Data Fig. 3a). In K18-hACE2 mice, OTS2, OTS7 and OTS8 caused no significant weight loss (Extended Data Fig. 2b) or symptoms (Extended Data Fig. 2c) for 5 days post inoculation (dpi). However, infectious virus titres (Extended Data Fig. 2d), genome copies (Extended Data Fig. 2e) and lung pathology (Extended Data Fig. 2f,g) matched WT, but no infectious virus was found with OTS2 and OTS7 in the nasal conchae or OTS7 in the brain (Extended Data Fig. 2d). To enhance attenuation, OTS fragments 4–5 and 7–8 were combined (OTS4-5-7-8) (Extended Data Fig. 2h). OTS4-5-7-8 and OTS-206 were tested in vivo. In K18-hACE2 mice, WT-infected mice and one OTS4–5-infected mouse lost weight (Extended Data Fig. 2i). Only WT-infected mice showed signs at 5 dpi (Extended Data Fig. 2j). OTS4–5- and OTS7–8-infected mice had lower virus titres in lungs, noses and brains (Extended Data Fig. 2k), while RNA copies remained high (Extended Data Fig. 2l,m). N protein levels in brains were minimal with OTS constructs (Extended Data Fig. 2g,o). In Syrian hamsters, unlike OTS-206, OTS4–5 and OTS7–8 induced weight loss similar to WT (Extended Data Fig. 3b). OTS-206 had fewer genome copies in nasal washings and respiratory tissues compared with OTS4–5 and OTS7–8 (Extended Data Fig. 3d,f,g). Lung histopathology showed typical SARS-CoV-2 lesions with virus antigen primarily in type I pneumocytes (Extended Data Fig. 3k,l). Transmission occurred from OTS4–5- and OTS7–8-infected hamsters to contacts, causing weight loss, unlike OTS-206 contacts (Extended Data Fig. 3c). Contact animals showed viral RNA in nasal washings (Extended Data Fig. 3d,e) and organs (Extended Data Fig. 3h), and seropositivity (Extended Data Fig. 3i,j), confirming transmission. Sequencing at 21 dpi confirmed stable OTS codons in conchae samples of OTS4–5 and OTS7–8 contact animals (Supplementary Table 5). In summary, OTS4–5 and OTS7–8 are modestly attenuated, reducing virulence but not weight loss or viral shedding. However, OTS-206, with four recoded genome fragments, is significantly attenuated, eliciting no weight loss in K18-hACE2 mice and hamsters, and showing restricted replication and genome stability.

### OTS constructs induce full SARS-CoV-2 protection

**Fig. 2:**
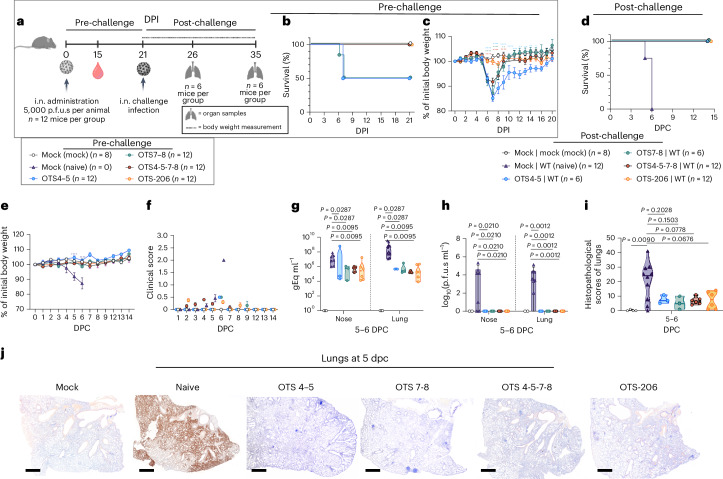
Immunization with OTS constructs provides full protection against SARS-CoV-2 challenge in sensitive preclinical K18-hACE2 mice model. **a**, Intranasal inoculation of age-matched K18-hACE2 mice (*n* = 12 mice per group) with OTS4–5, OTS7–8, OTS4-5-7-8 and OTS-206, and subsequent challenge with WT at 21 dpi (*n* = 12 mice per group, *n* = 6 mice in OTS4–5 and OTS7–8 groups). A mock (vaccinated and challenged with culture media) and a naïve control group (vaccinated with only media, but infected with challenge virus) were included. Data were obtained from one experiment. Overview created with BioRender.com. **b**,**c**, Pre-challenge survival (**b**, %) and body weight (**c**) (*n* = 12 mice (OTS), *n* = 8 (mock), group mean ± s.e.m.) loss showed correlation between increased OTS modifications and improved outcomes. **d**,**e**, All OTS constructs provided full protection against challenge infection in terms of survival (**d**) and body weight (**e**) (*n* = 12 mice in naïve, OTS-4-5-7-8, OTS-206 and OTS4–5 (until 7 dpi) and OTS7–8 (until 6 dpi) groups; *n* = 6 mice in OTS4–5 (from 7 dpi onwards), *n* = 10 mice in OTS7–8 at 6 dpi and *n* = 6 mice from 7 dpi onwards; *n* = 8 mice in the mock group) (group mean ± s.e.m.). **f**, Clinical scores post challenge were high only in naïve mice (group mean ± s.e.m.). **g**, Viral genome copies in nose and lung samples were significantly reduced for the vaccinated animals (*n* = 3 mice in OTS4–5, OTS7–8 groups; *n* = 6 mice in OTS4-5-7-8, OTS-206 groups; *n* = 4 in mock group, *n* = 10 in naïve group). **h**, At 5–6 dpc, no infectious virus was detectable in nose and lung samples of the vaccinated animals. **i**,**j**, Histopathological scores (**i**) and immunohistochemical analysis (**j**) of lung sections demonstrate protection in OTS-construct-inoculated mice. Scale bar, 500 μm. Statistical significance was calculated using two-sided ordinary two-way ANOVA with Tukey´s multiple-comparisons test (compare columns (simple effect within rows), 95% CI) for **c** and **e**; and two-sided, unpaired, non-parametric multiple *t*-test with Mann–Whitney test (compare ranks, 95% CI) for **g**–**i**. Violin plots in **g**–**i** show individual samples with mean values (middle lines). Source data

**Fig. 3:**
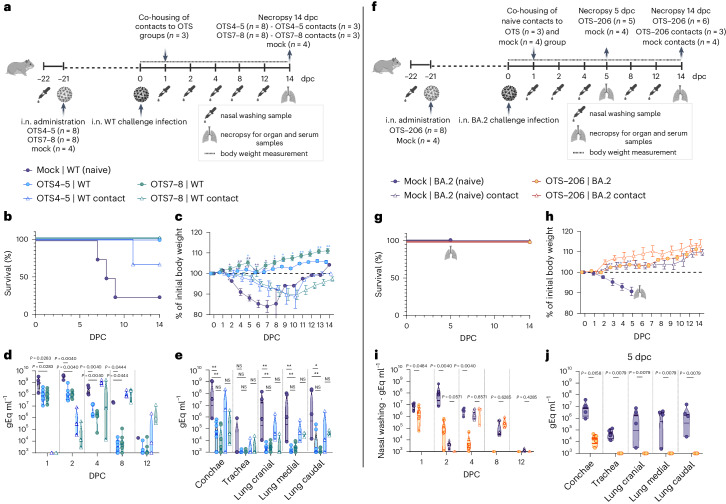
Immunization with OTS constructs provides full protection against SARS-CoV-2 challenge in the Syrian hamster. **a**, Syrian hamsters (*M. auratus*, male, 12 weeks old) inoculated with either no vaccine (naïve/control) or OTS4–5 or OTS7–8 (*n* = 8 hamsters per group, *n* = 4 hamsters for naïve/control group), and subsequently challenged. Overview created with BioRender.com. **b**–**e**, Survival (**b**) and weight stability (**c**) post challenge (mean ± s.e.m.), with reduced viral genome copies in nasal washings (**d**) and respiratory tissues (**e**). Nevertheless, contact animals (*n* = 3 hamsters per group) became infected, leading to body weight loss (**c**), virus shedding in nasal washings (**d**) and virus genome loads in respiratory tract samples (**e**). **f**, Similar results are observed in hamsters (male, 8 weeks old) inoculated with OTS-206 (*n* = 8, *n* = 4 hamsters for naïve/control group) and challenged with BA.2 VOC. Overview created with BioRender.com. **g**,**h**, Challenged hamsters did not exhibit any mortality (**g**) and vaccinated animals were protected from weight loss (**h**) (mean ± s.e.m.). **i**,**j**, Detected amount of virus genome was significantly reduced in the nasal washings (**i**) as well as in conchae samples (**j**) and was absent in all lung samples examined at 5 dpc. More data, such as serology and individual body weights, are presented in Extended Data Fig. 4 and Supplementary Fig. 6. Statistical significance was assessed using two-sided, unpaired, non-parametric multiple *t*-test with Mann–Whitney test (compare ranks, 95% CI) (**c**–**e**, **h**–**j**). *P < 0.05, **P < 0.01, ***P < 0.001, ****P < 0.0001; NS, not significant. Data obtained from two independent experiments (**a**–**e** and **f**–**j**). Violin plots in **d**, **e**, **i** and **j** show individual samples with mean values (middle lines). Source data

**Extended Data Fig. 4:**
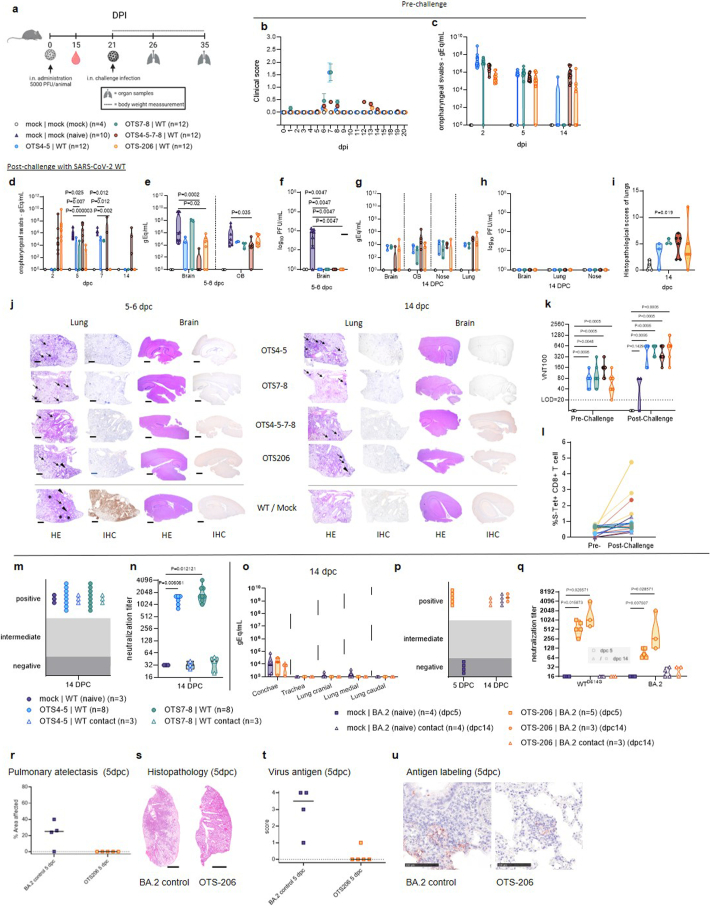
Immunization with OTS4-5, OTS7-8, OTS4-5-7-8, and OTS-206 protects K18-hACE2 mice and Syrian hamsters from SARS-CoV-2 infection. **a**, K18-hACE2 transgenic mice (7-16 weeks of age, n=8 mice/group) were immunized with either OTS viruses and SARS-CoV-2 WT or mock medium. Blood samples were taken on day 15 post-immunization. At 21 days post-immunization, mice were challenged with SARS-CoV-2 WT and euthanized on days 5 and 14 post-challenge to obtain organ samples (nose, lungs, brain, and olfactory bulbs) for evaluation. Overview created with BioRender.com. **b**, Clinical symptoms over the course of infection (mean +/- SEM) and **c**, genome copy numbers (genome equivalence per ml, gEq/mL) oropharyngeal swabs and in post challenge samples ((**d**, orophayngial swabs, **e**, brain and olfactory bulb (OB) 5-6 dpc) and **g**, brain, OB, nose and lung 14 dpc).**f, h**, Infectious virus titers from the brain, lung and nose samples. **i**, Histopathological lung scores at 14 dpc. **j**, Hematoxylin and eosin stain of lung sections (n=4 per group and time point). Pneumonia (asterisk), perivascular and peribronchiolar cuffings (arrowhead), tertiary lymphoid follicle formations (arrow). Scale bar, 500 μm. **k**, Virus neutralization test of 15 dpi (pre-challenge) and 5 and 14 dpc (post-challenge) sera against WT. **l**, Share of CD8^+^ T cells reacting to spike-stimulated condition. Workflow is given in Supplementary Fig. 1 and in the methods section. **m**, Sera samples from OTS4-5 or OTS7-8 vaccinated Syrian hamsters challenged with SARS-CoV-2 WT (Fig. 3a), as well as sera of co-housed contact animals, were analyzed and tested positive by SARS-CoV-2-RBD specific ELISA. **n**, Additionally, virus neutralizing capacity was confirmed for these samples, while mock, OTS4-5 contact, and OTS7-8 contact animals showed only low titers. **o**, Organ samples of 14 dpc of OTS-206 vaccinated and subsequently SARS-CoV-2 Omicron BA.2 challenged Syrian hamsters (Fig. 3f) were analyzed by RT-qPCR. **p**, Serological evaluation by SARS-CoV-2-RBD specific ELISA confirmed transmission of BA.2 challenge virus to the naïve contact animals for the mock vaccinated as well as for the OTS-206 vaccinated animals. **q**, Live virus neutralizing capacity comparison showed substantial neutralizing titers of the OTS-206 vaccinated animals against ancestral SARS-CoV-2 and Omicron BA.2 VOC. **r**, Pneumonia-induced pulmonary atelectasis at 5 dpi was assessed, and **s**, histopathology images showed atelectasis. Scale bar, 2.5 mm. **t**, Virus antigen score and **u**, immunohistochemistry for SARS-CoV nucleocapsid protein detection were conducted. Scale bar, 100 µm. Statistical significance was assessed by two-sided, unpaired, nonparametric multiple t-test with Mann-Whitney test (compared ranks) (panel **d-f, i, k, n** and **q**). *P<0.05, **P<0.01, ***P<0.001, ****P<0.0001. No asterisk indicates no statistical significance. Source data

To assess the protective efficacy of OTS4-5-7-8 and OTS-206 compared to OTS4–5 and OTS7–8, we intranasally immunized K18-hACE2 mice (Fig. 2a and Extended Data Fig. 4a). Pre-challenge, OTS4-5-7-8 and OTS-206 mice showed no significant weight loss or symptoms (Fig. 2b,c and Extended Data Fig. 4b,c), unlike half of OTS4–5 or OTS7–8 mice, which were euthanized (Fig. 2b,c). After 21 days, all mice were challenged with WT SARS-CoV-2. Naïve control mice reached a humane endpoint 5 or 6 days post challenge (Fig. 2d–f), while OTS4–5 and OTS7–8 mice recovered rapidly with no significant weight loss or clinical signs (Fig. 2d–f). OTS-immunized mice had significantly lower viral genome copies in nasal and lung samples compared with non-immunized mice (Fig. 2g,h and Extended Data Fig. 4e–h). No infectious virus was found in samples from pre-immunized and challenged K18-hACE2 mice, indicating virus clearance (Fig. 2h and Extended Data Fig. 4f,h). Histopathological analysis showed mild lung leucocytic infiltrates with follicle formation in OTS4–5-immunized mice, while OTS4-5-7-8-immunized mice had moderate to severe lung pathology (Fig. 2i,j and Extended Data Fig. 4i,j). However, OTS-206-vaccinated mice had minor infection signs that resolved quickly (Fig. 2i,j and Extended Data Fig. 4i,j). OTS LAV-candidates provided protection against lethal SARS-CoV-2 challenge infection, inducing neutralizing antibody responses (Extended Data Fig. 4k) and SARS-CoV-2 spike-specific CD8^+^ T cell responses (Extended Data Fig. 4l). The protective efficacy of OTS-LAVs was tested in Syrian hamsters as well. No deaths or weight loss were observed in hamsters immunized with OTS4–5 or OTS7–8 and challenged with WT SARS-CoV-2, unlike for the naive controls (Fig. 3a–c). Nasal washing samples from immunized groups had significantly lower viral genome copies (Fig. 3d). At 14 days post challenge, viral loads in immunized animals were barely above threshold, indicating virus clearance (Fig. 3e). However, transmission to naive contacts occurred, evidenced by increased mortality, weight loss and viral genome detection (Fig. 3b–e and Extended Data Fig. 4m,n). Next, hamsters received OTS-206 immunization and SARS-CoV-2 Omicron BA.2 challenge (Fig. 3f). No mortality occurred in immunized or naive contact hamsters (Fig. 3g). Immunized hamsters showed no weight loss, unlike challenged naive controls (Fig. 3h). Viral RNA in nasal washing samples significantly decreased in the immunized group compared with controls (Fig. 3i), and virus transmission to contacts was delayed (Fig. 3i). Lungs were protected against Omicron BA.2 in OTS-206-immunized animals (Fig. 3j and Extended Data Fig. 4o). Sera from OTS-206-immunized hamsters had high WT receptor binding domain (RBD)-specific antibodies (Extended Data Fig. 4p) and neutralizing capacity against both WT^D614G^ and Omicron BA.2 (Extended Data Fig. 4q). Despite transmission to direct contacts, OTS-206 immunization provided protection from pulmonary atelectasis (Extended Data Fig. 4r,s), with minimal virus antigen in lung samples (Extended Data Fig. 4t,u). In conclusion, OTS LAV immunization protected K18-hACE2 mice and hamsters against lethal SARS-CoV-2 variants, eliciting neutralizing antibodies and CD8^+^ T cell responses. OTS4–5 and OTS7–8 reduced viral loads and prevented mortality and morbidity in hamsters but did not halt transmission to contacts. OTS-206 offered superior protection against weight loss, pulmonary issues and viral replication, yet transmission to contacts persisted.

### OTS-206 provides lasting immunity and rapid virus clearance

**Fig. 4:**
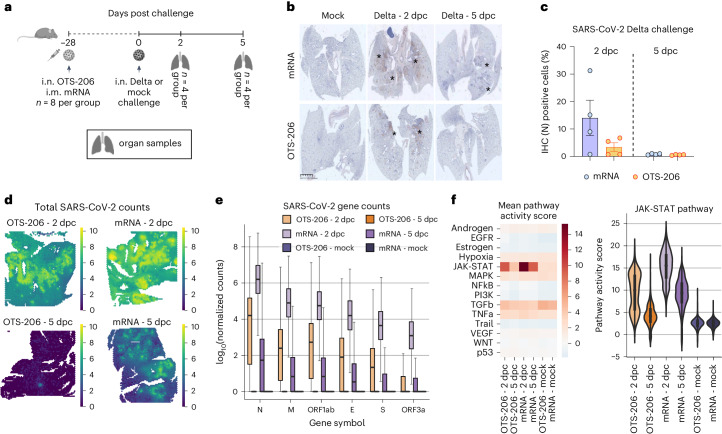
OTS-206 induces comparable efficacy to mRNA vaccines and superior virus clearance after challenge infection. **a**, Short-term experimental setup: age-matched K18-hACE2 mice were vaccinated with Spikevax mRNA vaccine (intramuscularly, i.m.) or OTS-206 (intranasally) (*n* = 8 mice per group) and later challenged with SARS-CoV-2 Delta VOC. Lungs were collected at 2 (*n* = 4) or 5 dpc (*n* = 4). Mock control group was vaccinated with culture medium. Data obtained from one experiment. Overview created with BioRender.com. **b**, Immunohistochemistry for SARS-CoV-2 nucleocapsid protein (N) of lung sections. **c**, Quantification of nucleocapsid-stained lung cells of *n* = 4 independently inoculated mice (bars indicate mean ± s.e.m.). **d**,**e**, Summed (**d**) and normalized (**e**) SARS-CoV-2 gene counts (N, ORF1ab, M, E, S, ORF3a). Both IHC and gene count quantification indicate faster clearance of SARS-CoV-2 for the OTS-206 group. **f**, Increased JAK-STAT pathway activity post challenge (left), with highest activity for the mRNA-vaccine group at 2 dpc, based on the mean pathway activity score (right) of *n* = 4 independently inoculated mice per group. Box and violin plots in **e** and **f** show minimum, maximum, mean (line) and s.d. Source data

**Extended Data Fig. 5:**
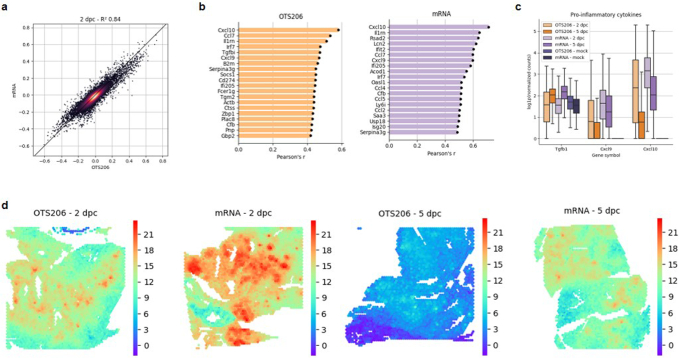
Spatial transcriptomics shows that OTS-206 vaccination induces similar activation of genes related to the immune response to viral infection and reduced inflammatory response. Short term experimental setup: Age-matched K18-hACE2 mice vaccinated with Spikevax mRNA-vaccine (intramuscularly) or OTS-206 (intranasally) (n=8 mice/group). Challenge with SARS-CoV-2 Delta VOC, lung harvest at 2- (n=4) or 5-days (n=4) post challenge (dpc). Mock control group was vaccinated with culture medium. Spatial transcriptomics samples (n=11): OTS 2dpc – (n=2), OTS 5dpc (n=2), mRNA 2dpc (n=2), mRNA 5dpc (n=3), mRNA mock (n=1), OTS mock (n=1). **a**, Pearson’s correlation coefficients were calculated between total SARS-CoV-2 gene counts and all host genes to determine spatial correlations. These values are plotted against each other on the x and y axis for the OTS and mRNA 2 dpc samples to show that the spatial gene expression signatures are very similar, as their correlation coefficients are nearly identical. **b**, Top 20 spatially most correlated genes in the lungs of infected mice vaccinated with OTS-206 or mRNA vaccine. **c**, Changes in proinflammatory cytokine expression between conditions (n=4/group). **d**, Spatial JAK-STAT pathway activity in the lung. We can see the co-occurrence between SARS-CoV-2 transcripts from **d**, and the increased JAK-STAT activity. Source data

**Extended Data Fig. 6:**
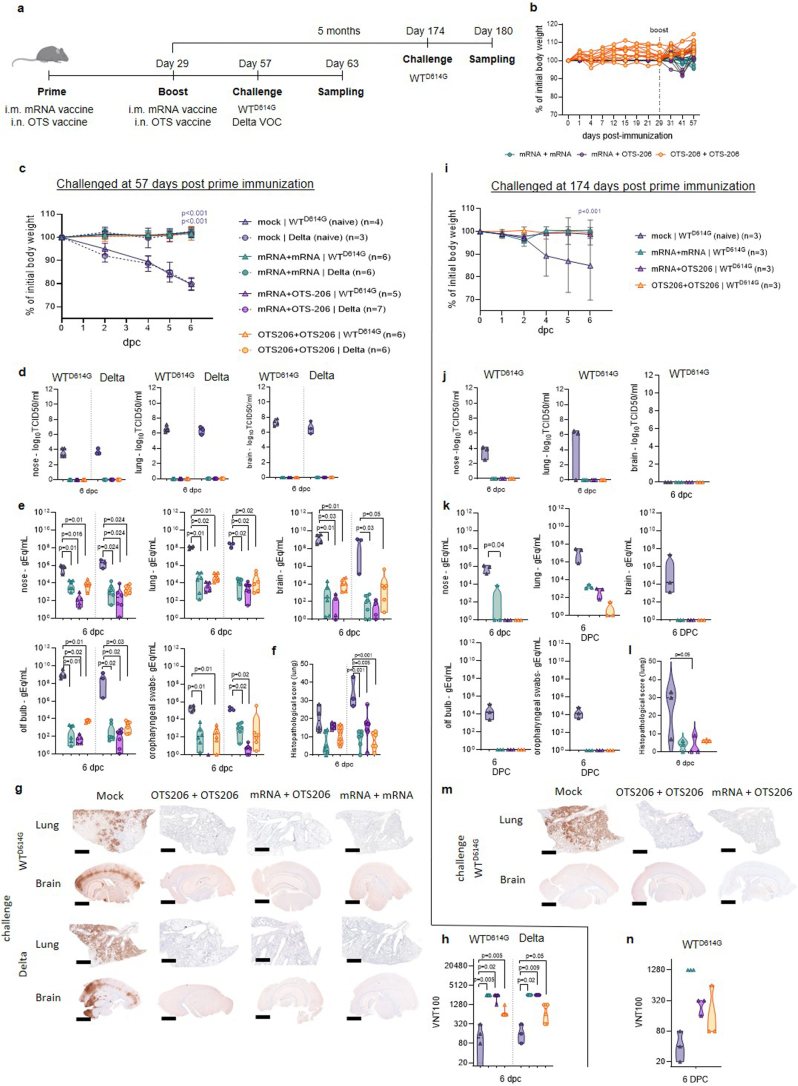
OTS-206 show comparable efficacy to mRNA-vaccines and inducing long-term immunity in K18-hACE2 mice. **a**, K18-hACE2 transgenic mice (7–15 weeks old, n=8 mice/group) were immunized (prime & boost) either intramuscularly with a single dose of 1 μg of mRNA-Vaccine Spikevax (Moderna), or intranasally with 5’000 PFU of OTS-206. **b**, During immunization, mice were regularly monitored for body weight changes. Each line in the body weight loss graphs represents a mouse. Six days post-challenge, mice were euthanized and organ samples were collected for evaluation of infectious virus titers, viral genome copy numbers, and pathology. At 57 dpi a group of mice was intranasally inoculated with 10^4^ TCID_50_ of SARS-CoV-2D614G, or SARS-CoV-2 Delta VOC (**c-h**). The rest of the immunized mice were kept for approximately 5 months and then intranasally inoculated with 10^4^ TCID_50_ of SARS-CoV-2^D614G^ (**i-n**). **d, j**, Infectious virus titers from the brain samples were determined using plaque assays in VeroE6 cells. **e, k**, Genome copy numbers (genome equivalence per ml, gEq/mL) in nose, lung, brain, olfactory bulb, and oropharyngeal swab samples of mice infected with different viruses were quantified using probe-specific RT–qPCR. **f, l**, High level of long-term protection was confirmed by histopathological scores for lung pathology. Data obtained from one experiment. **g, m**, Immunohistochemical analysis specific for SARS-CoV-2 nucleocapsid protein (magnification 50x). Scale bar, 500 µm. **h, n**, Sera collected on 6 dpc (post-challenge) were tested against SARS-CoV-2 Wuhan WT virus in a serum neutralization test. Data obtained from one experiment. Infectious viral particle concentrations, genome copies, immunohistochemical analysis and individual body weights in Extended Data Fig. 6 and Supplementary Fig. 6e and f**)**. Body weight changes, clinical scores, and histopathological scores in Supplementary Table 6. Statistical significance was determined using two-sided, two-way ANOVA (Tukey’s multiple comparison test) (panels c, and i,) or ordinary one-way ANOVA (panels f, and l,) or by using two-sided, unpaired nonparametric t-test (Mann Whitney test) (panels d, e, j and k). No asterisk indicates no statistical significance. *P<0.05, **P<0.01, ***P<0.001, ****P<0.0001. Source data

We tested K18-hACE2 mice with SARS-CoV-2 variant of concern (VOC) Delta (B.1.617.2) 28 days after a single dose of mRNA vaccine (Spikevax) or OTS-206 (Fig. 4a). Lung samples at 2 or 5 days post challenge (dpc) showed varied nucleocapsid protein (N) levels, higher in mRNA-vaccinated mice at 2 dpc, nearly absent in both at 5 dpc (Fig. 4b,c). Lung spatial transcriptomics confirmed these findings, showing greater viral mRNA expression in mRNA-vaccinated mice compared with OTS-206-vaccinated mice (Fig. 4d). Remarkably, SARS-CoV-2 transcripts were lower in OTS-206-vaccinated mice at 2 dpc versus mRNA-vaccinated mice and absent at 5 dpc in OTS-206-vaccinated mice (Fig. 4d,e), indicating quicker virus clearance in OTS-206-vaccinated mice. Spatial host gene expression near infection sites in lungs of top 100 genes in pathways such as MAPK, JAK-STAT, TGF-β and TNF-α were compared (Fig. 4f). A consistent spatial correlation pattern between viral and host genes in infected lungs for both mRNA and OTS-206 groups was noted at 2 dpc (Extended Data Fig. 5a). mRNA and OTS-206 groups shared 8 of the top 20 host genes highly correlated with virus RNA transcripts, implying comparable activation responses with both immunizations (Extended Data Fig. 5b). Pro-inflammatory cytokine expression post challenge, elevated in mRNA-vaccinated versus OTS-206 groups, mirrored the findings in SARS-CoV-2 patients^16,17^ (Extended Data Fig. 5c). Notably, the JAK-STAT pathway, vital in immune responses and tissue repair, exhibited significantly increased activity at infection sites (Extended Data Fig. 5d). As depicted in the violin plots illustrating pathway score distributions in each capture spot, JAK-STAT pathway activation at 2 dpc was higher in mRNA-vaccinated mice than in OTS-206-vaccinated mice (Fig. 4f). Remarkably, by 5 dpc, JAK-STAT activation reverted to nearly baseline levels in OTS-206-vaccinated mice. These findings indicate that quicker clearance of heterologous SARS-CoV-2 VOC Delta accompanies faster resolution of virus-induced host responses in K18-hACE2 mice. Subsequently, K18-hACE2 mice were immunized with either homologous or heterologous prime–boost combinations of mRNA vaccine (Spikevax) or OTS-206. Immediate protection was compared by challenging mice with WT^D614G^ or Delta VOC (B.1.617.2) at 28 days post boost, while long-term protection was assessed by challenging mice at 5 months post boost (mpb) with WTD614G virus (Extended Data Fig. 6a,b). All immunized mice, regardless of the vaccination combination or challenge virus, remained protected from disease and weight loss when challenged at 28 days or 5 mpb (Extended Data Fig. 6c,i and Supplementary Fig. 6e,f). No infectious virus was detected at 6 dpc in nose or lung samples from immunized animals (Extended Data Fig. 6d,j). Naïve WT^D614G^ and Delta VOC-challenged mice exhibited similar viral titres (Extended Data Fig. 6d), but Delta-challenged mice showed significantly higher lung histopathological scores compared with WT^D614G^-challenged mice (Extended Data Fig. 6f). Viral RNA load in organ samples and oropharyngeal swabs of all immunized groups demonstrated a significant reduction in replication compared with naïve controls challenged with either WT or Delta VOC (Extended Data Fig. 6e), with no infectious virus detected in the brain (Extended Data Fig. 6d). Notably, mice challenged at 5 mpb had less viral RNA in organ samples than those challenged at 57 days post prime immunization (Extended Data Fig. 6k). In addition, no infectious virus was found in the brain at 6 dpc (Extended Data Fig. 6j), indicating sustained protection over 5 months, supported by serology (Extended Data Fig. 6h,n). This pattern was also seen in lung histopathological scores (Extended Data Fig. 6f,l,g,m). Overall, these findings demonstrate OTS-206’s ability to provide long-term protection against SARS-CoV-2 in the highly sensitive K18-hACE2 mouse model, comparable to an established mRNA vaccine.

### Cleavage site deletion optimizes safety profile

**Fig. 5:**
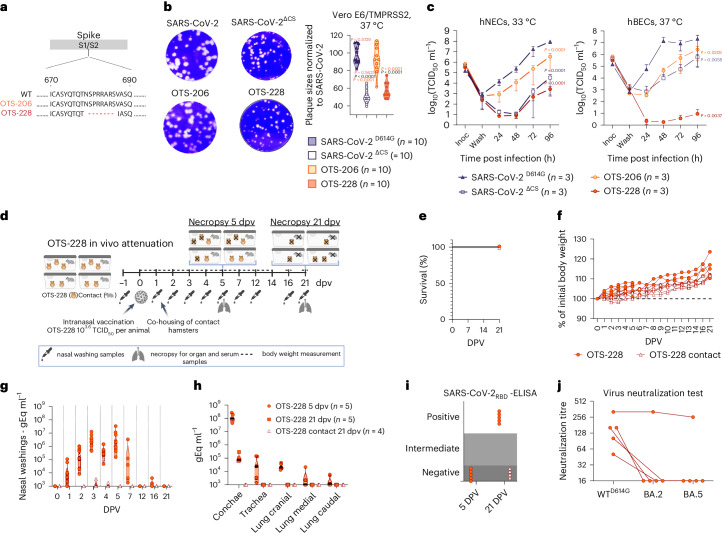
OTS-228 is significantly attenuated in vitro and not transmitted in vivo*.* **a**, Schematic representation of the deleted polybasic cleavage site in OTS-228 spike compared to WT and OTS-206. **b**, Reduced plaque sizes were observed with PCS deletion (*n* = 10 plaques measured per group). **c**, Infection of hNECs and hBECs with indicated viruses (*n* = 3 biologically independent samples, one experiment). Infectious particle titres were assessed over time, confirming attenuation of OTS-228 in both cell lines. Data are presented as mean ± s.e.m. **d**, Attenuation experiment in Syrian hamsters (*M. auratus*, male, 9 weeks old) with OTS-228 over 21 dpv. Data obtained from OTS-228-inoculated hamsters (*n* = 10) and naïve contact animals (*n* = 4) from one experiment. Overview created with BioRender.com. **e**,**f**, Full attenuation of OTS-228 in terms of survival (**e**) and body weight changes (**f**) of vaccinated and contact hamsters (1–5 dpv *n* = 10; 6–21 dpv *n* = 5; 1–21 dpv *n* = 4 contacts). **g**, OTS-228 was not transmitted to naïve direct contact hamsters (0, 1, 2, 3, 4, 5 dpv *n* = 10; 7, 12, 16, 21 dpv *n* = 5; 0, 2, 3, 4, 5, 7, 12, 16, 21 dpv *n* = 4 contacts). **h**, High genome loads were detected in conchae samples of the inoculated animals at 5 dpv but low genome loads in the lung samples, especially at later timepoints. **i**,**j**, Analysed serum samples confirmed lack of transmission to contacts (**i**), highlighting a partly cross-neutralizing antibody response (**j**). Statistical significance was assessed using two-sided, two-way ANOVA with Tukey’s multiple-comparisons adjusted *P* values (95% CI) (**b** and **c**). Violin plots in **b**, **g** and **h** show individual samples with mean values (middle lines). Source data

**Extended Data Fig. 7:**
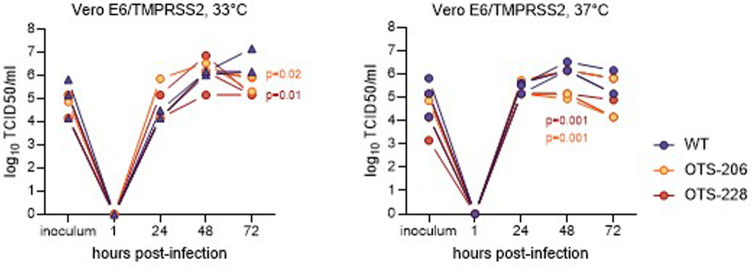
OTS-228 shows replication kinetics comparable to WT and OTS-206 in VeroE6/TMPRSS2 cells. Vero E6/TMPRSS2 cells were infected with 0.1 MOI of the indicated viruses and incubated at 37 °C for 1 h. After 1 h, supernatant was discarded and the cells were washed 3 times with PBS, and the third wash was kept for analysis. Following the addition of new sera on the cells, they were incubated at 37 °C. Samples were collected on designated time points post-infection. Infectious particle titers were assessed by TCID50 assays on VeroE6/TMPRSS2 cells. Each line in the graphs shows one replicate of samples. Statistical significances in the titer differences of OTS viruses vs WT on given times were determined using two-sided two-way ANOVA and p-values were adjusted using Tukey’s multiple-comparison test; *P<0.05, **P<0.01, ***P<0.001, ****P<0.0001. Source data

The OTS-206 vaccine was attenuated and effective against various SARS-CoV-2 variants but still transmissible. To mitigate this, we developed OTS-228 by removing the PCS of the spike protein (Fig. 5a). Removing the PCS led to smaller plaque sizes in Vero E6/TMPRSS2 cells (Fig. 5b). Replication kinetics in Vero E6/TMPRSS2 cells were similar to WT (Extended Data Fig. 7) but delayed in hNECs and showed lower titres in hBECs (Fig. 5c). Immunogenicity assessment of OTS-228 in K18-hACE2 mice demonstrated robust antibody (IgG subtypes and IgA) and CD8^+^ T cell responses after 14 days (Supplementary Fig. 3a).

OTS-228 vaccination resulted in significantly higher levels of IgG1 and IgA antibodies compared with mock controls, both in sera and bronchoalveolar lavage fluid (BALF) (Supplementary Fig. 3b). SARS-CoV-2-specific IgA concentrations in sera ranged from 20 to 250 ng ml^−1^, higher than in BALF (highest 50 ng ml^−1^). Furthermore, vaccinated mice showed elevated levels of SARS-CoV-2-spike-specific CD8^+^ T cells (Supplementary Fig. 3d). These findings indicate robust antibody- and CD8^+^ T cell-mediated responses in K18-hACE2 mice upon OTS-228 vaccination.

OTS-228’s transmission potential was tested by vaccinating 10 hamsters and adding 4 naive contacts at 1 day post vaccination (dpv) (Fig. 5d). No mortality or weight loss was observed in either group (Fig. 5e,f). Although viral RNA was found in vaccinated hamsters’ nasal washings for up to 7 days, in contact animals it remained minimal (Fig. 5g). Organ samples from vaccinated hamsters at 5 and 21 dpv showed low viral load in lungs and comparable levels to WT in conchae samples (Extended Data Fig. 3f and Fig. 5h). Deep sequencing of late conchae samples confirmed genetic stability of the OTS-228 modifications (Supplementary Data Table 1). No viral genome was found in organ samples of naive contact animals at 21 dpv (Fig. 5h).

Serological assessment confirmed seronegativity of contact animals after 20 days of contact (Fig. 5i). Sera of vaccinated animals displayed WT SARS-CoV-2 neutralizing capacity; one even neutralized Omicron variants (Fig. 5j). Lung histopathology at 5 dpi revealed no pneumonia-related atelectasis or SARS-CoV-2 lesions (Supplementary Fig 2a–e). Some showed mild pulmonary interstitial expansion with macrophages and one had perivascular immune cell infiltration. The findings show OTS-228’s complete attenuation and its ability to induce a broad neutralizing humoral immune response in Syrian hamsters. Crucially, transmission to naïve direct contacts was entirely prevented, addressing a previous concern with OTS-206.

### OTS-228 induces sterilizing, broad and mucosal protection

**Fig. 6:**
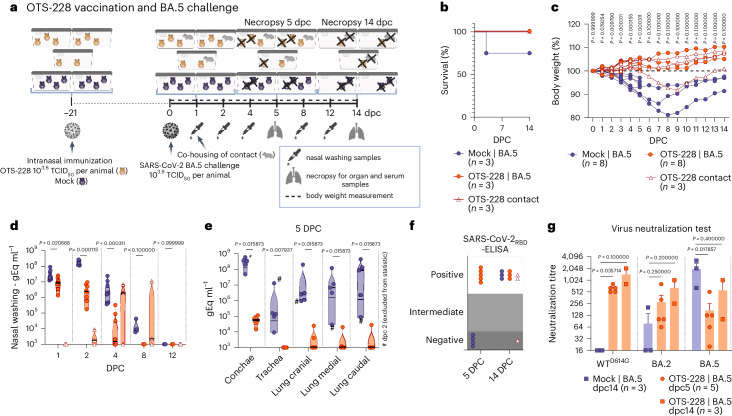
OTS-228 cross-protects against SARS-CoV-2 BA.5 challenge infections and limits transmission. **a**, Omicron BA.5 challenge infection of OTS-228-vaccinated Syrian hamsters (*M. auratus*, male, 12 weeks old). Data obtained from *n* = 8 hamsters per group (OTS-228 and mock) and *n* = 3 naïve contact animals (direct contact to OTS-228 group) from one experiment. Overview created with BioRender.com. **b**,**c**, Mortality (**b**) and body weight loss (**c**) were prevented by OTS-228 vaccination; OTS-228 group (1–5 dpc *n* = 8; 6–14 dpc *n* = 3); mock group (1–2 dpc *n* = 8; 3–5 dpc *n* = 7; 6–14 dpc *n* = 3); OTS-228 contact animals (1–14 dpc *n* = 3). **d**,**e**, Shedding of Omicron BA.5 virus genome was significantly reduced in the OTS-228-vaccinated animals (**d**) (OTS-228 group (1, 2, 4 dpc *n* = 8; 8, 12 dpc *n* = 3), mock group (1, 2 dpc *n* = 8; 4 dpc *n* = 7; 8, 12 dpc *n* = 3), OTS-228 contact animals (1, 2, 4, 8, 12 dpc *n* = 3)) and in the respiratory organ samples (**e**) at 5 dpc (OTS-228 group *n* = 5, mock group *n* = 4 (samples from 2 dpc animal excluded)). Organ samples of the 14 dpc group (Supplementary Fig. 4) confirmed virus clearance at this timepoint in contrast to the mock group, which still exhibited virus genome in conchae and lung samples. **f**, Serological evaluation confirmed reduced transmission to naïve contact animals. **g**, Evaluation of the post-challenge humoral immune response showed broad neutralization capacity of OTS-228 against WT^D614G^, but also against Omicron BA.2 and BA.5, while control sera only reacted against Omicron BA.5 (bars indicate mean ± s.e.m.). Statistical significance was assessed using two-sided, unpaired, non-parametric multiple *t*-test with Mann–Whitney test (compared ranks, 95% CI) (**c**–**e** and **g**). Violin plots in **d** and **e** show individual samples with means (middle lines). Source data

**Extended Data Fig. 8:**
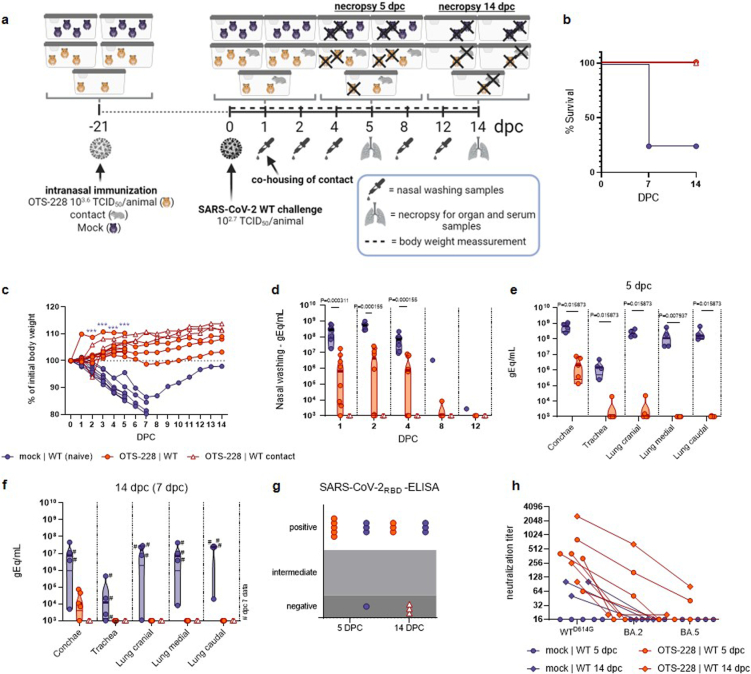
SARS-CoV-2 WT challenge infection of OTS-228 immunized Syrian hamsters. **a**, Experimental setup. Syrian hamsters (*Mesocricetus auratus*, male, 12 weeks old) were OTS-228 vaccinated and subsequently challenged by SARS-CoV-2 WT. Overview created with BioRender.com. **b**, Survival post-challenge infection. **c**, Relative body weight in percent. **d**, Virus genome copy numbers in nasal washing (until dpc5: n=8 OTS-228 and mock group, n=3 OTS-228 contact group; from dpc 6 on: n=3 OTS-228 and contacts and n=4 mock group) and **e**, organ samples of 5 dpc (n=5 OTS-228 group, n=4 mock group). **f**, Genome loads of organ samples taken 14 dpc (¾ mock vaccinated animal sampled on 7dpc) **g**, Serum samples of 5 and 14 dpc were analyzed by SARS-CoV-2 RBD-ELISA. **h**, Serum samples that reacted positively in the ELISA, were analyzed in addition by live virus neutralization assay (capacity to neutralize 100 TCID_50_) against ancestral (B.1) SARS-CoV-2 as well as against Omicron BA.2 and BA.5 variants. Statistical significance was assessed by two-sided, unpaired, nonparametric multiple t-test with Mann-Whitney test (compared ranks) (panel **c-e**). *P<0.05, **P<0.01, ***P<0.001, ****P<0.0001. No asterisk indicates no statistical significance. Source data

**Extended Data Fig. 9:**
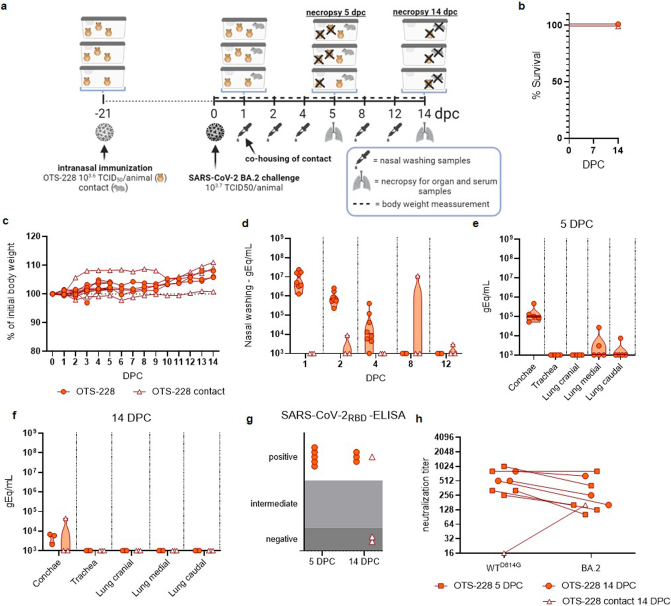
Omicron BA.2 challenge of OTS-228 vaccinated Syrian hamsters. **a**, Experimental setup. Syrian hamsters (*Mesocricetus auratus*, male, 12 weeks old) were OTS-228 vaccinated and subsequently challenged by SARS-CoV-2 BA.2. Data obtained from n = 8 OTS-228 inoculated hamster from one experiment, with n=3 naïve direct contact hamsters. Overview created with BioRender.com. **b**, Survival post challenge infection. **c**, Relative body weight in percent. Shown are the mean with standard error. **d**, Virus genome copy numbers in nasal washing (until dpc5 n=8 and n=3 (contacts), from dpc6 on n=3 for both groups) and **e**, organ samples of 5 dpc (n=5 OTS-228) and **f**, 14 dpc (n=3 OTS-228 and contacts). **g**, Serum samples of 5 and 14 dpc were analyzed by SARS-CoV-2_RBD_-ELISA. **h**, Serum samples which reacted positively in the ELISA, were analyzed in addition by live virus neutralization assay (capacity to neutralize 100 TCID_50_) against ancestral (B.1) as well as against Omicron BA.2 variant. Source data

**Extended Data Fig. 10:**
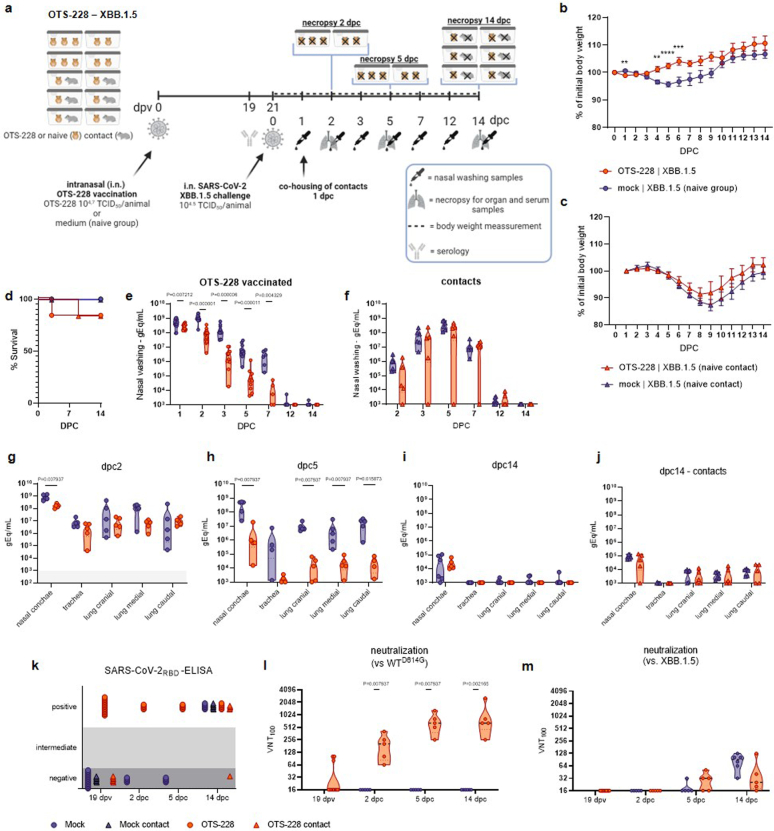
Syrian hamsters' shows protection against XBB.1.5 Omicron challenge infection after a single intranasal OTS-228 vaccination. **a**, Experimental setup. Syrian hamsters (*Mesocricetus auratus*, male and female equally mixed, 9 weeks old) were OTS-228 vaccinated and subsequently challenged by SARS-CoV-2 XBB.1.5, with co-housing of naive direct contact animals 1-day post-challenge (dpc). Data were obtained from n=16 hamsters/group, with n=6 naïve direct contact hamsters/group, comparing vaccinated to nonvaccinated. Overview created with BioRender.com. **b**, Body weight percentages of donor animals over time indicated no weight loss post-infection (dpc1-2: n=16/group, dpc3-5: n=11/group, dpc6-14 n=6/group). Data are presented as mean values +/- SEM. **c**, Body weight percentage of contact animals over time showed weight loss following co-housing (n=6). **d**, Relative survival post-challenge (n=6/group), with one unrelated death and its contact animal excluded from comparisons (see Supplementary Fig. 7). **e**, RT-qPCR analysis of donor hamster nasal washing samples for SARS-CoV-2 viral genomes revealed reduced shedding for the OTS-228 vaccinated group (dpc1-2: n=16/group, dpc3-5: n=11/group, dpc6-14 n=6/group). **f**, RT-qPCR analysis of contact hamster nasal washing samples for SARS-CoV-2 viral genomes indicated positive results for most animals (n=6/group). **g**, RT-qPCR analysis for SARS-CoV-2 viral genomes of organ samples from donor animals euthanized at 2 dpc (n=5/group) showed reduced viral loads in OTS-228 vaccinated animals. **h**, RT-qPCR analysis for SARS-CoV-2 viral genomes of organ samples from donor animals euthanized at 5 dpc (n=5/group) demonstrated pronounced virus clearance in the OTS-228 group. **i**, RT-qPCR analysis for SARS-CoV-2 viral genomes of organ samples from donor hamsters euthanized at 14 dpc (n=6/group) showed reduced viral loads in vaccinated animals. **j**, RT-qPCR analysis for SARS-CoV-2 viral genomes of organ samples from contact hamsters euthanized at 14 dpc (n=6/group) revealed no differences between groups. **k**, SARS-CoV-2RBD-ELISA of serum from donor and contact animals confirmed positive reactions in all vaccinated animals’ post-vaccination. **l**, Virus neutralization test (VNT100) against homologous WT SARS-CoV-2 verified detectable neutralization at 19 dpv and post-challenge (dpv19: n=16/group, dpc2: n=5/group, dpc5: n=5/group, dpc14: n=6/group). **m**, Virus neutralization test (VNT100) against SARS-CoV-2 variant Omicron XBB1.5 showed lower and delayed neutralizing immune responses compared to the ancestral virus. Statistical significance was assessed by two-sided, unpaired, nonparametric multiple t-test with Mann-Whitney test (compared ranks) (panel **b**, **c**, **e-j**, **l** and **m**). *P<0.05, **P < 0.01, ***P<0.001, ****P<0.0001. No asterisk indicates no statistical significance. Source data

Since OTS-228 has an optimal safety profile, we tested its single-dose protection in Syrian hamsters against WT SARS-CoV-2 (Extended Data Fig. 8a) and several VOCs (Omicron BA.2 (Extended Data Fig. 9a), Omicron BA.5 (Fig. 6a) and Omicron XBB.1.5 (Extended Data Fig. 10a)). Post challenge, vaccinated hamsters were co-housed with unvaccinated contacts. OTS-228 vaccination protected hamsters from lethal SARS-CoV-2 WT infection (Extended Data Fig. 8b), weight loss (Extended Data Fig. 8c) and reduced viral shedding (Extended Data Fig. 8d). Organ viral loads were significantly lower (Extended Data Fig. 8e,f). Remarkably, WT virus transmission to contacts was prevented (triangles in Extended Data Fig. 8b–d,f), confirmed by serology testing (Extended Data Fig. 8g,h). Omicron BA.2 challenge did not cause mortality (Extended Data Fig. 9b) or weight loss (Extended Data Fig. 9c). Virus shedding (Extended Data Fig. 9d) and replication in lungs were lower (Extended Data Fig. 9e) than in unvaccinated animals (Fig. 3i,j). No viral genome was detected at 14 dpc (Extended Data Fig. 9f). Only one contact animal showing evidence of infection (Extended Data Fig. 9g,h) highlight the reduced transmission. Vaccinated and challenged animals had similar neutralizing titres against WT^D614G^ and Omicron BA.2 (Extended Data Fig. 9h). Following Omicron BA.5 challenge (Fig. 6a), OTS-228-vaccinated animals showed no mortality or weight loss. However, one control animal did not regain consciousness and passed away during sampling (Fig. 6b,c). XBB.1.5-challenged animals (Extended Data Fig. 10a) were protected against weight loss (Extended Data Fig. 10b). Yet, one donor and contact animal were found dead at 3 and 9 dpc, respectively (Extended Data Fig. 10d). At least in the donor animal, PCR and pathologic evaluation suggested that it was unrelated to infection and it was therefore excluded from further analysis (Supplementary Fig. 7). Viral loads in nasal washings of the OTS-228 group were lower following BA.5 (Fig. 6d) and XBB.1.5 challenge (Extended Data Fig. 10e). Viral loads in organs and conchae were also reduced in vaccinated animals challenged with BA.5 (Fig. 6e) or XBB.1.5 (Extended Data Fig. 10g,h). Lung samples at 14 dpc tested negative for SARS-CoV-2 BA.5 (Supplementary Fig. 4) and XBB.1.5 (Extended Data Fig. 10i). Serological evaluation confirmed SARS-CoV-2-RBD-specific antibodies in the vaccinated group before challenge (Extended Data Fig. 10k) and at 2 and 5 dpc, unlike the mock group until 14 dpc (BA.5 (Fig. 6f) and XBB.1.5 (Extended Data Fig. 10k)). Rapid onset of cross-neutralizing humoral immune response was observed (BA.5 (Fig. 6g) and XBB.1.5 (Extended Data Fig. 10l,m)). Histopathological examination of lungs post OTS-228 vaccination demonstrated protection against pneumonia-related atelectasis with various challenge viruses (Supplementary Fig. 2). Nonetheless, sporadic SARS-CoV-2-typical lesions were observed depending on the virus type. Both BA.5 and XBB.1.5 were transmitted from OTS-228-vaccinated animals to naive direct contacts. After BA.5 challenge, 2 of 3 contacts became infected (Fig. 6d,f and Supplementary Fig. 4). With XBB.1.5 challenge, 5 of 6 contacts were infected (Extended Data Fig. 10f,j,k). To evaluate the role of the mucosal immune response in protection, we analysed 5 dpc conchae and lung samples from OTS-228-vaccinated hamsters challenged with WT (Extended Data Fig. 8e), BA.5 (Fig. 6e) or XBB.1.5 (Extended Data Fig. 10h) for SARS-CoV-2-specific IgA antibodies. All vaccinated groups showed IgA antibodies in both conchae and lung samples, contrasting with mock-vaccinated animals (Supplementary Fig. 5a,b). Vaccinated animals exhibited significantly more neutralizing antibodies in conchae (Supplementary Fig. 5c), lungs (Supplementary Fig. 5d) and sera (Supplementary Fig. 5e) than the mock-vaccinated controls.

Overall, single-dose OTS-228 application was proven to be safe and highly effective, providing protection against WT and Omicron BA.2, BA.5 and XBB.1.5 variants. It prevented WT SARS-CoV-2 transmission, demonstrating sterilizing immunity. Transmission of Omicron variants to contacts was also reduced. OTS-228 induced neutralizing antibodies and SARS-CoV-2-specific IgA in nasal and lung tissues just 5 days post infection.

## Discussion

In this study, we described an innovative application of the ‘one-to-stop’ strategy to attenuate SARS-CoV-2 to produce safe and effective LAV candidates^2^. By introducing synonymous changes into specific codons, we increased the likelihood of generating nonsense mutations into the viral genome (Fig. 1a), leading to reduced viral fitness and efficient attenuation^2^. These modifications had no impact on the amino acid sequence of the viral proteins but made the viruses less fit in primary human airway models (Fig. 1d,e). We demonstrated that the level of attenuation was adjustable by enriching genome regions with additional one-to-stop codons. Through stepwise modifications, we achieved significant attenuation in K18-hACE2 mice, resulting in 100% survival in a lethal SARS-CoV-2 animal model (Fig. 2a–j) and marked attenuation in the Syrian hamster model (Extended Data Fig. 3). Furthermore, we disarmed the virus by deleting ORF6–8 and the functional knockout of specific viral genes (Nsp1:K164A/H165A) known to interfere with antiviral cellular responses (Fig. 1a)^18–23^.

The resulting LAV candidate, OTS-206 displayed optimal efficacy in animal models, protecting against WT SARS-CoV-2, Delta (Figs. 2 and 4) and Omicron BA.2 variant (Fig. 3f–j). A single-dose OTS-206 provided faster Delta variant clearance compared with mRNA vaccines, resolving innate immune responses more rapidly (Fig. 4). Notably, protection was sustained for at least 5 months (Extended Data Fig. 6), so far not shown for other live-attenuated vaccine candidates^24–26^, and was comparable to mRNA vaccines, indicating its potential as an efficient immunity booster.

To further improve the vaccine candidate, we deleted PCS (PRRAR), resulting in final candidate OTS-228 (Fig. 5a), which displayed reduced plaque size and growth in vitro (Fig. 5b,c) while still stimulating humoral and cellular immunity effectively (Supplementary Fig. 3). Unlike other LAVs, which focus solely on either codon pair deoptimization^12,24–26^ or full sequence deletion^27,28^, OTS-228 combines multiple attenuation strategies, including OTS recoding, targeted deletions (ORF6–8, PCS) and inhibition of Nsp1 activity (Fig. 1a). This comprehensive approach resulted in significant attenuation and complete transmission blockage in the Syrian hamster model (Fig. 5d–j). Genome stability was also confirmed through sequencing (Supplementary Table 5).

Importantly, a single intranasal OTS-228 dose provided robust protection against severe pathology, prevented lung virus replication and blocked transmission of the WT virus (Extended Data Fig. 8). It also reduced transmission of Omicron BA.2 (Extended Data Fig. 9), BA.5 (Fig. 6) and XBB.1.5 (Extended Data Fig. 10) variants, showcasing its broad efficacy against heterologous strains.

In some of the hamster experiments (Fig. 3a–e, Extended Data Fig. 8 and Fig. 6) contact animals for the mock-vaccinated and challenged groups were not included to reduce the number of animals with respect to the 3R principle. To our knowledge, there is no description of less than 100% SARS-CoV-2 transmission between Syrian hamsters in direct contact, co-housed at 1 dpi, irrespective of the variant tested; we therefore considered 100% transmission between serologically naïve Syrian hamsters as a scientific fact.

In addition, OTS-228 elicited SARS-CoV-2-specific IgG and IgA responses detected in sera, BALF and mucosal surface samples, as well as spike-specific CD8^+^ T cells establishing local humoral and cell-mediated immunity. In summary, OTS LAV candidates, OTS-206 and OTS-228, demonstrated optimal attenuation, protection and safety in preclinical animal models. The protection against WT and VOCs remained robust even 5 months post vaccination, as shown for OTS-206. With the deletion of PCS, OTS-228 lost its transmissibility but still induced protection against WT SARS-CoV-2 and emerging Omicron VOCs BA.2, BA.5 and XBB.1.5, as well as reducing their transmission capacity. These promising findings highlighting OTS-228’s potential as a LAV in protecting against and preventing SARS-CoV-2 transmission chains granted OTS-228 to advance to the clinical study phase.

## Methods

### Biosafety statement

All experiments with infectious SARS-CoV-2 variants as well as the attenuated OTS constructs were performed in enhanced biosafety level 3 (BSL3) containment laboratories at the Institute of Virology and Immunology (IVI), Mittelhäusern, Switzerland, and the Friedrich-Loeffler-Institut (FLI), Greifswald-Insel Riems, Germany. The standard operating procedures of BSL3 facilities were approved by relevant authorities in Switzerland and Germany. All personnel received relevant training before commencing work in BSL3 laboratories.

### Ethics statements for animal experimentation

All hamster experiments were evaluated by the responsible ethics committee of the State Office of Agriculture, Food Safety and Fishery in Mecklenburg–Western Pomerania (LALLF M-V) and gained governmental approval under registration number LVL MV TSD/7221.3-1-041/20. Mouse studies were approved by the Commission for Animal Experimentation of the Cantonal Veterinary Office of Bern and conducted in compliance with the Swiss Animal Welfare legislation under licence BE43/20.

### Cell culture

At IVI, Vero E6 (Vero C1008, ATCC) and Vero E6/TMPRSS2 cells (NIBSC Research Reagent Depository, UK) were cultured in Dulbecco’s modified Eagle’s medium (DMEM) supplemented with 10% (v/v) fetal bovine serum (FBS), 1% (w/v) non-essential amino acids (NEAA), 100 IU ml^−1^ penicillin and 100 μg ml^−1^ streptomycin. BHK-21 cells expressing the N protein of SARS-CoV (BHK-SARS-N)^29^ were grown in minimal essential medium (MEM) supplemented as DMEM above. Cells were maintained at 37 °C with 5% CO_2_ under selection with puromycin (Vero E6/TMPRSS2) and doxycyclin (BHK-SN).

At FLI, Vero E6 (Collection of Cell Lines in Veterinary Medicine CCLV-RIE 0929) were cultured using a mixture of equal volumes of Eagle MEM (Hanks’ balanced salts solution) and Eagle MEM (Earle’s balanced salts solution) supplemented with 2 mM l-glutamine, NEAA adjusted to 850 mg l^−1^, NaHCO_3_, 120 mg l^−1^ sodium pyruvate and 10% FBS at pH 7.2.

### Generation of infectious complementary DNA clones using transformation-associated recombination cloning and rescue of recombinant viruses

The in-yeast TAR cloning method, as previously described^15^, was used to generate recombinant OTS viruses of SARS-CoV-2. Briefly, 12 overlapping DNA fragments encoding the entire SARS-CoV-2 genome (referred to as WU-fragments 1–12), along with a TAR vector, were recombined in yeast as a yeast artificial chromosome (YAC). WU-fragments 2, 4, 5, 7 and 8 were recoded according to the OTS strategy to produce OTS fragments. The OTS strategy involves recoding all serine and leucine codons to synonymous codons that are just one nucleotide away from encoding a stop codon.

Initially, single OTS fragments were used to create infectious SARS-CoV-2 clones, namely, OTS2 (WU-fragment 2 out of the 12 WU-fragments was replaced with OTS Fragment 2), OTS4, OTS5, OTS7 and OTS8. Subsequently, clones with multiple OTS fragments were created, such as OTS4–5, OTS7–8 and OTS4-5-7-8. Supplementary Table 3 provides a detailed list of all nucleotide changes recoded in the OTS fragments. The recombinant SARS-CoV-2 OTS-206 infectious clone contains additional modifications, for which we created WU-fragment 2-Nsp1: K164A, H165A, and WU-fragment 11: delORF6–8. We introduced four-point mutations into WU-fragment 2 to create amino acid changes K164A and H165A in the Nsp1 gene, and deleted ORF6 to ORF8 from WU-fragment 11 using PCR. Lastly, to create OTS-228, the final iteration of our attenuation strategy, WU-fragment 10 was replaced with WU-fragment 10: delFCS, where the PCS in the SARS-CoV-2 spike was removed. The primers used for these modifications are listed in Supplementary Table 2. The YACs were cleaved by EagI digestion, and in vitro transcription was performed using the T7 RiboMAX Large Scale RNA production system (Promega), as previously described^15^. The resulting capped mRNA was electroporated into BHK-21 cells expressing the SARS-CoV N protein. Electroporated BHK-21 cells were then co-cultured with Vero E6/TMPRSS2 cells to produce passage 0 (p.0) of the recombinant viruses. To generate a p.1 virus stock for downstream experiments, the p.0 viruses were used to infect Vero E6/TMPRSS2 cells.

### Determination of infectious viral particles, plaque phenotype and foci sizes

A complete list of viruses used in this study can be found in Supplementary Table 1. Vero E6 or Vero E6/TMPRSS2 were used to culture viruses, and the identity of all virus stocks was verified by whole-genome next-generation sequencing. Infectious viral particle titres were determined using tissue culture infectious dose 50% (TCID_50_) measurement on Vero E6 or Vero E6/TMPRSS2 cells. Briefly, 2 × 10^4^ cells per well were seeded in a 96-well plate 1 day before the titration and then inoculated with a 10-fold serial dilution of the samples. Three to six technical replicates were performed for each sample. Cells were then incubated at 37 °C in a humidified incubator with 5% CO_2_. After 72 h, cells were fixed with 4% (v/v) buffered formalin (formafix) and stained with crystal violet. TCID_50_ was calculated according to the Spearman–Kaerber formula. The plaque sizes caused by the respective viruses in 6-well plates at 2 dpi were measured in Adobe Illustrator. Statistical significance was determined using ordinary one-way analysis of variance (ANOVA) and *P* values were adjusted using Tukey’s multiple-comparisons test; *P < 0.05, **P < 0.01, ***P < 0.001, ****P < 0.0001.

### Genetic stability of recombinant OTS viruses

To evaluate their genetic stability, OTS4–5 (10-times Vero E6), OTS7–8, (10-times Vero E6) and OTS-206 (15-times Vero E6/TMPRSS2) were passaged at low multiplicity of infection (MOI) (0.01) and sequenced by Ion Torrent sequencing. Also, conchae samples of OTS4–5 and OTS7–8 contact animals at 20 days post initial contact were sequenced. Results are shown in Supplementary Table 5.

### Ion Torrent sequencing

Virus stocks and animal samples were sequenced using a generic metagenomics sequencing workflow as described previously^30^ with some modifications. For reverse-transcribing RNA into cDNA, SuperScriptIV First-Strand cDNA Synthesis System (Invitrogen) and the NEBNext Ultra II Non-Directional RNA Second Strand Synthesis Module (New England Biolabs) were used, and library quantification was done with the QIAseq Library Quant Assay kit (Qiagen). Animal samples were treated with a myBaits panel (Daicel Arbor Biosciences) specific for SARS-CoV-2 as described^31^. Libraries were quality checked, quantified and sequenced using an Ion 530 chip and chemistry for 400 base pair reads on an Ion Torrent S5XL instrument (Thermo Fisher). Raw sequencing data were analysed using the Genome Sequencer Software Suite (v.2.6; Roche), applying default software settings for quality filtering and mapping. The obtained genome sequences were compared with their reference genomes via alignment using MAFFT v.7.38837 as implemented in Geneious v.10.2.3 (Biomatters; https://www.geneious.com). The variant analysis integrated in Geneious Prime 10.2.3 were applied (default settings, minimum variant frequency 0.02) to detect single nucleotide variants.

### Illumina sequencing

Sequencing reads were trimmed using TrimGalore v.0.6.5, and FastQC v.0.11.9 was used to assess overall read quality. Trimmed reads for each OTS sample were then aligned to their corresponding OTS reference sequence using Bowtie2 v.2.3.4. For virus stocks, consensus sequences were generated using Samtools v.1.10 with the -d option set to 10,000. For OTS passaged samples, nucleotide variants were called using Lofreq v.2.1.5 with the -C option set to 100 and the -d option set to 10,000. The resulting VCF files were filtered using the lofreq filter command for variants called at a frequency of ≥0.1. Data analysis was performed on UBELIX, the high-performance computing cluster at the University of Bern (http://www.id.unibe.ch/hpc).

### Virus replication kinetics, fluorouracil and molnupiravir treatment

The virus replication kinetics of the OTS viruses in comparison to WT SARS-CoV-2 were determined without any treatment, as well as under fluorouracil (5-FU) (Sigma, F6627) and molnupiravir (Lucerna Chem, HY-135853-10MG) treatment conditions. Vero E6/TMPRSS2 cells were infected with 0.1 MOIs of the WT SARS-CoV-2 or OTS viruses for 1 h. After 1 h, inoculum was removed, cells were washed three times with 1x PBS and new media were added to the cells. Supernatant from wells were collected at 6, 18, 24, 48 and 72 hpi for the infectious virus titre determination and diluted 1:1 with virus transport medium (VTM). For the antiviral treatment condition, Vero E6/TMPRSS2 cells were pretreated for 30 min with 5-FU and molnupiravir, and then infected with 0.1 MOI of WT SARS-CoV-2 and OTS4-5-7-8 for 1 h. Afterwards, inoculum was removed, cells were washed and new medium containing either 5-FU (concentration range of 40–280 μM) or molnupiravir (concentration range of 0.1–10 μM) was added to the cells for 24 h. After 24 h, supernatant from cells were collected and used to determine the virus titres. Infectious virus titres were assessed by standard TCID_50_ assays on Vero E6/TMPRSS2 cells, as explained above.

### Well-differentiated primary airway epithelial cells

Primary hBECs were isolated from lung explants and hNECs were obtained commercially (Epithelix Sàrl). The generation of well-differentiated hBECs and hNECs at the air–liquid interface (ALI) was based on a previously described method, with minor adjustments^32^. Human BECs/NECs were expanded in collagen-coated (Sigma) cell culture flasks (Costar) in PneumaCult ExPlus medium, supplemented with 1 μM hydrocortisone, 5 μM Y-27632 (Stem Cell Technologies), 1 μM A-83-01 (Tocris), 3 μM isoproterenol (Abcam) and 100 μg ml^−1^ primocin (Invivogen), and maintained at 37 °C and 5% CO_2_. Expanded hBECs/hNECs were seeded onto 24-well plate inserts with a pore size of 0.4 μm (Greiner Bio-One) at a density of 50,000 cells per insert, submerged into 200 μl of supplemented PneumaCult ExPlus medium on the apical side and 500 μl in the basolateral chamber. To induce the differentiation of the cells, PneumaCult ALI medium supplemented with 4 μg ml^−1^ heparin (Stem Cell Technologies), 5 μM hydrocortisone and 100 μg ml^−1^ primocin was added to the basolateral chamber. Basal medium was replaced every 2–3 days, and the cells were maintained at 37 °C and 5% CO_2_ until ciliated cells appeared and mucus was produced. After 3–4 weeks post exposure to ALI, hBECs/hNECs were considered well differentiated. For Fig. 1d, well-differentiated hNECs were obtained commercially (Epithelix Sàrl) and consist of a pool of 14 human donors each. Basal medium (Epithelix Sàrl) was replaced every 2–3 days and cells were maintained at 33 °C and 5% CO_2_. To remove mucus from hBECs and hNECs, cells were washed once a week with 250 μl of pre-warmed Hank’s balanced salt solution (HBSS, Gibco) for 20 min at 37 °C.

### Virus replication kinetics on human primary airway cells

Human BECs and NECs were infected with 0.1 MOI or 5 × 10^4^ plaque-forming units (p.f.u.s) of the OTS viruses listed or WT SARS-CoV-2 as described previously, with some changes^33^. Viruses were diluted in HBSS, applied apically and incubated for 1 h at 37 °C or 33 °C for hBECs or hNECs, respectively. Then, the inoculum was removed and the cells were washed three times with 100 µl of HBSS. The last wash was collected as the 1 hpi timepoint and diluted 1:1 with VTM. Afterwards, hBECs and hNECs were incubated in a humidified incubator with 5% CO_2_ at 37 °C or 33 °C, respectively. For quantification of infectious viral particle release 24, 48, 72 and 96 hpi, 100 µl of HBSS were applied to the apical surface 10 min before the respective timepoint, incubated and subsequently collected. Apical washes were diluted 1:1 with VTM and stored at −80 °C until further analysis. Infectious virus titres in the apical washes were assessed using a standard TCID_50_ assay on Vero E6/TMPRSS2 cells.

### Mouse studies

Well-characterized SARS-CoV-2 model hACE2-K18Tg mice (Tg(K18-hACE2)2Prlmn)^34,35^ were bred at the specific-pathogen-free facility of the Institute of Virology and Immunology and housed as previously described^36^. For infection, 7–16-week-old female and male mice were anaesthetized with isoflurane and inoculated intranasally with 20 μl per nostril (5,000 p.f.u.s per mouse). The mice were observed for clinical symptoms, weighed and swabbed at specific timepoints. The clinical symptoms were scored and the animals were euthanized before they reached the humane endpoint. On euthanasia day, oropharyngeal swabs, serum and organs samples were collected as mentioned in our previous studies^35^.

For the vaccination experiments, K18-hACE2 mice (7–16 weeks old) were vaccinated twice at a 4-week interval either intramuscularly with a single dose of 1 μg of mRNA vaccine Spikevax (Moderna) or intranasally with 5,000 p.f.u.s of OTS viruses. Four weeks after the boost, the vaccinated mice and a group of sex- and age-matched naïve animals were challenged intranasally with the challenge virus inoculum (either WT (BetaCoV/Wuhan/IVDC-HB-01/2019, Acc. No. MT108784), WT^D614G^ (BetaCoV/Germany/BavPat1/2020, Acc. No. EPI_ISL_406862) or Delta (hCoV-19/Germany/BW-FR1407/2021, Acc. No. EPI_ISL_2535433)) described in the results section. Euthanasia and organ collection were performed at 6 dpc as described above. All mice were monitored daily for weight loss and clinical signs. Oropharyngeal swabs were collected daily as previously described. Animals were randomly assigned to the various experimental groups and groups were handled equally to avoid any bias.

### Hamster studies

Specific-pathogen-free male Syrian golden hamsters (*Mesocricetus auratus*) of 4–12 weeks age were purchased from Janvier labs, Le Genest-Saint-Isle, France. Animals were randomly assigned to experimental groups and were handled equally to avoid any bias. Syrian hamsters received either 70 µl (35 µl into each nostril) of the respective OTS constructs (OTS4–5, OTS7–8, OTS-206 or OTS-228) intranasally or were challenged 3 weeks post immunization with SARS-CoV-2 WT (BetaCoV/Wuhan/IVDC-HB-01/2019, Acc. No. MT108784), SARS-CoV-2 Omicron BA.2 (SARS-CoV-2/human/NLD/EMC-BA2-1/2022, Acc. No. ON545852, kindly provided by B. Haagmans) or SARS-CoV-2 Omicron BA.5 (hCoV-19/South Africa/CERI-KRISP-K040013/2022, Acc. No. EPI_ISL_12268493.2, kindly provided by Alex Sigal). Details about the OTS viruses and challenge viruses are described in Supplementary Table 1. Body weight was tracked and nasal washing samples under short-term isoflurane anaesthesia were taken (flushing 200 µl PBS into each nostril and collecting the reflux into a 2 ml tube) at timepoints as specifically indicated for each experiment (Figs. 3a,f, 5d and 6a, and Extended Data Figs. 3a, 8a, 9a and 10a). To obtain organ samples (nasal conchae, trachea, lung caudal, medial and cranial lobes), animals were euthanized by an isoflurane overdose and subsequent decapitation. Serum samples were obtained during euthanasia by collecting the blood into serum-separating tubes (BD Vacutainer).

### Processing of animal specimens, viral RNA and infectious particle quantification

Organ samples of ~0.1 cm^3^ size from hamsters were homogenized in a 1 ml mixture composed of equal volumes of Hank’s balanced salts MEM and Earle’s balanced salts MEM containing 2 mM l-glutamine, 850 mg l^−1^ NaHCO_3_, 120 mg l^−1^ sodium pyruvate and 1% penicillin–streptomycin at 300 Hz for 2 min using a Tissuelyser II (Qiagen) and were then centrifuged to clarify the supernatant.

Nucleic acid was extracted from 100 μl of the hamster nasal washes after a short centrifugation step or from 100 μl of organ sample supernatant using the NucleoMag Vet kit (Macherey Nagel). Nasal washings, oropharyngeal swabs and organ samples from hamsters were tested using virus-specific RT–qPCR. The RT–qPCR reaction was prepared using the qScript XLT One-Step RT–qPCR ToughMix (QuantaBio) in a volume of 12.5 µl including 1 µl of the respective FAM mix and 2.5 µl of extracted RNA. The reaction was performed for 10 min at 50 °C for reverse transcription, 1 min at 95 °C for activation and 42 cycles of 10 s at 95 °C for denaturation, 10 s at 60 °C for annealing and 20 s at 68 °C for elongation. Fluorescence was measured during the annealing phase. RT–qPCRs were performed on a Bio-Rad real-time CFX96 detection system (Bio-Rad). The primers are listed in Supplementary Table 2.

Organ samples from mice were either homogenized in 0.5 ml of RA1 lysis buffer supplemented with 1% β-mercaptoethanol and later used for RNA isolation, or in gentleMACS M-tubes containing 1 ml DMEM (Miltenyi Biotec) for the detection of infectious particles as previously described^36^. RNA was isolated using the NucleoMag Vet kit (Macherey Nagel). The RT–qPCR reaction was prepared using *Taq*Path 1-Step Multiplex Master Mix kit (Thermo Fisher) with primers and probes targeting the SARS-CoV-2 E gene, and was performed for 10 min at 45 °C for reverse transcription, 10 min at 95 °C for activation and 45 cycles of 15 s at 95 °C for denaturation, 30 s at 58 °C for annealing and 30 s at 72 °C for elongation. Fluorescence was measured during the annealing phase. RT–qPCRs were performed on a Bio-Rad real-time CFX96 detection system (Bio-Rad). The primers are listed in Supplementary Table 2. Infectious virus titres were determined by TCID_50_ measurement on Vero E6 cells and were calculated according to the Spearman–Kaerber formula.

### Histopathological and immunohistochemical analysis in mice

The left lung and the left hemisphere of the brain from mice were collected into 4% formalin. After fixation, both tissues were embedded in paraffin, cut at 4 μm and stained with haematoxylin and eosin (H&E) for histological evaluation. Scoring of the lung tissue pathology was done according to a previously published scoring scheme^36^. Immunohistochemical (IHC) analysis of the lung and the brain was performed by using a rabbit polyclonal anti-SARS-CoV nucleocapsid antibody (Rockland, 200-401-A50) in a BOND RXm immunostainer (Leica Biosystems). For this purpose, paraffin blocks were cut at 3 μm, incubated with citrate buffer for 30 min at 100 °C for antigen retrieval and incubated with a 1:3,000 dilution of the first antibody for 30 min at room temperature. Bond Polymer Refine Detection Visualization kit (Leica Biosystems) was afterwards used for signal detection using 3,3′-diaminobenzidine as chromogen and counterstaining with haematoxylin.

### Histopathological and immunohistochemical analysis in hamster

The left lung lobe was carefully removed, immersion fixed in 10% neutral-buffered formalin, paraffin embedded, and 2–3-μm sections were stained with H&E. Consecutive sections were processed for IHC according to standardized procedures of the avidin-biotin-peroxidase complex (ABC) method. Briefly, endogenous peroxidase was quenched on dewaxed lung slides with 3% hydrogen peroxide in distilled water for 10 min at room temperature (r.t.). Antigen heat retrieval was performed in 10 mM citrate buffer (pH 6) for 20 min in a pressure cooker. Non-specific antibody binding was blocked for 30 min at r.t. with goat normal serum, diluted in PBS (1:2). A primary anti-SARS-CoV nucleocapsid protein antibody was applied overnight at 4 °C (Rockland, 200-401-A50, 1:3,000), and the secondary biotinylated goat anti-mouse antibody was applied for 30 min at r.t. (Vector Laboratories, 1:200). Colour was developed by incubation with ABC solution (Vectastain Elite ABC kit, Vector Laboratories), followed by exposure to 3-amino-9-ethylcarbazole substrate (AEC, Dako). The sections were counterstained with Mayer’s haematoxylin and cover slipped. As negative control, consecutive sections were labelled with an irrelevant antibody (M protein of Influenza A virus, ATCC clone HB-64). An archived control slide from a SARS-CoV2-infected Syrian hamster was included in each run. All slides were scanned using a Hamamatsu S60 scanner and evaluated using the NDPview.2 plus software (v.2.8.24, Hamamatsu Photonics) by a trained (T.B.) and a board-certified pathologist (A.B.), blind to treatment. The lung tissue was evaluated using a 500 × 500 μm grid, and the extent of pneumonia-associated consolidation was recorded as a percentage of affected lung fields. Further, the lung was examined for the presence of SARS-CoV-2-characteristic lesions described for hamsters, that is, intra-alveolar, interstitial, peribronchial and perivascular inflammatory infiltrates, alveolar oedema, necrosis of the bronchial epithelium, diffuse alveolar damage, vasculitis, activation of endothelium with immune cell rolling, as well as bronchial epithelial and pneumocyte type 2 hyperplasia. Following IHC, the distribution of virus antigen was graded on an ordinal scale with scores: 0, no antigen; 1, focal, affected cells per tissue <5% or up to 3 foci per tissue; 2, multifocal, 6%–40% affected; 3, coalescing, 41%–80% affected; 4, diffuse, >80% affected. The target cell was identified on the basis of morphology.

### Serological tests

To evaluate the virus neutralizing potential of hamster serum samples, a live virus neutralization test was done following an established standard protocol^37^. Briefly, sera were prediluted 1/16 in MEM and further diluted in log_2_ steps until a final tested dilution of 1:4,096. Each dilution was evaluated for its potential to prevent 100 TCID_50_ SARS-CoV-2 per well of the respective VOC from inducing cytopathic effect in Vero E6 cells, giving the virus neutralization titre (VNT_100_). The following SARS-CoV-2 variants were used for testing: SARS-CoV-2 WT^D614G^ (BetaCoV/Germany/BavPat1/2020, Acc. No. EPI_ISL_406862, kindly provided by Roman Wölfel), SARS-CoV-2 Omicron BA.2 (SARS-CoV-2/human/NLD/EMC-BA2-1/2022, Acc. No. ON545852, kindly provided by B. Haagmans) or SARS-CoV-2 Omicron BA.5 (hCoV-19/South Africa/CERI-KRISP-K040013/2022, Acc. No. EPI_ISL_12268493.2, kindly provided by Alex Sigal).

In addition, serum samples were tested using multispecies enzyme-linked immunosorbent assay (ELISA) for sero-reactivity against the WT SARS-CoV-2 RBD domain^38^.

Similarly, for mouse samples, serum was diluted initially at 1:20 with DMEM and subsequently further diluted to reach the final dilution of 1:2,560. Diluted sera were first incubated with the virus in a 1:1 volume ratio and after 1 h incubation, the serum–virus mixture was applied on Vero E6 cells in 96-well plates for 2–3 days of incubation. The serum dilution in which the cells were still intact was recorded as the neutralization titre of the serum for the given virus.

### SARS-CoV-2-specific IgA measurement by ELISA from organ homogenates

SARS-CoV-2-specific IgA was detected in the supernatant of homogenates from hamster conchae and lung using ELISA. ELISA plates (96-well, flat-bottom; Nunc MaxiSorp) were coated with 100 µl of 1.5 µg ml^−1^ recombinant SARS-CoV-2 spike protein (S1 + S2 ECD, His tag; Sino Biological) in PBS overnight at 4 °C. The following day, plates were washed three times with PBS supplemented with 0.05% Tween 20 (PBS-T) and incubated with 3% BSA in PBS-T (blocking buffer) for 1 h at r.t. to block unspecific binding. Organ homogenates were centrifuged at 4,000 × *g* for 5 min. Supernatants were diluted 3-fold from a starting dilution at 1:8 in blocking buffer before adding 50 µl of the diluted samples to the plates. Samples were incubated in the plates for 2 h at r.t., then washed three times before adding 50 µl of 1:50-diluted biotinylated anti-hamster IgA detection antibody (Brookwood Biomedical). Following a 2 h incubation at r.t., plates were washed three times and 50 µl of High-Sensitivity NeutrAvidin HRP conjugate was added for 30 min at r.t. The plates were washed three times and 50 µl of 1-Step Ultra TMB ELISA substrate solution (Thermo Fisher) was added. After 5 min, the reaction was stopped by adding an equal volume of 2 M sulfuric acid. The plates were read for absorbance at 450 nm and 570 nm on a Tecan Infinite M200 Pro microplate reader. Extinction at 570 nm was subtracted as background. The effective dilution to reach 50% of the maximal extinction (ED_50_) for each sample was determined using a four-parameter nonlinear regression curve fit in GraphPad Prism v.9.

### Detection of SARS-CoV-2 neutralizing antibodies in organ homogenates and sera

SARS-CoV-2 neutralizing antibodies were detected in conchae, lung homogenates and the correlating sera samples by using the cPass SARS-CoV-2 Neutralization Antibody Detection kit (GeneScript, REF L00847) according to manufacturer instructions. In brief, samples and controls were diluted 1:10 in sample dilution buffer and afterwards mixed 1:1 with HRP-conjugated SARS-CoV-2 RBD, followed by incubation for 30 min at 37 °C. Of the sample/RBD-HRP mixture, 100 µl was then added to a 96-well microtitre plate coated with hACE2 receptor proteins and incubated for 15 min at 37 °C. Subsequently, the microtitre plate was washed four times using 1x wash solution, followed by the addition of 100 µl TMB substrate solution and incubation for another 15 min at r.t. in the absence of light. The reaction was stopped by adding 50 µl stop solution into each well and the optical density (OD) was measured at 450 nm. Signal inhibition (%) was calculated as: (1 − OD_450 sample_/OD_450 negative control_) × 100%. Cut-off was defined as 30% inhibition.

### Tetramer staining of mice blood cells

All preparation of cells and staining were done under BSL3 conditions. Whole blood was collected in EDTA tubes with heparinized capillary tubes (Sigma-Aldrich, BR749311). After the centrifugation of the blood at 400 × *g* for 10 min, sera were collected, heat inactivated at 56 °C and immediately stored at −80 °C. In-house red blood cell lysis buffer (containing ammonium chloride, sodium bicarbonate, EDTA) was added to the rest of the blood and the mixture incubated on ice for 10 min. Later, cold PBS was added to the tubes, the tubes centrifuged at 350 × *g* at 4 °C for 5 min, then supernatant was discarded. Following the addition of Live/Dead fixable aqua dead cell stain (Thermo Fisher), cells were incubated on ice for 10 min, then washed with cold PBS and centrifuged at 350 × *g* at 4 °C for 5 min. After discarding the supernatant, cells were incubated with avidin (MERCK) and FcR-blocking reagent (anti-mouse CD16/32) (Miltenyi Biotec) for 20 min on ice. Subsequently, antibody mixes including the following antibodies were mixed with the cells and incubated for 30 min in the dark on ice: anti-mouse anti-CD8-FITC (Biolegend), anti-mouse anti-CD45-PerCP (Biolegend), anti-mouse anti-CD3e-PE (Biolegend), either MHC-I tetramer against SARS-CoV-2 spike (H-2K(b), SARS-CoV-2 S 539-546, VNFNFNGL) (NIH tetramer core facility) or negative control (H-2D(b) Influenza A NP 366-374 ASNENMETM). In addition, a fluorescence minus one (FMO) control without the tetramer or negative control antibody, as well as single antibody staining were prepared as flow cytometry control and compensation groups. Cells were washed twice with PBS and then centrifuged at 350 × *g* at 4 °C for 5 min. Finally, PBS + 4% paraformaldehyde (in-house) was added to the cells to fix them and allow removal from the BSL3 laboratory for flow cytometry acquisition with FACS Canto II (BD Bioscience) using the DIVA software.

### Spatial transcriptomics and gene expression analysis

Lung tissue sections (5-μm-thick formalin-fixed paraffin-embedded) were placed on Visium Spatial Gene Expression slides (10X Genomics) containing four capture areas each and processed according to manufacturer recommendations. In addition to the mouse transcriptome probes, we designed probes for the SARS-CoV-2 virus targeting ORF1ab, ORF3a, ORF10 and the genes encoding the structural proteins spike (S), envelope (E), membrane (M) and nucleocapsid (N). The custom SARS-CoV-2 probes are listed in Supplementary Table 4 and the final concentration for each primer in the probe hybridization mix was 1.2 nM. The cDNA libraries were loaded onto the NovaSeq 6000 system (Illumina) and sequenced with a minimum of 50,000 reads per covered spot. Reads contained in Illumina FASTQ files were aligned to a custom multispecies reference transcriptome generated with Space Ranger using the GRCm38 (v.mm10-2020-A_build, 10X Genomics) mouse and NC_045512.2 SARS-CoV-2 references. Downstream data analysis of the mouse samples was performed using SCANPY^39^. To compare host and viral gene expression levels across conditions, the counts were first normalized and then log transformed. To examine spatial correlations between total viral mRNA counts and host genes, pairwise Pearson’s correlation coefficients were calculated and compared across conditions. Cellular pathway activity scores for 13 different cellular pathways were calculated using PROGENy^40^.

### Statistics and reproducibility

Statistical analysis was performed using GraphPad Prism 9 (v.9.5.1). Unless noted otherwise, the results are expressed as mean ± s.e.m. Specific tests are indicated in the main text or the figure legends. The statistical tests used were sufficient to check for significant differences in parameters such as body weight and viral genome loads. Data collection was not performed blind to the conditions of the experiments, while sample analysis was blinded when possible (PCR analysis, pathological investigations). Data distribution was assumed to be normal, but this was not formally tested. For OTS-228 BA.5 and the OTS-228 XBB.1.5 challenge experiments, animals were excluded from comparisons, as directly mentioned in the respective figures, to avoid comparing different timepoints with different viral loads. For in vivo experiments, the sample size was calculated to be sufficient to achieve a power of 1−*ß* = 80%. LLM (openai.com) was used to improve readability and shorten the original text. Schematic overviews for animal experiments were created with BioRender.com.

### Reporting summary

Further information on research design is available in the Nature Portfolio Reporting Summary linked to this article.

### Supplementary information

Supplementary Information

Reporting Summary

Supplementary Data 1 Statistical source data for Supplementary Figs. 3 and 5.

### Source data

Source Data Figs. 1–6 and Extended Data Figs. 1–10 Statistical source data.

## Data Availability

All data are available in the main text or the supplementary materials. Project information and sequencing data are accessible with the BioProject ID PRJNA1002985. Source data are provided with this paper.
