# Supplementary material for: A safe, effective and adaptable live-attenuated SARS-CoV-2 vaccine to reduce disease and transmission using one-to-stop genome modifications
Source: Nat Microbiol. 2024 Jul 12;9(8):2099–112. doi: 10.1038/s41564-024-01755-1 (PMC11306094; doi:10.1038/s41564-024-01755-1)
Supplement: Supplementary file 1 — Supplementary Information [file 41564_2024_1755_MOESM1_ESM.pdf]

nature microbiology

Supplementary information

https://doi.org/10.1038/s41564-024-01755-1

A safe, effective and adaptable live-attenuated SARS-CoV-2 vaccine to reduce disease and transmission using one-to-stop genome modifications

In the format provided by the authors and unedited

Supplementary Table 1: *List of viruses used for the experiments.*

Abbreviation used in the manuscript	Name	Explanation	Accession Number	Experiment
**Challenge viruses**				
WT	BetaCoV/Wuhan/IVDC-HB-01/2019	wild-type SARS-CoV-2 – Lineage B	MT108784	Fig.2 l-p; Extended Data Fig. 4 l,m
WT^{D614G}	BetaCoV/Germany/BavPat1/2020	SARS-CoV-2 WT with D614G mutation – Lineage B.1	EPI_ISL_406862	VNT (hamster), Fig. 3 g-m, Fig. 4 b-c
Delta (B.1.617.2)	hCoV-19/Germany/BW-FR1407/2021	VOC Delta GK (B.1.617.2+AY.*)	EPI_ISL_2535433	Fig. 3 a-m; Extended Data Fig. 6 and 7
BA.2	SARS-CoV-2/human/NLD/EMC-BA2-1/2022	VOC Omicron BA.2	ON545852	Fig.2 q-v; Extended Data Fig. 4 o,p; VNT (hamster)
BA.5	hCoV-19/South Africa/CERI-KRISP-K040013/2022	VOC Omicron BA.5	EPI_ISL_12268493.2	Fig.4 k-r; Extended Data Fig. 10 a-c; VNT (hamster)
XBB.1.5	hCoV-19/Netherlands/NH-EMC-5667/2023	VOC Omicron XBB.1.5	EPI_ISL_16640568	Extended Data Fig. 10
**Modified viruses**				
OTS2		WT SARS-CoV-2 with OTS modifications in fragment 2		
OTS7		WT SARS-CoV-2 with OTS modifications in fragment 7		
OTS8		WT SARS-CoV-2 with OTS modifications in fragment 8		
OTS4-5		WT SARS-CoV-2 with OTS modifications in fragments 4 and 5		
OTS7-8		WT SARS-CoV-2 with OTS modifications in fragments 7 and 8		
OTS4-5-7-8		WT SARS-CoV-2 with OTS modifications in fragments 4,5,7and 8		
OTS-206	OTS4-5-7-8.$NSP1^{K164A, H165A}$.delORF6-8	OTS in fragments 4-5, 7-8; point mutations in NSP1(K164A, H165A), deletion of ORF6 to ORF8		
OTS-228	OTS4-5-7-8.$NSP1^{K164A, H165A}$.delORF6-8.delCS	OTS in fragments 4-5, 7-8; point mutations in NSP1(K164A, H165A), deletion of ORF6 to ORF8, deletion of 24 nucleotides in furin cleavage site (23598-23622)		
delORF6-8		WT SARS-CoV-2, deletion of ORF6 to ORF8		
nsp1		WT SARS-CoV-2, point mutations in NSP1(K164A, H165A)		
SARS-CoV-$2^{\Delta CS}$		WT SARS-CoV-2, deletion of 24 nucleotides in furin cleavage site (23598-23622)		

Supplementary Table 2: *Primers.*

OTS primer and modifications introduced into the SARS-CoV-2 genome.

OTS primers				
Fragment	Primer name	Primer sequence (5' - 3')	Primer length	Amplified region (bp)
Fg 2 nsp1_dm1 (K164A and H165A)	WU-5-F	GTCTTATCAGAGGCACGTCAAC	22	405
	WU-124-R	TTCACGGGTAACACCACTGCTAGCAGCAGTGTTCCAGTTTTCTTGAAA	48	
	WU-135-F	TTTTCAAGAAAACTGGAACACTGCTGCTAGCAGTGGTGTTACCCGTGAA	49	2588
	WU-6-R	CTGGTGTAAGTTCCATCTCTAATTG	25	
Fg 11 Mutant delORF6-8	WU-23-F	GATGGCAACTAGCACTCTCC	20	1622
	CoV2mutdel6-8-R	TTAGTTTGTTCGTTTAGATGAAATCTTACTGTACAAGCAAAGCAATATTGTC	52	
	CoV2mutdel6-8-F	CAATATTGCTTTGCTTGTACAGTAAGATTTCATCTAAACGAACAAACTAAAATGTC	56	557
	WU-24-R	TTTGGCAATGTTGTTCCTTGAGG	23	
Fg 9&10 Bristol del	WU-19-F	GGAGTCACATTAATTGGAGAAGC	23	3488
	CoV2-Sp-bristol del-R	TAGGCAATGATGGATTGACTAGCTATAGTCTGAGTCTGATAACTAGCGCA	50	
	CoV2-Sp-bristol del-F	TGCGCTAGTTATCAGACTCAGACTATAGCTAGTCAATCCATCATTGCCTA	50	2319
	WU-22-R	TCATGTTCAGAAATAGGACTTGTTG	25	

RTqPCR primer for the detection of viral genome.

RTPCR primers				
Gene	Primer name	Primer sequence (5' - 3')	Primer length	Amplified region (bp)
At IVI:				
E (forward)	PWhSF-E-F21	ACAGGTACGTTAATAGTTAATAGCGTACTTCT	32	116
E (reverse)	PWhSF-R-22	ACAATATTGCAGCAGTACGCACA	23	
E (probe)	PWhSF-E-P23mgb	ATCCTTACTGCGCTTCGA	18	
At FLI:				
RdRp gene / nCoV_IP4	Reference: National Reference Center for Respiratory Viruses, Institut Pasteur, Paris.			
nCoV_IP4-14059Fw		GGTAACTGGTATGATTTCG	19	107
nCoV_IP4-14146Rv		CTGGTCAAGGTTAATATAGG	20	
nCoV_IP4-14084Probe(+)		TCATACAAACCACGCCAGG [5']Fam [3']BHQ-1	19	

__Supplementary Table 3__: Modifications introduced into the SARS-CoV-2 genome.

OTS changes introduced into SARS-CoV-2 genome						
OTS	Length	Amino Acid Change	CDS	Change	Codon Change	CDS Codon Number
Fg 2 - OTS	1	-	polyprotein 1ab CDS	C -> T	CTG -> TTG	88
Fg 2 - OTS	2	-	polyprotein 1ab CDS	CTC -> TTG	CTC -> TTG	92
Fg 2 - OTS	3	-	polyprotein 1ab CDS	AGT -> TCA	AGT -> TCA	100
Fg 2 - OTS	2	-	polyprotein 1ab CDS	CTT -> TTA	CTT -> TTA	104
Fg 2 - OTS	2	-	polyprotein 1ab CDS	CTT -> TTA	CTT -> TTA	107
Fg 2 - OTS	2	-	polyprotein 1ab CDS	CTT -> TTA	CTT -> TTA	122
Fg 2 - OTS	2	-	polyprotein 1ab CDS	CTT -> TTA	CTT -> TTA	123
Fg 2 - OTS	1	-	polyprotein 1ab CDS	C -> T	CTA -> TTA	140
Fg 2 - OTS	2	-	polyprotein 1ab CDS	CTT -> TTA	CTT -> TTA	149
Fg 2 - OTS	3	-	polyprotein 1ab CDS	AGC -> TCG	AGC -> TCG	166
Fg 2 - OTS	3	-	polyprotein 1ab CDS	AGT -> TCA	AGT -> TCA	167
Fg 2 - OTS	2	-	polyprotein 1ab CDS	CTC -> TTG	CTC -> TTG	173
Fg 2 - OTS	2	-	polyprotein 1ab CDS	CTT -> TTA	CTT -> TTA	177
Fg 2 - OTS	2	-	polyprotein 1ab CDS	CTT -> TTA	CTT -> TTA	198
Fg 2 - OTS	2	-	polyprotein 1ab CDS	CTT -> TTA	CTT -> TTA	204
Fg 2 - OTS	1	-	polyprotein 1ab CDS	C -> T	CTA -> TTA	205
Fg 2 - OTS	1	-	polyprotein 1ab CDS	C -> T	CTG -> TTG	219
Fg 2 - OTS	1	-	polyprotein 1ab CDS	T -> A	TCT -> TCA	245
Fg 2 - OTS	3	-	polyprotein 1ab CDS	AGC -> TCG	AGC -> TCG	248
Fg 2 - OTS	1	-	polyprotein 1ab CDS	C -> G	TCC -> TCG	279
Fg 2 - OTS	2	-	polyprotein 1ab CDS	CTT -> TTA	CTT -> TTA	293
Fg 2 - OTS	1	-	polyprotein 1ab CDS	T -> A	TCT -> TCA	302
Fg 2 - OTS	2	-	polyprotein 1ab CDS	CTC -> TTG	CTC -> TTG	320
Fg 2 - OTS	3	-	polyprotein 1ab CDS	AGT -> TCA	AGT -> TCA	383
Fg 2 - OTS	1	-	polyprotein 1ab CDS	T -> A	TCT -> TCA	391
Fg 2 - OTS	2	-	polyprotein 1ab CDS	CTT -> TTA	CTT -> TTA	397
Fg 2 - OTS	1	-	polyprotein 1ab CDS	T -> A	TCT -> TCA	412
Fg 2 - OTS	3	-	polyprotein 1ab CDS	AGC -> TCG	AGC -> TCG	428
Fg 2 - OTS	1	-	polyprotein 1ab CDS	C -> G	TCC -> TCG	443
Fg 2 - OTS	2	-	polyprotein 1ab CDS	CTT -> TTA	CTT -> TTA	446
Fg 2 - OTS	2	-	polyprotein 1ab CDS	CTT -> TTA	CTT -> TTA	450
Fg 2 - OTS	2	-	polyprotein 1ab CDS	CTT -> TTA	CTT -> TTA	451
Fg 2 - OTS	2	-	polyprotein 1ab CDS	CTC -> TTG	CTC -> TTG	454
Fg 2 - OTS	2	-	polyprotein 1ab CDS	CTT -> TTA	CTT -> TTA	469
Fg 2 - OTS	1	-	polyprotein 1ab CDS	T -> A	TCT -> TCA	479
Fg 2 - OTS	1	-	polyprotein 1ab CDS	T -> A	TCT -> TCA	481
Fg 2 - OTS	1	-	polyprotein 1ab CDS	C -> G	TCC -> TCG	483
Fg 2 - OTS	3	-	polyprotein 1ab CDS	AGT -> TCA	AGT -> TCA	485
Fg 2 - OTS	1	-	polyprotein 1ab CDS	C -> G	TCC -> TCG	505
Fg 2 - OTS	1	-	polyprotein 1ab CDS	C -> T	CTG -> TTG	530
Fg 2 - OTS	3	-	polyprotein 1ab CDS	AGT -> TCA	AGT -> TCA	531
Fg 2 - OTS	2	-	polyprotein 1ab CDS	CTT -> TTG	CTT -> TTG	533
Fg 2 - OTS	1	-	polyprotein 1ab CDS	C -> G	TCC -> TCG	549
Fg 2 - OTS	2	-	polyprotein 1ab CDS	CTT -> TTA	CTT -> TTA	552
Fg 2 - OTS	1	-	polyprotein 1ab CDS	T -> A	TCT -> TCA	558
Fg 2 - OTS	1	-	polyprotein 1ab CDS	C -> T	CTA -> TTA	570
Fg 2 - OTS	1	-	polyprotein 1ab CDS	C -> T	CTG -> TTG	578
Fg 2 - OTS	2	-	polyprotein 1ab CDS	CTC -> TTG	CTC -> TTG	580
Fg 2 - OTS	1	-	polyprotein 1ab CDS	T -> A	TCT -> TCA	588
Fg 2 - OTS	1	-	polyprotein 1ab CDS	C -> T	CTA -> TTA	595
Fg 2 - OTS	1	-	polyprotein 1ab CDS	C -> T	CTA -> TTA	613
Fg 2 - OTS	2	-	polyprotein 1ab CDS	CTC -> TTG	CTC -> TTG	624
Fg 2 - OTS	2	-	polyprotein 1ab CDS	CTT -> TTA	CTT -> TTA	628
Fg 2 - OTS	2	-	polyprotein 1ab CDS	CTT -> TTA	CTT -> TTA	631
Fg 2 - OTS	2	-	polyprotein 1ab CDS	CTT -> TTA	CTT -> TTA	642
Fg 2 - OTS	3	-	polyprotein 1ab CDS	AGT -> TCA	AGT -> TCA	674
Fg 2 - OTS	2	-	polyprotein 1ab CDS	CTT -> TTA	CTT -> TTA	681
Fg 2 - OTS	1	-	polyprotein 1ab CDS	T -> A	TCT -> TCA	692
Fg 2 - OTS	2	-	polyprotein 1ab CDS	CTT -> TTA	CTT -> TTA	700
Fg 2 - OTS	1	-	polyprotein 1ab CDS	C -> G	TCC -> TCG	723
Fg 2 - OTS	1	-	polyprotein 1ab CDS	C -> T	CTA -> TTA	729
Fg 2 - OTS	2	-	polyprotein 1ab CDS	CTC -> TTG	CTC -> TTG	730
Fg 2 - OTS	1	-	polyprotein 1ab CDS	C -> T	CTA -> TTA	733
Fg 2 - OTS	2	-	polyprotein 1ab CDS	CTT -> TTA	CTT -> TTA	747
Fg 2 - OTS	3	-	polyprotein 1ab CDS	AGT -> TCA	AGT -> TCA	771
Fg 2 - OTS	2	-	polyprotein 1ab CDS	CTT -> TTA	CTT -> TTA	788
Fg 2 - OTS	2	-	polyprotein 1ab CDS	CTC -> TTG	CTC -> TTG	791
Fg 2 - OTS	2	-	polyprotein 1ab CDS	CTC -> TTG	CTC -> TTG	815
Fg 2 - OTS	3	-	polyprotein 1ab CDS	AGT -> TCA	AGT -> TCA	838
Fg 2 - OTS	2	-	polyprotein 1ab CDS	CTT -> TTA	CTT -> TTA	845
Fg 2 - OTS	2	-	polyprotein 1ab CDS	CTT -> TTA	CTT -> TTA	853
Fg 2 - OTS	1	-	polyprotein 1ab CDS	T -> A	TCT -> TCA	858
Fg 2 - OTS	2	-	polyprotein 1ab CDS	CTC -> TTG	CTC -> TTG	864
Fg 2 - OTS	1	-	polyprotein 1ab CDS	T -> A	TCT -> TCA	887
Fg 2 - OTS	1	-	polyprotein 1ab CDS	C -> T	CTG -> TTG	893
Fg 2 - OTS	3	-	polyprotein 1ab CDS	AGT -> TCA	AGT -> TCA	901
Fg 2 – OTS	1	-	polyprotein 1ab CDS	T -> A	TCT -> TCA	911
Total Fg2 - OTS	136			Total codon changes Fg 2 = 77		

Supplementary Table 3 (continued): *Modifications introduced into the SARS-CoV-2 genome.*

Fg 4 - OTS	1-	polyprotein 1ab CDS	C -> T	CTG -> TTG	2028
Fg 4 - OTS	2-	polyprotein 1ab CDS	CTT -> TTA	CTT -> TTA	2039
Fg 4 - OTS	1-	polyprotein 1ab CDS	C -> T	CTA -> TTA	2044
Fg 4 - OTS	1-	polyprotein 1ab CDS	T -> A	TCT -> TCA	2048
Fg 4 - OTS	2-	polyprotein 1ab CDS	CTT -> TTA	CTT -> TTA	2062
Fg 4 - OTS	2-	polyprotein 1ab CDS	CTT -> TTA	CTT -> TTA	2077
Fg 4 - OTS	3-	polyprotein 1ab CDS	AGT -> TCA	AGT -> TCA	2083
Fg 4 - OTS	1-	polyprotein 1ab CDS	C -> T	CTA -> TTA	2095
Fg 4 - OTS	1-	polyprotein 1ab CDS	T -> A	TCT -> TCA	2103
Fg 4 - OTS	3-	polyprotein 1ab CDS	AGT -> TCA	AGT -> TCA	2104
Fg 4 - OTS	1-	polyprotein 1ab CDS	T -> A	TCT -> TCA	2114
Fg 4 - OTS	2-	polyprotein 1ab CDS	CTT -> TTA	CTT -> TTA	2122
Fg 4 - OTS	3-	polyprotein 1ab CDS	AGT -> TCA	AGT -> TCA	2132
Fg 4 - OTS	2-	polyprotein 1ab CDS	CTT -> TTA	CTT -> TTA	2146
Fg 4 - OTS	3-	polyprotein 1ab CDS	AGT -> TCA	AGT -> TCA	2151
Fg 4 - OTS	1-	polyprotein 1ab CDS	C -> T	CTA -> TTA	2177
Fg 4 - OTS	3-	polyprotein 1ab CDS	AGT -> TCA	AGT -> TCA	2185
Fg 4 - OTS	1-	polyprotein 1ab CDS	T -> A	TCT -> TCA	2188
Fg 4 - OTS	1-	polyprotein 1ab CDS	T -> A	TCT -> TCA	2193
Fg 4 - OTS	3-	polyprotein 1ab CDS	AGT -> TCA	AGT -> TCA	2205
Fg 4 - OTS	1-	polyprotein 1ab CDS	C -> T	CTA -> TTA	2211
Fg 4 - OTS	1-	polyprotein 1ab CDS	T -> A	TCT -> TCA	2224
Fg 4 - OTS	1-	polyprotein 1ab CDS	C -> T	CTG -> TTG	2226
Fg 4 - OTS	1-	polyprotein 1ab CDS	C -> T	CTA -> TTA	2235
Fg 4 - OTS	3-	polyprotein 1ab CDS	AGT -> TCA	AGT -> TCA	2237
Fg 4 - OTS	1-	polyprotein 1ab CDS	C -> T	CTA -> TTA	2240
Fg 4 - OTS	1-	polyprotein 1ab CDS	T -> A	TCT -> TCA	2242
Fg 4 - OTS	1-	polyprotein 1ab CDS	T -> A	TCT -> TCA	2255
Fg 4 - OTS	1-	polyprotein 1ab CDS	T -> A	TCT -> TCA	2261
Fg 4 - OTS	1-	polyprotein 1ab CDS	T -> A	TCT -> TCA	2273
Fg 4 - OTS	1-	polyprotein 1ab CDS	T -> A	TCT -> TCA	2285
Fg 4 - OTS	3-	polyprotein 1ab CDS	AGT -> TCA	AGT -> TCA	2289
Fg 4 - OTS	2-	polyprotein 1ab CDS	CTT -> TTA	CTT -> TTA	2292
Fg 4 - OTS	3-	polyprotein 1ab CDS	AGT -> TCA	AGT -> TCA	2293
Fg 4 - OTS	1-	polyprotein 1ab CDS	T -> A	TCT -> TCA	2297
Fg 4 - OTS	1-	polyprotein 1ab CDS	T -> A	TCT -> TCA	2303
Fg 4 - OTS	1-	polyprotein 1ab CDS	T -> A	TCT -> TCA	2313
Fg 4 - OTS	2-	polyprotein 1ab CDS	CTT -> TTA	CTT -> TTA	2333
Fg 4 - OTS	2-	polyprotein 1ab CDS	CTT -> TTA	CTT -> TTA	2341
Fg 4 - OTS	3-	polyprotein 1ab CDS	AGC -> TCG	AGC -> TCG	2352
Fg 4 - OTS	3-	polyprotein 1ab CDS	AGT -> TCA	AGT -> TCA	2360
Fg 4 - OTS	1-	polyprotein 1ab CDS	T -> A	TCT -> TCA	2362
Fg 4 - OTS	2-	polyprotein 1ab CDS	CTT -> TTA	CTT -> TTA	2364
Fg 4 - OTS	2-	polyprotein 1ab CDS	CTT -> TTA	CTT -> TTA	2371
Fg 4 - OTS	3-	polyprotein 1ab CDS	AGT -> TCA	AGT -> TCA	2396
Fg 4 - OTS	1-	polyprotein 1ab CDS	C -> G	TCC -> TCG	2433
Fg 4 - OTS	1-	polyprotein 1ab CDS	C -> T	CTA -> TTA	2447
Fg 4 - OTS	3-	polyprotein 1ab CDS	AGT -> TCA	AGT -> TCA	2462
Fg 4 - OTS	3-	polyprotein 1ab CDS	AGT -> TCA	AGT -> TCA	2466
Fg 4 - OTS	1-	polyprotein 1ab CDS	C -> T	CTA -> TTA	2475
Fg 4 - OTS	1-	polyprotein 1ab CDS	T -> A	TCT -> TCA	2487
Fg 4 - OTS	1-	polyprotein 1ab CDS	T -> A	TCT -> TCA	2488
Fg 4 - OTS	3-	polyprotein 1ab CDS	AGT -> TCA	AGT -> TCA	2493
Fg 4 - OTS	1-	polyprotein 1ab CDS	C -> G	TCC -> TCG	2500
Fg 4 - OTS	2-	polyprotein 1ab CDS	CTT -> TTA	CTT -> TTA	2503
Fg 4 - OTS	1-	polyprotein 1ab CDS	T -> A	TCT -> TCA	2517
Fg 4 - OTS	2-	polyprotein 1ab CDS	CTC -> TTG	CTC -> TTG	2518
Fg 4 - OTS	1-	polyprotein 1ab CDS	T -> A	TCT -> TCA	2519
Fg 4 - OTS	1-	polyprotein 1ab CDS	C -> T	CTG -> TTG	2527
Fg 4 - OTS	1-	polyprotein 1ab CDS	T -> A	TCT -> TCA	2553
Fg 4 - OTS	1-	polyprotein 1ab CDS	T -> A	TCT -> TCA	2558
Fg 4 - OTS	2-	polyprotein 1ab CDS	CTT -> TTA	CTT -> TTA	2564
Fg 4 - OTS	1-	polyprotein 1ab CDS	C -> T	CTG -> TTG	2570
Fg 4 - OTS	1-	polyprotein 1ab CDS	C -> T	CTA -> TTA	2572
Fg 4 - OTS	1-	polyprotein 1ab CDS	T -> A	TCT -> TCA	2578
Fg 4 - OTS	3-	polyprotein 1ab CDS	AGT -> TCA	AGT -> TCA	2583
Fg 4 - OTS	2-	polyprotein 1ab CDS	CTC -> TTG	CTC -> TTG	2609
Fg 4 - OTS	1-	polyprotein 1ab CDS	C -> T	CTA -> TTA	2612
Fg 4 - OTS	2-	polyprotein 1ab CDS	CTT -> TTA	CTT -> TTA	2620
Fg 4 - OTS	1-	polyprotein 1ab CDS	C -> G	TCC -> TCG	2625
Fg 4 - OTS	1-	polyprotein 1ab CDS	T -> A	TCT -> TCA	2631
Fg 4 - OTS	2-	polyprotein 1ab CDS	CTT -> TTA	CTT -> TTA	2655
Fg 4 - OTS	1-	polyprotein 1ab CDS	T -> A	TCT -> TCA	2661
Fg 4 - OTS	3-	polyprotein 1ab CDS	AGT -> TCA	AGT -> TCA	2669
Fg 4 - OTS	2-	polyprotein 1ab CDS	CTC -> TTG	CTC -> TTG	2675
Fg 4 - OTS	2-	polyprotein 1ab CDS	CTT -> TTA	CTT -> TTA	2688
Fg 4 - OTS	3-	polyprotein 1ab CDS	AGT -> TCA	AGT -> TCA	2695
Fg 4 - OTS	3-	polyprotein 1ab CDS	AGT -> TCA	AGT -> TCA	2706
Fg 4 - OTS	1-	polyprotein 1ab CDS	T -> A	TCT -> TCA	2722
Fg 4 - OTS	1-	polyprotein 1ab CDS	C -> T	CTA -> TTA	2725
Fg 4 - OTS	3-	polyprotein 1ab CDS	AGT -> TCA	AGT -> TCA	2731
Fg 4 - OTS	2-	polyprotein 1ab CDS	CTT -> TTA	CTT -> TTA	2760
Fg 4 - OTS	2-	polyprotein 1ab CDS	CTT -> TTA	CTT -> TTA	2778
Fg 4 - OTS	2-	polyprotein 1ab CDS	CTT -> TTA	CTT -> TTA	2781
Fg 4 - OTS	1-	polyprotein 1ab CDS	T -> A	TCT -> TCA	2797
Fg 4 – OTS	3-	polyprotein 1ab CDS	AGT -> TCA	AGT -> TCA	2804
Total Fg 4 - OTS	149			Total codon changes Fg 4 = 86	

Supplementary Table 3 (continued): *Modifications introduced into the SARS-CoV-2 genome.*

Fg 5 - OTS	1-	polyprotein 1ab CDS	T -> A	TCT -> TCA	2926
Fg 5 - OTS	1-	polyprotein 1ab CDS	C -> T	CTA -> TTA	2939
Fg 5 - OTS	1-	polyprotein 1ab CDS	T -> A	TCT -> TCA	2942
Fg 5 - OTS	3-	polyprotein 1ab CDS	AGT -> TCA	AGT -> TCA	2947
Fg 5 - OTS	2-	polyprotein 1ab CDS	CTC -> TTG	CTC -> TTG	2956
Fg 5 - OTS	1-	polyprotein 1ab CDS	T -> A	TCT -> TCA	2960
Fg 5 - OTS	2-	polyprotein 1ab CDS	CTT -> TTA	CTT -> TTA	2969
Fg 5 - OTS	1-	polyprotein 1ab CDS	T -> A	TCT -> TCA	2972
Fg 5 - OTS	1-	polyprotein 1ab CDS	T -> A	TCT -> TCA	2981
Fg 5 - OTS	1-	polyprotein 1ab CDS	T -> A	TCT -> TCA	2999
Fg 5 - OTS	3-	polyprotein 1ab CDS	AGT -> TCA	AGT -> TCA	3001
Fg 5 - OTS	2-	polyprotein 1ab CDS	CTT -> TTA	CTT -> TTA	3006
Fg 5 - OTS	1-	polyprotein 1ab CDS	T -> A	TCT -> TCA	3013
Fg 5 - OTS	2-	polyprotein 1ab CDS	CTT -> TTA	CTT -> TTA	3027
Fg 5 - OTS	1-	polyprotein 1ab CDS	C -> T	CTA -> TTA	3034
Fg 5 - OTS	1-	polyprotein 1ab CDS	T -> A	TCT -> TCA	3046
Fg 5 - OTS	2-	polyprotein 1ab CDS	CTT -> TTA	CTT -> TTA	3060
Fg 5 - OTS	3-	polyprotein 1ab CDS	AGT -> TCA	AGT -> TCA	3075
Fg 5 - OTS	1-	polyprotein 1ab CDS	C -> T	CTA -> TTA	3084
Fg 5 - OTS	2-	polyprotein 1ab CDS	CTT -> TTA	CTT -> TTA	3086
Fg 5 - OTS	2-	polyprotein 1ab CDS	CTC -> TTG	CTC -> TTG	3092
Fg 5 - OTS	1-	polyprotein 1ab CDS	T -> A	TCT -> TCA	3106
Fg 5 - OTS	2-	polyprotein 1ab CDS	CTT -> TTA	CTT -> TTA	3116
Fg 5 - OTS	1-	polyprotein 1ab CDS	T -> A	TCT -> TCA	3121
Fg 5 - OTS	1-	polyprotein 1ab CDS	C -> G	TCC -> TCG	3149
Fg 5 - OTS	3-	polyprotein 1ab CDS	AGT -> TCA	AGT -> TCA	3158
Fg 5 - OTS	1-	polyprotein 1ab CDS	C -> T	CTA -> TTA	3161
Fg 5 - OTS	1-	polyprotein 1ab CDS	C -> G	TCC -> TCG	3171
Fg 5 - OTS	3-	polyprotein 1ab CDS	AGT -> TCA	AGT -> TCA	3173
Fg 5 - OTS	1-	polyprotein 1ab CDS	C -> T	CTG -> TTG	3180
Fg 5 - OTS	1-	polyprotein 1ab CDS	C -> T	CTA -> TTA	3191
Fg 5 - OTS	3-	polyprotein 1ab CDS	AGT -> TCA	AGT -> TCA	3195
Fg 5 - OTS	1-	polyprotein 1ab CDS	C -> T	CTA -> TTA	3198
Fg 5 - OTS	2-	polyprotein 1ab CDS	CTT -> TTA	CTT -> TTA	3201
Fg 5 - OTS	2-	polyprotein 1ab CDS	CTT -> TTA	CTT -> TTA	3210
Fg 5 - OTS	3-	polyprotein 1ab CDS	AGT -> TCA	AGT -> TCA	3218
Fg 5 - OTS	3-	polyprotein 1ab CDS	AGC -> TCG	AGC -> TCG	3225
Fg 5 - OTS	2-	polyprotein 1ab CDS	CTC -> TTG	CTC -> TTG	3234
Fg 5 - OTS	2-	polyprotein 1ab CDS	CTC -> TTG	CTC -> TTG	3238
Fg 5 - OTS	3-	polyprotein 1ab CDS	AGT -> TCA	AGT -> TCA	3242
Fg 5 - OTS	1-	polyprotein 1ab CDS	T -> A	TCT -> TCA	3246
Fg 5 - OTS	2-	polyprotein 1ab CDS	CTT -> TTA	CTT -> TTA	3249
Fg 5 - OTS	1-	polyprotein 1ab CDS	T -> A	TCT -> TCA	3256
Fg 5 - OTS	3-	polyprotein 1ab CDS	AGT -> TCA	AGT -> TCA	3264
Fg 5 - OTS	1-	polyprotein 1ab CDS	T -> A	TCT -> TCA	3273
Fg 5 - OTS	2-	polyprotein 1ab CDS	CTT -> TTA	CTT -> TTA	3290
Fg 5 - OTS	2-	polyprotein 1ab CDS	CTT -> TTA	CTT -> TTA	3293
Fg 5 - OTS	2-	polyprotein 1ab CDS	CTT -> TTA	CTT -> TTA	3295
Fg 5 - OTS	1-	polyprotein 1ab CDS	T -> A	TCT -> TCA	3309
Fg 5 - OTS	2-	polyprotein 1ab CDS	CTT -> TTA	CTT -> TTA	3313
Fg 5 - OTS	2-	polyprotein 1ab CDS	CTC -> TTG	CTC -> TTG	3321
Fg 5 - OTS	1-	polyprotein 1ab CDS	T -> A	TCT -> TCA	3325
Fg 5 - OTS	2-	polyprotein 1ab CDS	CTC -> TTG	CTC -> TTG	3338
Fg 5 - OTS	1-	polyprotein 1ab CDS	T -> A	TCT -> TCA	3344
Fg 5 - OTS	2-	polyprotein 1ab CDS	CTT -> TTA	CTT -> TTA	3350
Fg 5 - OTS	2-	polyprotein 1ab CDS	CTT -> TTA	CTT -> TTA	3352
Fg 5 - OTS	1-	polyprotein 1ab CDS	T -> A	TCT -> TCA	3386
Fg 5 - OTS	2-	polyprotein 1ab CDS	CTT -> TTA	CTT -> TTA	3404
Fg 5 - OTS	3-	polyprotein 1ab CDS	AGT -> TCA	AGT -> TCA	3410
Fg 5 - OTS	1-	polyprotein 1ab CDS	T -> A	TCT -> TCA	3421
Fg 5 - OTS	2-	polyprotein 1ab CDS	CTC -> TTG	CTC -> TTG	3483
Fg 5 - OTS	2-	polyprotein 1ab CDS	CTT -> TTA	CTT -> TTA	3490
Fg 5 - OTS	2-	polyprotein 1ab CDS	CTT -> TTA	CTT -> TTA	3495
Fg 5 - OTS	1-	polyprotein 1ab CDS	C -> T	CTA -> TTA	3505
Fg 5 - OTS	1-	polyprotein 1ab CDS	C -> T	CTA -> TTA	3513
Fg 5 - OTS	2-	polyprotein 1ab CDS	CTT -> TTA	CTT -> TTA	3516
Fg 5 - OTS	1-	polyprotein 1ab CDS	C -> T	CTG -> TTG	3535
Fg 5 - OTS	3-	polyprotein 1ab CDS	AGT -> TCA	AGT -> TCA	3547
Fg 5 - OTS	3-	polyprotein 1ab CDS	AGT -> TCA	AGT -> TCA	3570
Fg 5 - OTS	2-	polyprotein 1ab CDS	CTC -> TTG	CTC -> TTG	3585
Fg 5 - OTS	2-	polyprotein 1ab CDS	CTT -> TTA	CTT -> TTA	3591
Fg 5 - OTS	3-	polyprotein 1ab CDS	AGT -> TCA	AGT -> TCA	3597
Fg 5 - OTS	1-	polyprotein 1ab CDS	T -> A	TCT -> TCA	3601
Fg 5 - OTS	1-	polyprotein 1ab CDS	T -> A	TCT -> TCA	3622
Fg 5 - OTS	2-	polyprotein 1ab CDS	CTC -> TTG	CTC -> TTG	3636
Fg 5 - OTS	1-	polyprotein 1ab CDS	T -> A	TCT -> TCA	3643
Fg 5 - OTS	2-	polyprotein 1ab CDS	CTT -> TTA	CTT -> TTA	3644
Fg 5 - OTS	3-	polyprotein 1ab CDS	AGT -> TCA	AGT -> TCA	3658
Fg 5 - OTS	3-	polyprotein 1ab CDS	AGT -> TCA	AGT -> TCA	3673
Fg 5 - OTS	1-	polyprotein 1ab CDS	T -> A	TCT -> TCA	3675
Fg 5 - OTS	1-	polyprotein 1ab CDS	C -> T	CTA -> TTA	3679
Fg 5 - OTS	1-	polyprotein 1ab CDS	C -> T	CTA -> TTA	3692
Fg 5 - OTS	2-	polyprotein 1ab CDS	CTT -> TTA	CTT -> TTA	3694
Fg 5 - OTS	2-	polyprotein 1ab CDS	CTT -> TTA	CTT -> TTA	3711
Fg 5 - OTS	2-	polyprotein 1ab CDS	CTC -> TTG	CTC -> TTG	3717
Fg 5 - OTS	1-	polyprotein 1ab CDS	C -> G	TCC -> TCG	3732
Fg 5 - OTS	2-	polyprotein 1ab CDS	CTT -> TTA	CTT -> TTA	3736
Fg 5 - OTS	1-	polyprotein 1ab CDS	T -> A	TCT -> TCA	3739
Fg 5 - OTS	1-	polyprotein 1ab CDS	T -> A	TCT -> TCA	3742
Fg 5 - OTS	2-	polyprotein 1ab CDS	CTT -> TTA	CTT -> TTA	3776
Fg 5 - OTS	1-	polyprotein 1ab CDS	C -> T	CTA -> TTA	3781
Fg 5 – OTS	2-	polyprotein 1ab CDS	CTC -> TTG	CTC -> TTG	3796
Total Fg 5 - OTS	160-			Total codon changes Fg 5 = 92	

Supplementary Table 3 (continued): Modifications introduced into the SARS-CoV-2 genome.

Fg 7 - OTS	2 -	polyprotein 1ab CDS	CTT -> TTA	CTT -> TTA	4793
Fg 7 - OTS	1 -	polyprotein 1ab CDS	T -> A	TCT -> TCA	4817
Fg 7 - OTS	3 -	polyprotein 1ab CDS	AGT -> TCA	AGT -> TCA	4825
Fg 7 - OTS	1 -	polyprotein 1ab CDS	T -> A	TCT -> TCA	4826
Fg 7 - OTS	3 -	polyprotein 1ab CDS	AGC -> TCG	AGC -> TCG	4843
Fg 7 - OTS	1 -	polyprotein 1ab CDS	C -> T	CTA -> TTA	4852
Fg 7 - OTS	1 -	polyprotein 1ab CDS	C -> T	CTA -> TTA	4861
Fg 7 - OTS	1 -	polyprotein 1ab CDS	C -> T	CTA -> TTA	4862
Fg 7 - OTS	1 -	polyprotein 1ab CDS	C -> T	CTA -> TTA	4890
Fg 7 - OTS	2 -	polyprotein 1ab CDS	CTT -> TTA	CTT -> TTA	4906
Fg 7 - OTS	3 -	polyprotein 1ab CDS	AGT -> TCA	AGT -> TCA	4912
Fg 7 - OTS	2 -	polyprotein 1ab CDS	CTT -> TTA	CTT -> TTA	4919
Fg 7 - OTS	2 -	polyprotein 1ab CDS	CTT -> TTA	CTT -> TTA	4936
Fg 7 - OTS	3 -	polyprotein 1ab CDS	AGT -> TCA	AGT -> TCA	4941
Fg 7 - OTS	1 -	polyprotein 1ab CDS	T -> A	TCT -> TCA	4953
Fg 7 - OTS	3 -	polyprotein 1ab CDS	AGT -> TCA	AGT -> TCA	4956
Fg 7 - OTS	3 -	polyprotein 1ab CDS	AGC -> TCG	AGC -> TCG	4984
Fg 7 - OTS	3 -	polyprotein 1ab CDS	AGT -> TCA	AGT -> TCA	4999
Fg 7 - OTS	2 -	polyprotein 1ab CDS	CTT -> TTA	CTT -> TTA	5006
Fg 7 - OTS	2 -	polyprotein 1ab CDS	CTT -> TTA	CTT -> TTA	5022
Fg 7 - OTS	2 -	polyprotein 1ab CDS	CTT -> TTA	CTT -> TTA	5028
Fg 7 - OTS	2 -	polyprotein 1ab CDS	CTT -> TTA	CTT -> TTA	5030
Fg 7 - OTS	3 -	polyprotein 1ab CDS	AGC -> TCG	AGC -> TCG	5039
Fg 7 - OTS	3 -	polyprotein 1ab CDS	AGT -> TCA	AGT -> TCA	5056
Fg 7 - OTS	1 -	polyprotein 1ab CDS	C -> T	CTA -> TTA	5065
Fg 7 - OTS	3 -	polyprotein 1ab CDS	AGT -> TCA	AGT -> TCA	5084
Fg 7 - OTS	2 -	polyprotein 1ab CDS	CTT -> TTA	CTT -> TTA	5099
Fg 7 - OTS	1 -	polyprotein 1ab CDS	T -> A	TCT -> TCA	5101
Fg 7 - OTS	2 -	polyprotein 1ab CDS	CTT -> TTA	CTT -> TTA	5119
Fg 7 - OTS	2 -	polyprotein 1ab CDS	CTC -> TTG	CTC -> TTG	5123
Fg 7 - OTS	2 -	polyprotein 1ab CDS	CTC -> TTG	CTC -> TTG	5150
Fg 7 - OTS	1 -	polyprotein 1ab CDS	T -> A	TCT -> TCA	5151
Fg 7 - OTS	3 -	polyprotein 1ab CDS	AGC -> TCG	AGC -> TCG	5160
Fg 7 - OTS	1 -	polyprotein 1ab CDS	T -> A	TCT -> TCA	5164
Fg 7 - OTS	1 -	polyprotein 1ab CDS	C -> T	CTA -> TTA	5167
Fg 7 - OTS	3 -	polyprotein 1ab CDS	AGC -> TCG	AGC -> TCG	5170
Fg 7 - OTS	2 -	polyprotein 1ab CDS	CTT -> TTA	CTT -> TTA	5178
Fg 7 - OTS	1 -	polyprotein 1ab CDS	T -> A	TCT -> TCA	5187
Fg 7 - OTS	2 -	polyprotein 1ab CDS	CTT -> TTA	CTT -> TTA	5197
Fg 7 - OTS	1 -	polyprotein 1ab CDS	T -> A	TCT -> TCA	5206
Fg 7 - OTS	1 -	polyprotein 1ab CDS	C -> T	CTA -> TTA	5211
Fg 7 - OTS	2 -	polyprotein 1ab CDS	CTT -> TTA	CTT -> TTA	5221
Fg 7 - OTS	1 -	polyprotein 1ab CDS	C -> T	CTA -> TTA	5230
Fg 7 - OTS	2 -	polyprotein 1ab CDS	CTT -> TTA	CTT -> TTA	5246
Fg 7 - OTS	1 -	polyprotein 1ab CDS	T -> A	TCT -> TCA	5253
Fg 7 - OTS	2 -	polyprotein 1ab CDS	CTT -> TTA	CTT -> TTA	5261
Fg 7 - OTS	1 -	polyprotein 1ab CDS	C -> T	CTA -> TTA	5283
Fg 7 - OTS	1 -	polyprotein 1ab CDS	T -> A	TCT -> TCA	5296
Fg 7 - OTS	2 -	polyprotein 1ab CDS	CTT -> TTA	CTT -> TTA	5299
Fg 7 - OTS	2 -	polyprotein 1ab CDS	CTT -> TTA	CTT -> TTA	5331
Fg 7 - OTS	1 -	polyprotein 1ab CDS	T -> A	TCT -> TCA	5368
Fg 7 - OTS	2 -	polyprotein 1ab CDS	CTT -> TTA	CTT -> TTA	5387
Fg 7 - OTS	3 -	polyprotein 1ab CDS	AGC -> TCG	AGC -> TCG	5393
Fg 7 - OTS	3 -	polyprotein 1ab CDS	AGC -> TCG	AGC -> TCG	5424
Fg 7 - OTS	2 -	polyprotein 1ab CDS	CTC -> TTG	CTC -> TTG	5454
Fg 7 - OTS	2 -	polyprotein 1ab CDS	CTT -> TTA	CTT -> TTA	5456
Fg 7 - OTS	2 -	polyprotein 1ab CDS	CTC -> TTG	CTC -> TTG	5462
Fg 7 - OTS	1 -	polyprotein 1ab CDS	C -> T	CTG -> TTG	5471
Fg 7 - OTS	1 -	polyprotein 1ab CDS	T -> A	TCT -> TCA	5472
Fg 7 - OTS	1 -	polyprotein 1ab CDS	C -> T	CTG -> TTG	5482
Fg 7 - OTS	1 -	polyprotein 1ab CDS	T -> A	TCT -> TCA	5483
Fg 7 - OTS	2 -	polyprotein 1ab CDS	CTT -> TTA	CTT -> TTA	5489
Fg 7 - OTS	2 -	polyprotein 1ab CDS	CTT -> TTA	CTT -> TTA	5500
Fg 7 - OTS	3 -	polyprotein 1ab CDS	AGT -> TCA	AGT -> TCA	5515
Fg 7 - OTS	1 -	polyprotein 1ab CDS	C -> T	CTG -> TTG	5551
Fg 7 - OTS	3 -	polyprotein 1ab CDS	AGT -> TCA	AGT -> TCA	5560
Fg 7 - OTS	1 -	polyprotein 1ab CDS	C -> T	CTA -> TTA	5564
Fg 7 - OTS	2 -	polyprotein 1ab CDS	CTC -> TTG	CTC -> TTG	5580
Fg 7 - OTS	1 -	polyprotein 1ab CDS	T -> A	TCT -> TCA	5587
Fg 7 - OTS	3 -	polyprotein 1ab CDS	AGC -> TCG	AGC -> TCG	5588
Fg 7 - OTS	1 -	polyprotein 1ab CDS	T -> A	TCT -> TCA	5602
Fg 7 - OTS	2 -	polyprotein 1ab CDS	CTC -> TTA	CTC -> TTA	5604
Fg 7 - OTS	3 -	polyprotein 1ab CDS	AGT -> TCA	AGT -> TCA	5613
Fg 7 - OTS	1 -	polyprotein 1ab CDS	C -> T	CTA -> TTA	5619
Fg 7 - OTS	2 -	polyprotein 1ab CDS	CTC -> TTG	CTC -> TTG	5621
Fg 7 - OTS	1 -	polyprotein 1ab CDS	T -> A	TCT -> TCA	5625
Fg 7 - OTS	1 -	polyprotein 1ab CDS	T -> A	TCT -> TCA	5634
Fg 7 - OTS	1 -	polyprotein 1ab CDS	C -> T	CTA -> TTA	5641
Fg 7 - OTS	3 -	polyprotein 1ab CDS	AGT -> TCA	AGT -> TCA	5655
Fg 7 - OTS	3 -	polyprotein 1ab CDS	AGT -> TCA	AGT -> TCA	5709
Total Fg 7 - OTS	148			Total codon changes Fg 7 = 80	

Supplementary Table 3 (continued): Modifications introduced into the SARS-CoV-2 genome.

Fg 8 - OTS	2-	polyprotein 1ab CDS	CTT -> TTA	CTT -> TTA	5824
Fg 8 - OTS	1-	polyprotein 1ab CDS	C -> T	CTA -> TTA	5852
Fg 8 - OTS	1-	polyprotein 1ab CDS	T -> A	TCT -> TCA	5879
Fg 8 - OTS	2-	polyprotein 1ab CDS	CTT -> TTA	CTT -> TTA	5897
Fg 8 - OTS	1-	polyprotein 1ab CDS	T -> A	TCT -> TCA	5901
Fg 8 - OTS	2-	polyprotein 1ab CDS	CTT -> TTA	CTT -> TTA	5905
Fg 8 - OTS	3-	polyprotein 1ab CDS	AGT -> TCA	AGT -> TCA	5913
Fg 8 - OTS	2-	polyprotein 1ab CDS	CTT -> TTA	CTT -> TTA	5914
Fg 8 - OTS	2-	polyprotein 1ab CDS	CTC -> TTG	CTC -> TTG	5932
Fg 8 - OTS	3-	polyprotein 1ab CDS	AGT -> TCA	AGT -> TCA	5937
Fg 8 - OTS	2-	polyprotein 1ab CDS	CTC -> TTG	CTC -> TTG	5952
Fg 8 - OTS	3-	polyprotein 1ab CDS	AGT -> TCA	AGT -> TCA	5953
Fg 8 - OTS	2-	polyprotein 1ab CDS	CTC -> TTG	CTC -> TTG	5979
Fg 8 - OTS	1-	polyprotein 1ab CDS	T -> A	TCT -> TCA	5981
Fg 8 - OTS	1-	polyprotein 1ab CDS	C -> T	CTA -> TTA	6034
Fg 8 - OTS	1-	polyprotein 1ab CDS	T -> A	TCT -> TCA	6037
Fg 8 - OTS	1-	polyprotein 1ab CDS	C -> T	CTA -> TTA	6042
Fg 8 - OTS	1-	polyprotein 1ab CDS	C -> G	TCC -> TCG	6059
Fg 8 - OTS	3-	polyprotein 1ab CDS	AGT -> TCA	AGT -> TCA	6062
Fg 8 - OTS	2-	polyprotein 1ab CDS	CTC -> TTG	CTC -> TTG	6074
Fg 8 - OTS	2-	polyprotein 1ab CDS	CTT -> TTA	CTT -> TTA	6077
Fg 8 - OTS	2-	polyprotein 1ab CDS	CTT -> TTA	CTT -> TTA	6082
Fg 8 - OTS	3-	polyprotein 1ab CDS	AGT -> TCA	AGT -> TCA	6096
Fg 8 - OTS	2-	polyprotein 1ab CDS	CTT -> TTA	CTT -> TTA	6099
Fg 8 - OTS	2-	polyprotein 1ab CDS	CTC -> TTG	CTC -> TTG	6102
Fg 8 - OTS	1-	polyprotein 1ab CDS	T -> A	TCT -> TCA	6103
Fg 8 - OTS	1-	polyprotein 1ab CDS	T -> A	TCT -> TCA	6119
Fg 8 - OTS	1-	polyprotein 1ab CDS	C -> T	CTA -> TTA	6134
Fg 8 - OTS	1-	polyprotein 1ab CDS	C -> G	TCC -> TCG	6143
Fg 8 - OTS	1-	polyprotein 1ab CDS	T -> A	TCT -> TCA	6155
Fg 8 - OTS	1-	polyprotein 1ab CDS	C -> T	CTA -> TTA	6178
Fg 8 - OTS	3-	polyprotein 1ab CDS	AGC -> TCG	AGC -> TCG	6180
Fg 8 - OTS	1-	polyprotein 1ab CDS	C -> T	CTG -> TTG	6184
Fg 8 - OTS	3-	polyprotein 1ab CDS	AGT -> TCA	AGT -> TCA	6196
Fg 8 - OTS	1-	polyprotein 1ab CDS	C -> T	CTA -> TTA	6205
Fg 8 - OTS	2-	polyprotein 1ab CDS	CTT -> TTA	CTT -> TTA	6254
Fg 8 - OTS	3-	polyprotein 1ab CDS	AGT -> TCA	AGT -> TCA	6282
Fg 8 - OTS	1-	polyprotein 1ab CDS	T -> A	TCT -> TCA	6294
Fg 8 - OTS	1-	polyprotein 1ab CDS	T -> A	TCT -> TCA	6299
Fg 8 - OTS	1-	polyprotein 1ab CDS	C -> T	CTA -> TTA	6308
Fg 8 - OTS	1-	polyprotein 1ab CDS	C -> G	TCC -> TCG	6321
Fg 8 - OTS	1-	polyprotein 1ab CDS	C -> T	CTA -> TTA	6331
Fg 8 - OTS	1-	polyprotein 1ab CDS	T -> A	TCT -> TCA	6332
Fg 8 - OTS	2-	polyprotein 1ab CDS	CTT -> TTA	CTT -> TTA	6334
Fg 8 - OTS	3-	polyprotein 1ab CDS	AGT -> TCA	AGT -> TCA	6343
Fg 8 - OTS	3-	polyprotein 1ab CDS	AGT -> TCA	AGT -> TCA	6359
Fg 8 - OTS	1-	polyprotein 1ab CDS	T -> A	TCT -> TCA	6373
Fg 8 - OTS	3-	polyprotein 1ab CDS	AGT -> TCA	AGT -> TCA	6375
Fg 8 - OTS	1-	polyprotein 1ab CDS	T -> A	TCT -> TCA	6379
Fg 8 - OTS	1-	polyprotein 1ab CDS	C -> T	CTA -> TTA	6393
Fg 8 - OTS	1-	polyprotein 1ab CDS	T -> A	TCT -> TCA	6395
Fg 8 - OTS	2-	polyprotein 1ab CDS	CTC -> TTG	CTC -> TTG	6420
Fg 8 - OTS	3-	polyprotein 1ab CDS	AGC -> TCG	AGC -> TCG	6432
Fg 8 - OTS	2-	polyprotein 1ab CDS	CTC -> TTG	CTC -> TTG	6444
Fg 8 - OTS	2-	polyprotein 1ab CDS	CTT -> TTA	CTT -> TTA	6451
Fg 8 - OTS	3-	polyprotein 1ab CDS	AGT -> TCA	AGT -> TCA	6453
Fg 8 - OTS	1-	polyprotein 1ab CDS	T -> A	TCT -> TCA	6477
Fg 8 - OTS	2-	polyprotein 1ab CDS	CTT -> TTA	CTT -> TTA	6509
Fg 8 - OTS	2-	polyprotein 1ab CDS	CTC -> TTG	CTC -> TTG	6524
Fg 8 - OTS	1-	polyprotein 1ab CDS	T -> A	TCT -> TCA	6549
Fg 8 - OTS	1-	polyprotein 1ab CDS	T -> A	TCT -> TCA	6555
Fg 8 - OTS	2-	polyprotein 1ab CDS	CTC -> TTG	CTC -> TTG	6571
Fg 8 - OTS	2-	polyprotein 1ab CDS	CTT -> TTA	CTT -> TTA	6594
Fg 8 - OTS	3-	polyprotein 1ab CDS	AGT -> TCA	AGT -> TCA	6599
Fg 8 - OTS	1-	polyprotein 1ab CDS	T -> A	TCT -> TCA	6606
Fg 8 – OTS	3-	polyprotein 1ab CDS	AGT -> TCA	AGT -> TCA	6613
Fg 8 – OTS	2-	polyprotein 1ab CDS	CTT -> TTA	CTT -> TTA	6614
Total Fg - 8 OTS	119			Total codon changes Fg 8 = 67	

Supplementary Table 4: Primers for gene expression analysis.

SARS-CoV-2 target gene		LHS (5'-3')	RHS (5'phosphorylated-3')
ORF1ab	1	TCC TTG GCA CCC GAG AAT TCC AAG TAC CGG CAG CAC AAG ACA TCT GT	CGT AGT GCA ACA GGA CTA AGC TCA TAB AAA AAA AAA AAA AAA AAA AAA AAA AAA AAA
	2	TCC TTG GCA CCC GAG AAT TCC AAT CGA AGC CAA TCC ATG CAC GTA CA	TGT CTT ATA GCT TCT TCG CGG GTG AAB AAA AAA AAA AAA AAA AAA AAA AAA AAA AAA
	4	TCC TTG GCA CCC GAG AAT TCC AGA CTT TAG ATC GGC GCC GTA ACT AT	GGC CAC CAG CTC CTT TAT TAC CGT TAB AAA AAA AAA AAA AAA AAA AAA AAA AAA AAA
	5	TCC TTG GCA CCC GAG AAT TCC ACT GCA GCA ATC AAT GGG CAA GCT TT	GTC ATT AGT ATA ACT ACC ACC ACG CAB AAA AAA AAA AAA AAA AAA AAA AAA AAA AAA
ORF3a	1	TCC TTG GCA CCC GAG AAT TCC AGG ATT AAC AAC TCC GGA TGA ACC GT	CGA TTG TGT GAA TTT GGA CAT GTT CAB AAA AAA AAA AAA AAA AAA AAA AAA AAA AAA
	2	TCC TTG GCA CCC GAG AAT TCC ACA GTA TAA CCA CCA ATC TGG TAG TC	ATG TTC AGA AAT AGG ACT TGT TGT GAB AAA AAA AAA AAA AAA AAA AAA AAA AAA AAA
	3	TCC TTG GCA CCC GAG AAT TCC AAG TCT GAA GTG AAG TAA CTG TGT AA	TAC AAC ACA GTC TTT TAC TCC AGA TAB AAA AAA AAA AAA AAA AAA AAA AAA AAA AAA
ORF10		TCC TTG GCA CCC GAG AAT TCC ATG CAC AAG AGT AGA CTA TAT ATC GT	AAA CGG AAA AGC GAA AAC GTT TAT AAB AAA AAA AAA AAA AAA AAA AAA AAA AAA AAA AAA
Envelope (E)		TCC TTG GCA CCC GAG AAT TCC ACA ATA TTG CAG CAG TAC GCA CAC AA	TCG AAG CGC AGT AAG GAT GGC TAG TAB AAA AAA AAA AAA AAA AAA AAA AAA AAA AAA

Supplementary Table 4 (continued): Primers for gene expression analysis

SARS-CoV-2 target gene		LHS (5'-3')	RHS (5'phosphorylated-3')
ORF1ab	1	TCC TTG GCA CCC GAG AAT TCC AAG TAC CGG CAG CAC AAG ACA TCT GT	CGT AGT GCA ACA GGA CTA AGC TCA TAB AAA AAA AAA AAA AAA AAA AAA AAA AAA AAA
Membrane (M)	1	TCC TTG GCA CCC GAG AAT TCC AAG CGT CCT AGA TGG TGT CCA GCA AT	ACG AAG ATG TCC ACG AAG GAT CAC AAB AAA AAA AAA AAA AAA AAA AAA AAA AAA AAA
	2	TCC TTG GCA CCC GAG AAT TCC AAT TTG TAA TAA GAA AGC GTT CGT GA	TGT AGC AAC AGT GAT TTC TTT AGG CAB AAA AAA AAA AAA AAA AAA AAA AAA AAA AAA
	3	TCC TTG GCA CCC GAG AAT TCC AAT AGC AAT TCC ACC GGT GAT CCA AT	TTA TTC TGT AAA CAG CAG CAA GCA CAB AAA AAA AAA AAA AAA AAA AAA AAA AAA AAA
Nucleocapsid (N)	1	TCC TTG GCA CCC GAG AAT TCC AGG GAA TTT AAG GTC TTC CTT GCC AT	GTT GAG TGA GAG CGG TGA ACC AAG AAB AAA AAA AAA AAA AAA AAA AAA AAA AAA AAA
	2	TCC TTG GCA CCC GAG AAT TCC AAA TTT CCT TGG GTT TGT TCT GGA CC	ACG TCT GCC GAA AGC TTG TGT TAC AAB AAA AAA AAA AAA AAA AAA AAA AAA AAA AAA
	3	TCC TTG GCA CCC GAG AAT TCC AAC CAC CAC GAA TTC GTC TGG TAG CT	CTT CGG TAG TAG CCA ATT TGG TCA TAB AAA AAA AAA AAA AAA AAA AAA AAA AAA AAA
Spike (S)	1	TCC TTG GCA CCC GAG AAT TCC AAG GAT CCA CAA GAA CAA CAG CCC TT	GAG ACA ACT ACA GCA ACT GGT CAT ABA AAA AAA AAA AAA AAA AAA AAA AAA AAA AA
	2	TCC TTG GCA CCC GAG AAT TCC AAG TAC TAC TAC TCT GTA TGG TTG GT	AAC CAA CAC CAT TAG TGG GTT GGA AAB AAA AAA AAA AAA AAA AAA AAA AAA AAA AAA
	3	TCC TTG GCA CCC GAG AAT TCC ACC TAG TGA TGT TAA TAC CTA TTG GC	AAA TCT ACC AAT GGT TCT AAA GCC GAB AAA AAA AAA AAA AAA AAA AAA AAA AAA AAA

Supplementary Table 5: Sequencing results of OTS viruses after in vitro and in vivo passaging.

In vitro

Mutations detected after ten or fifteen serial passages of OTS viruses and SARS-CoV-2 WT in Vero E6 cells.

Construct	Locus	Nucleotide position	Nucleotide exchange	Amino acid exchange	Frequency
OTS4-5 p.10 in Vero E6	ORF1b	19720	A → G	K2085E	90%
	S	21789	C → T	T76I	18%
	S	23525	C → T	H655Y	99%
	S	23585-23599	Deletion	del675-679	93%
	M	26895	C→ T	H125Y	43%
OTS7-8 p.10 in Vero E6	ORF1a	4979	A→ G	T1572A	19%
	ORF1b	14599	C→ T	synonymous	13%
	ORF1b	19741	G→ A	E2092K	98%
	S	22296	A→ G	H245R	87%
	S	23597-23617	Deletion	del679-685	95%
OTS-228 p.15	S	21765-21785	Deletion	del68-73	100%
	ORF10	29587	G → A	synonymous	13%
OTS-228 p.15 rep.1 in VeroE6	ORF1ab	7563	C → T	S → L	15%
	ORF1ab	7729	T → A	synonymous	77%
	ORF1ab	11460	C → T	S → L	61%
	S	22320	A → T	D → V	85%
OTS-228 p.15 rep.2 in VeroE6	ORF1ab	553	G → A	synonymous	13%
	ORF1ab	1684	C → T	synonymous	43%
	ORF1ab	2909	A → G	T→ A	21%
	ORF1ab	7334	C → T	H→ Y	49%
	ORF1ab	7729	A → C	synonymous	88%
	ORF1ab	8660	C → T	H→ Y	33%
	S	22320	A → T	D→ V	92%
OTS-228 p.15 rep.3 in VeroE6	ORF1ab	3003	A → G	E→ G	46%
	ORF1ab	7729	A → C	synonymous	31%
	ORF1ab	7932	C → T	S → L	20%
	ORF1ab	8872	G → T	L→ F	61%
	ORF1ab	16538	A → C	I→ L	18%
	S	22320	A → T	D→ V	88%
	S	26147	T → A	M→ K	49%
	3a	26532	G → A	E→ K	10%
SARS-CoV-2 WT p.15 in Vero E6	ORF1b	16609	A → G	T1048A	54%
	ORF1b	17432	T → G	I1322S	100%
	S	23597-23626	Deletion	del679-688	100%

Supplementary Table 5 (continued): Sequencing results of OTS viruses after in vitro and in vivo passaging.

Mutations detected after ten serial passages of OTS-206 in TMPRSS2-expressing Vero E6 cells in 3 replicates. Only mutations with a frequency less than 10% are included.

Replicate	Locus	Nucleotide position	Nucleotide change	Amino acid change	Frequency
OTS-206_1 p.10	S	22206	A → G	D → G	68.5%
	3a	25893	T → C	N/A	78.5%
OTS-206_2 p.10	ORF1a	2840	G → A	A → T	61.6%
	ORF1a	7334	C → T	H → Y	88.6%
	S	21752	T → A	W → R	95%
OTS-206_3 p.10	ORF1a	895	A → G	N/A	12%
	ORF1a	4456	C → T	N/A	10.3%
	ORF1a	6251	A → C	N → H	10%
	ORF1a	7678	A → C	R → S	13.8%
	ORF1b	14352	C → T	T → I	22.2%
	ORF1b	15278	A → G	T → A	10.7%
	S	22206	A → G	D → G	50.5%
	S	23525	C → T	H → Y	13.5%
	3a	25893	T → C	N/A	27.8%

In vivo

Mutations detected after animal passage of OTS4-5 and OTS7-8 in Syrian hamsters. For "OTS4-5 animal4-d21", no full-length genomic sequence could be obtained; the final sequence contains N-stretches (1.4%) also localized in fragment 4 (5.5% of the 2992 nucleotides are Ns) and fragment 5 (3.4% of the 3249 nucleotides are Ns).

Construct	Locus	Nucleotide position	Nucleotide exchange	Amino acid exchange	Frequency
OTS4-5 animal4-d21	ORF1a	9442	C → T	synonymous	100%
	M	26895	C → T	H125Y	66%
	ORF6	27243	A → G	I14M	88%
	S	23585-23599	Deletion	del675-679	26%
OTS7-8 animal18-d21	ORF1b	18394	G → A	A1643T	28%

Supplementary Table 5 (continued): Sequencing results of OTS viruses after in vitro and in vivo passaging.

In vivo

Mutations detected after in vivo replication passage of OTS-228 in nasal conchae tissue of Syrian hamsters (inoculated animals). For “animal3”, “animal 7” and “animal 10”, no full-length genomic sequence could be obtained; the final sequences contain N-stretches (“animal3” 10.5%, “animal 7” 0.2% and “animal 10” 0.6%), n.a., not analyzed due to low coverage

Construct	Locus	Nucleotide position	Nucleotide exchange	Amino acid exchange	Frequency
OTS-228 animal3-d21	n.a.				
OTS-228 animal7-d21	n.a.				
OTS-228 animal10-d21	ORF1a	7063	C → A	Y2266*	100%
	S	25018	A → T	L1152F	23%
OTS-228 animal12-d21	ORF1a	1684	C → T	synonymous	29%
OTS-228 animal13-d21	ORF1a	7008	C → T	A2248V	100%

Gating strategy for flow cytometry analysis

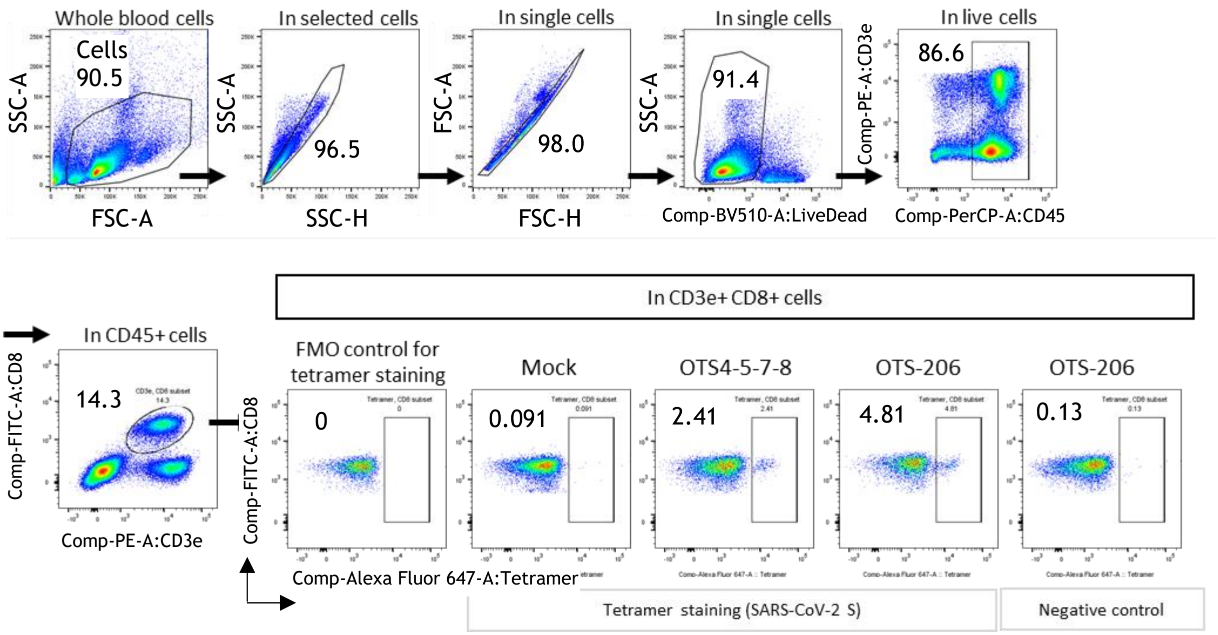

List of antibodies used for flow cytometry analysis

Live/Dead Fixable Aqua			Thermofisher
Avidin			MERCK
FcR blockung reagent, mouse	Fc Block (CD16/32)		Miltenyi biotec
**Anti-mouse antibodies**	**Dye**	**Clone**	**Company**
CD8- FITC	FITC	53-6.7	biolegend
CD45- PerCP	PerCP	30-F11	biolegend
CD3e- PE	PE	145-2C11	biolegend
H-2K(b) SARS-CoV-2 S 539-546 VNFNFNGL	Alexa Fluor 647		NIH tetramer core facility
H-2D(b) Influenza A NP 366-374 ASNENMETM	Alexa Fluor 647		NIH tetramer core facility
CD3- AF647 (compensation)	Alexa Fluor 647	145-2C11	biolegend

***Supplementary Figure 1**: **Gating strategy for the flow cytometry analysis**. Blood was collected from mock and OTS- or WT-infected mice, and red blood cells were lyzed as explained in Materials and Methods section. Antibody mixes including the following antibodies were mixed with the cells and incubated for 30 min in dark on ice: anti-mouse anti-CD8-FITC (biolegend), anti-mouse anti-CD45-PerCP (biolegend), anti-mouse anti-CD3e-PE (biolegend), either MHC-I tetramer against SARS-CoV-2 spike (H-2K(b), SARS-CoV-2 S 539-546, VNFNFNGL) (NIH), or negative control (Influenza A NP, NIH). In addition, a fluorescence minus one (FMO) control without the tetramer or negative control antibody, as well as single antibody stainings were prepared as flow cytometry control and compensation groups. Cells were washed two times with PBS, centrifuged at 350xg, 4°C for 5 min. Finally, PBS+4% paraformaldehyde (PFA) was added on the cells to fix them to take out the samples out of BSL3 for flow cytometry acquisition in FACS Canto II (BD Bioscience) using the DIVA software.*

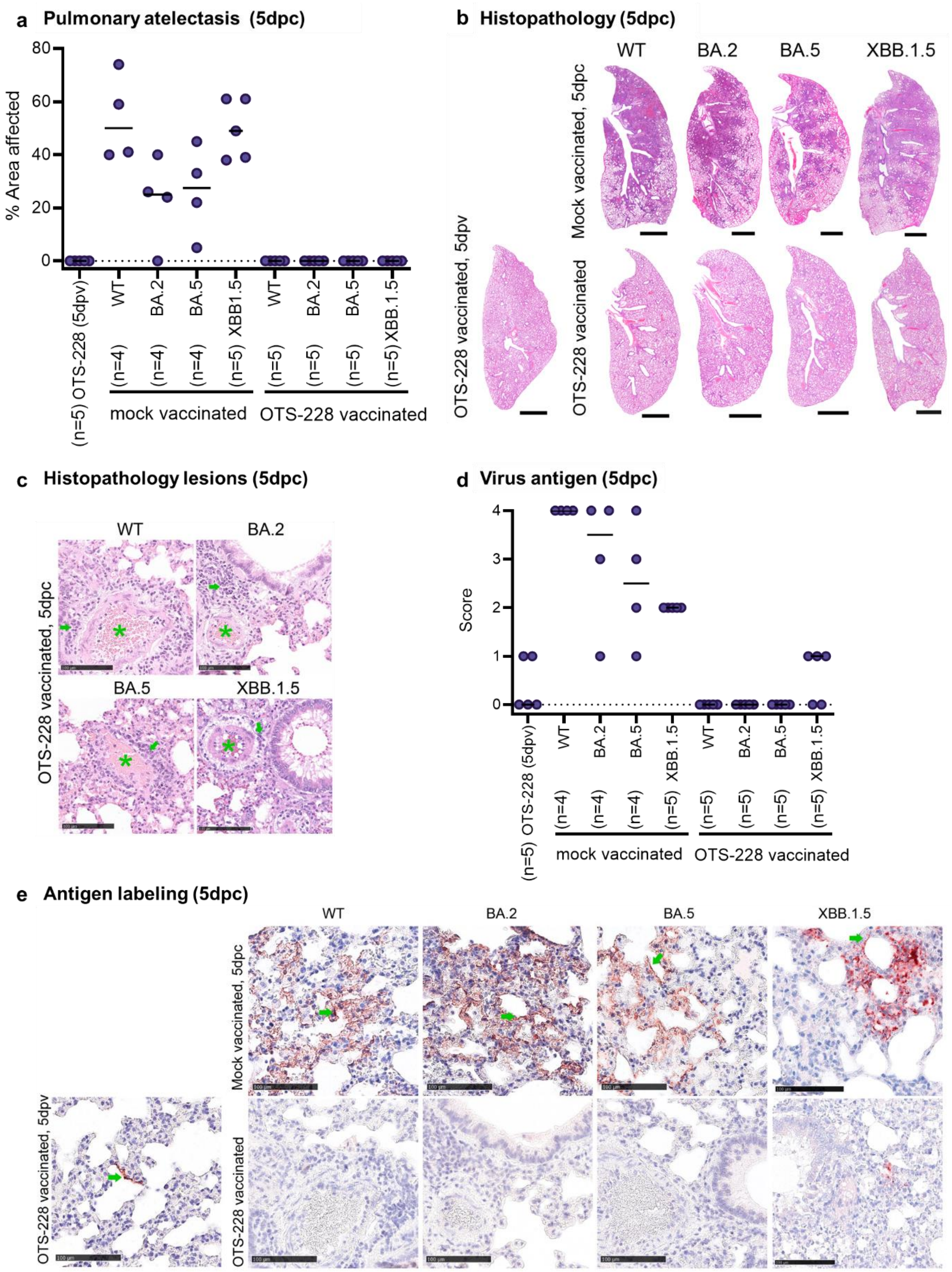

Supplementary Figure 2: Lung histopathology and virus antigen detection of OTS-228 vaccinated hamsters and 5 days after WT, Omicron BA.2, BA.5, or XBB.1.5 challenge. a, Pneumonia-induced pulmonary atelectasis given in % affected area **b**, Histopathology, lung whole slide images showing atelectasis in control animals only, hematoxylin-eosin stain. Scale bar, 2.5 mm. Analysis was done once for each hamster (mock vaccinated (n=5 hamsters each group) and OTS-228 vaccinated (n=5 hamsters each group and OTS-228 vaccinated only without challenge (n=5 hamster)) with one representative image is shown per group. **c,** Histopathology lesions. One representative image is shown per group. WT challenge infection led to perivascular (2/5) (green arrow), peribronchial (2/5), interstitial (3/5) inflammatory infiltrates, necrotizing bronchitis (1/5), activation of vascular

endothelium (1/5), pneumocyte type 2 (1/5) hyperplasia/hypertrophy, bar 100µm. BA.2 challenge led perivascular (3/5) (green arrow), peribronchial (3/5), alveolar (2/5) and interstitial (5/5) inflammatory infiltrates, necrotizing bronchitis (2/5), bronchial (2/5) hyperplasia bar 100µm. BA.5 challenge was associated with peribronchial (4/5) and perivascular (5/5) (green arrow), interstitial (4/5) inflammatory infiltrates, endothelial activation (1/5), vasculitis (1/5), alveolar edema (1/5), bronchial (2/5) and pneumocyte type 2 (1/5) hyperplasia/hypertrophy, bar 100µm. XBB.1.5 challenge yielded alveolar, perivascular and peribronchial (green arrow) immune cell infiltration (green arrow) in 5/5 hamsters, interstitial infiltrates (3/5), alveolar edema damage (5/5), activation of endothelium (2/5) bronchial (4/5) and pneumocyte type 2 (3/5) hyperplasia/hypertrophy. Scale bar, 100µm. Green asterisk indicates vascular lumen. **d,** No virus antigen was found after WT, BA.2 and BA.5 challenge, but minimal after XBB.1.5 challenge. Virus antigen score, 0 = no antigen, 1 = focal, 2 = multifocal, 3= coalescing, 4 = diffuse. **e,** Virus antigen, representative immunohistochemistry for SARS-CoV-nucleocapsid protein detection. Analysis was done once for each hamster (mock vaccinated (n=5 hamsters each group) and OTS-228 vaccinated (n=5 hamsters each group and OTS-228 vaccinated only without challenge (n=5 hamster)) with one representative image is shown per group. Minimal antigen 5 days after OTS-228 vaccination, abundant antigen in mock infected controls, no antigen (minimal subsequent XBB.1.5 challenge) in vaccinated hamsters, 5 dpc, green arrow indicates type-1 pneumocytes.Scale bar, 100 µm.

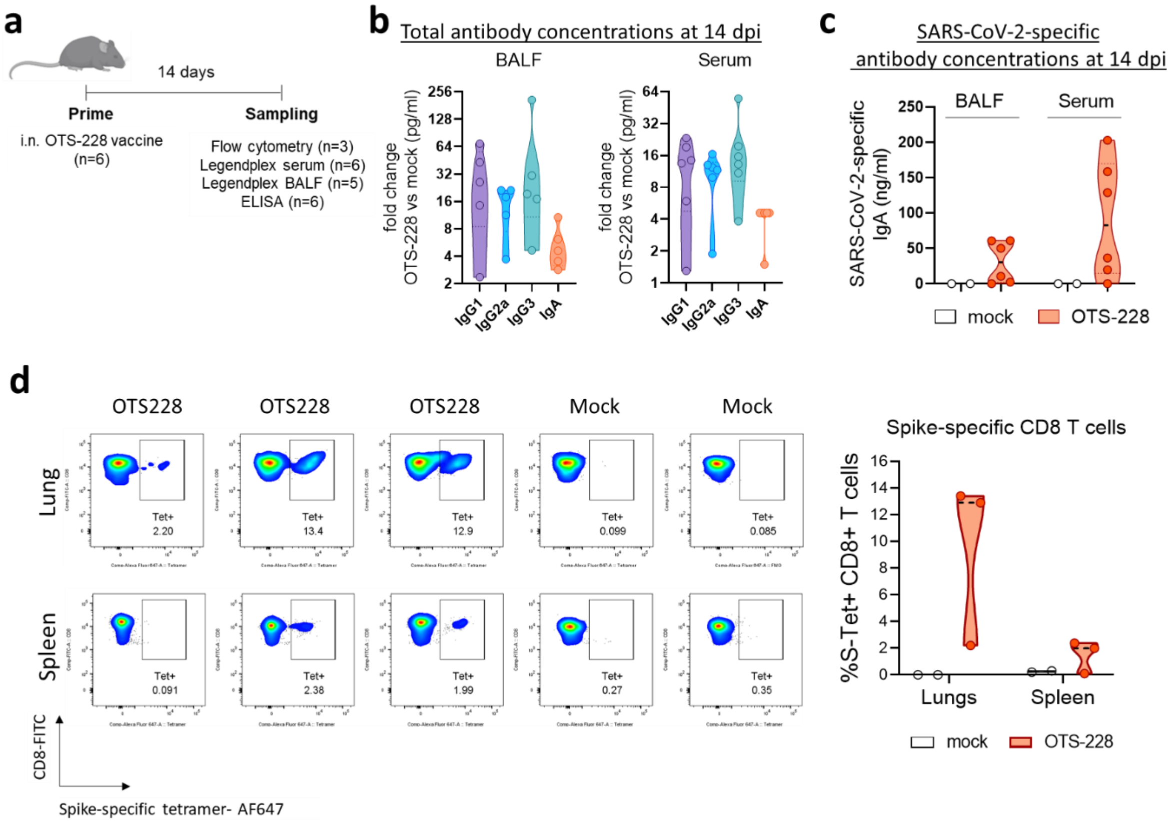

Supplementary Figure 3: Immune response to OTS-228 vaccine. a, Experimental setup of the recognition of antibodies and CD8 T cells in response to OTS-228 vaccination, 14 days post immunization (dpi). **b,** Blood and bronchoalveolar fluids (BALF) of mice (n=5) were collected upon euthanasia at 14 dpi. Sera were extracted from the blood samples (n=6). Serum and BALF samples were analyzed with LEGENDplexTM Mouse Immunoglobulin Isotyping Panel for detection of total IgG1, IgG2, IgG3 and IgA. OTS-228 data is normalized to mock data. **c,** Serum and BALF samples were analyzed for SARS-CoV-2 specific IgA antibodies with Mouse Anti-2019 nCoV (S) IgA ELISA KIT (orb756798). **d,** Homogenized lung and spleen samples from three mice vaccinated with OTS-228 and two mock vaccinated mice were stained with antibodies to detect SARS-CoV-2 spike specific CD8 T cells. The staining and flow cytometry were conducted as given in materials and methods and the gating strategy is shown in **Supplementary figure 1.**

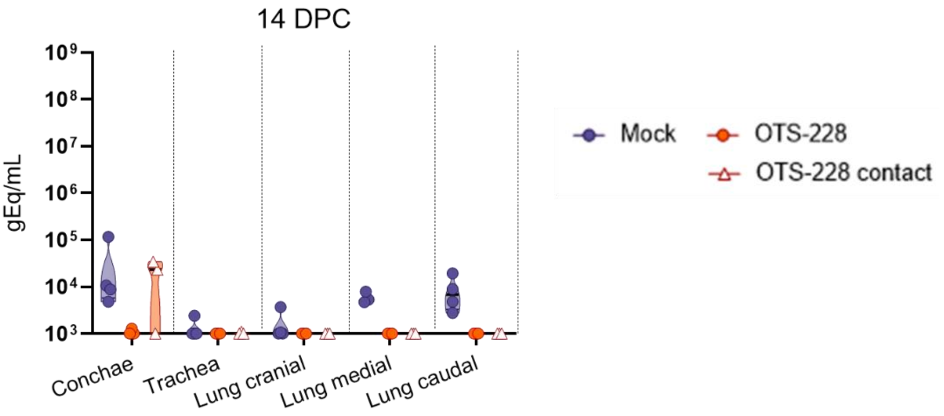

Supplementary Figure 4: Organ samples following Omicron BA.5 challenge of OTS-228 vaccinated Syrian hamsters. Virus genome copy numbers in organ samples 14 dpc. Statistical significance was assessed by two-sided, unpaired, nonparametric multiple t-test with Mann-Whitney test (compared ranks). *P<0.05, **P<0.01, ***P<0.001, ****P<0.0001. No asterisk indicates no statistical significance.

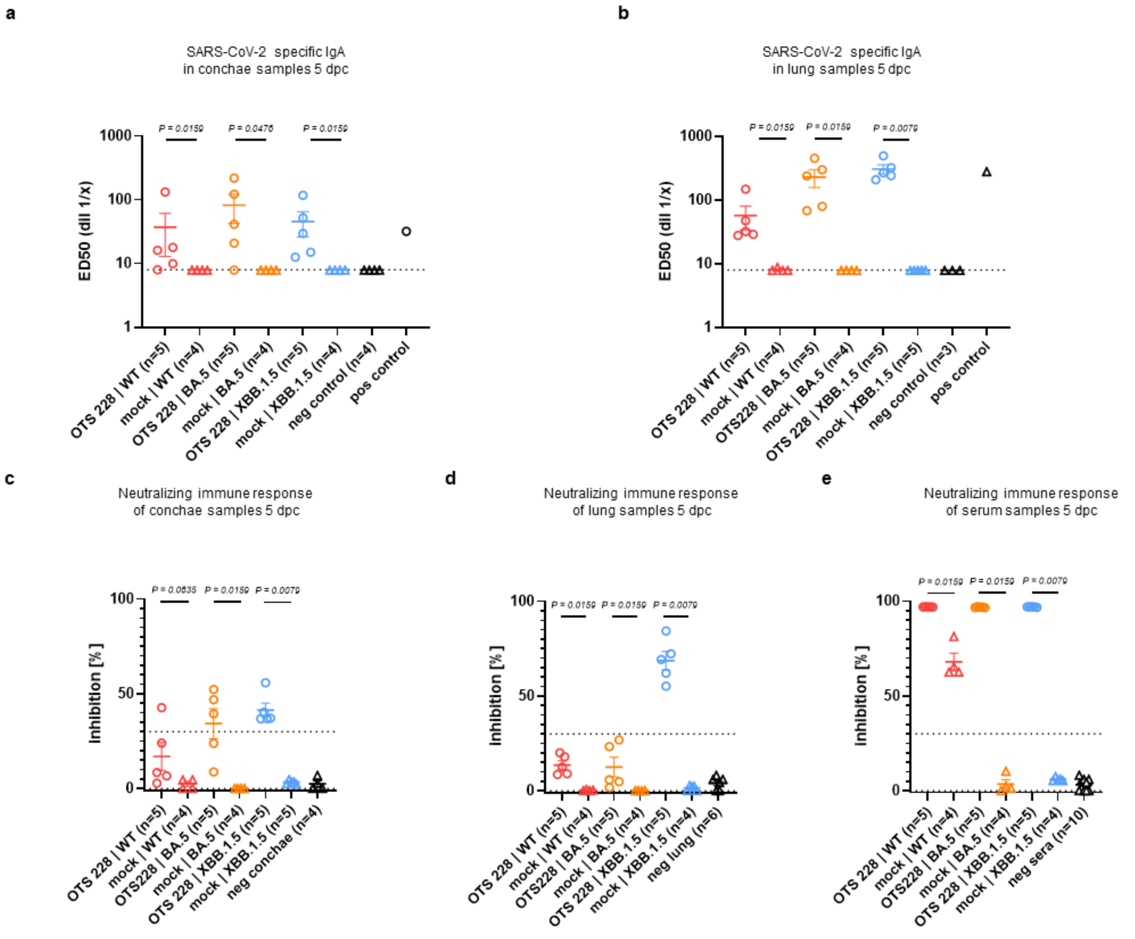

Supplementary Figure 5: Detectable mucosal immunity in OTS-228 Syrian hamsters already five days post challenge with SARS-CoV-2 WT, BA.5 or XBB1.5. SARS-CoV-2 specific IgA were detected in **a,** conchae samples and **b,** cranial lung samples. Neutralizing capacity of **c,** conchae and **d,** lung samples were confirmed and compared to neutralizing capacity of **e,** sera samples. Lines and error bars present mean values +/- SEM. Statistical significance was assessed by two-sided, unpaired, nonparametric multiple t-test with Mann-Whitney test (compared ranks).

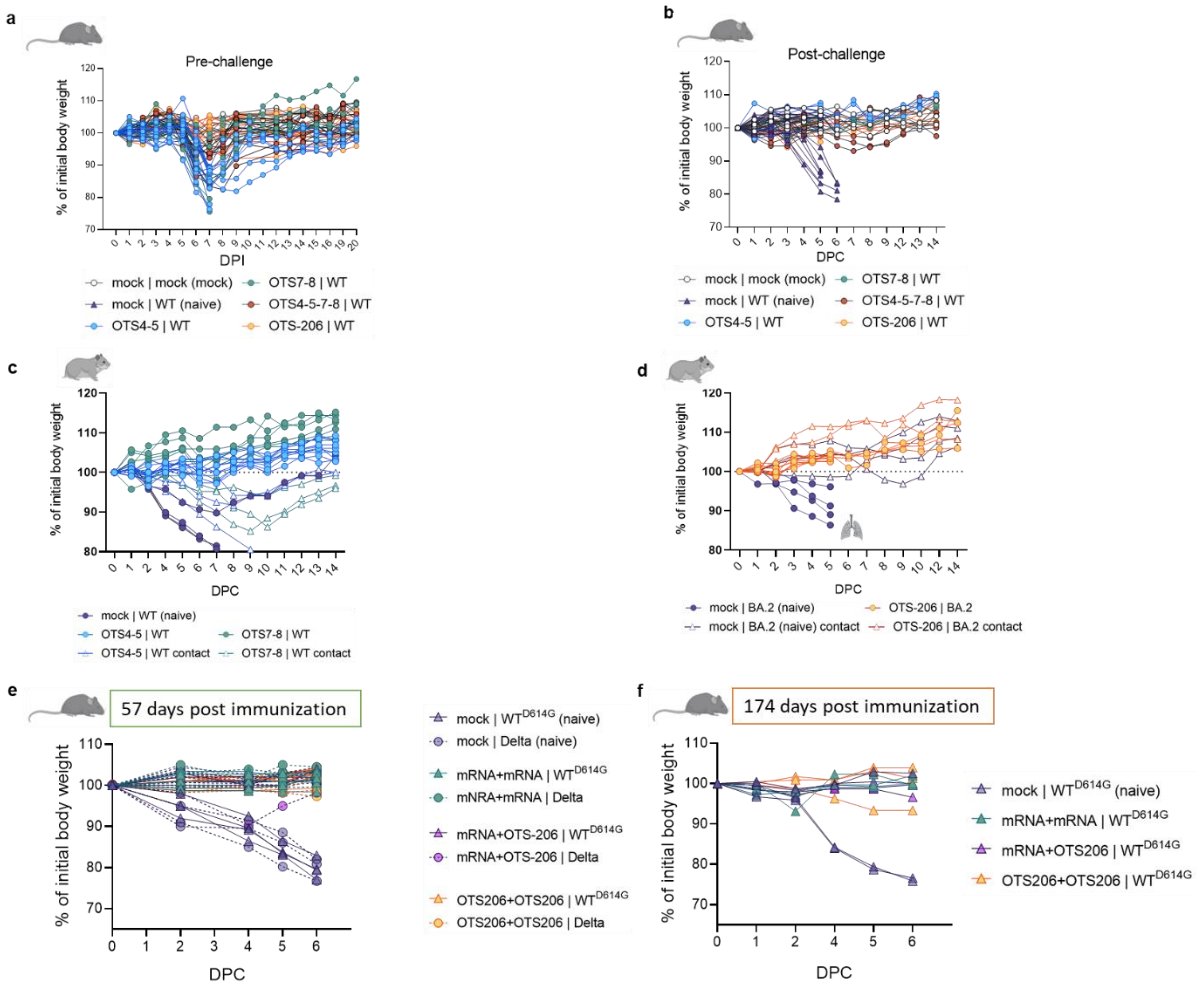

Supplementary Figure 6: Individual body weight changes in k18-hACE2 mouse and Syrian hamster infection studies. a, Body weight changes of k18-hACE2 mice post inoculation with OTS4-5, OTS7-8, OTS4-5-7-8 or OTS-206. **b,** Body weight changes post challenge of the OTS-vaccinated k18-hACE2 mice. **c,** Body weight changes of OTS4-5 and OTS7-8 vaccinated and WT SARS-CoV-2 challenged Syrian hamsters. **d,** Body weight changes of OTS-206 vaccinated and BA.2 SARS-CoV-2 challenged Syrian hamsters.

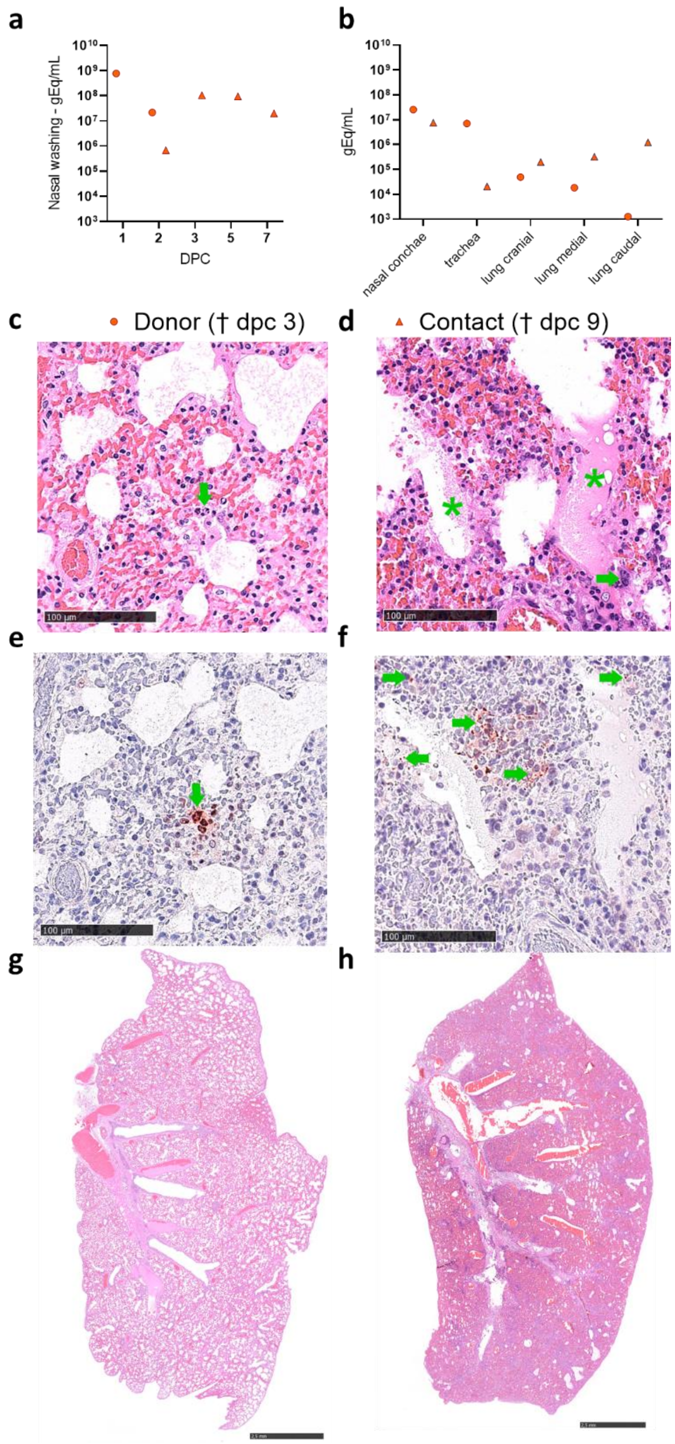

Supplementary Figure 7: Detailed examination of OTS-228 vaccinated and XBB.1.5 challenged donor animal and its contact animal succumbed at dpc 3 and 9. a, Viral genomes detected in nasal washing samples and **b,** organ samples of the succumbed donor and contact animal. **c,** Focally restricted necrosis of the alveolar epithelium (green arrow) with sloughing into the lumen observed in donor, **d,** interstitial infiltrates, hypertrophy/hyperplasia of type II pneumocytes (green arrow), diffuse alveolar edema (green asterisk), and congestion in contact animal, Scale bar, 100 µm. **e,** Virus antigen, for SARS-CoV-nucleocapsid protein detection, focally restricted (green arrow) and associated with the lesion in donor and **f,** lesion associated, multifocal (green arrows) antigen in the contact animal. Scale bar, 100 µm. **g,** Histopathology, lung whole slide images showing no atelectasis in donor and **h,** 53 % atelectasis in the contact animal. Scale bar, 2.5 mm.
