# Supplementary material for: A safe, effective and adaptable live-attenuated SARS-CoV-2 vaccine to reduce disease and transmission using one-to-stop genome modifications
Source: Nat Microbiol. 2024 Jul 12;9(8):2099–112. doi: 10.1038/s41564-024-01755-1 (PMC11306094; doi:10.1038/s41564-024-01755-1)
Supplement: Supplementary file 2 — Reporting Summary [file 41564_2024_1755_MOESM2_ESM.pdf]

nature portfolio

Corresponding author(s): Martin Beer
Volker Thiel

Last updated by author(s): 2024.04.04

Reporting Summary

Nature Portfolio wishes to improve the reproducibility of the work that we publish. This form provides structure for consistency and transparency in reporting. For further information on Nature Portfolio policies, see our Editorial Policies and the Editorial Policy Checklist.

Statistics

For all statistical analyses, confirm that the following items are present in the figure legend, table legend, main text, or Methods section.

n/a	Confirmed	
☐	☒	The exact sample size (n) for each experimental group/condition, given as a discrete number and unit of measurement
☐	☒	A statement on whether measurements were taken from distinct samples or whether the same sample was measured repeatedly
☐	☒	The statistical test(s) used AND whether they are one- or two-sided<br>*Only common tests should be described solely by name; describe more complex techniques in the Methods section.*
☐	☒	A description of all covariates tested
☐	☒	A description of any assumptions or corrections, such as tests of normality and adjustment for multiple comparisons
☐	☒	A full description of the statistical parameters including central tendency (e.g. means) or other basic estimates (e.g. regression coefficient) AND variation (e.g. standard deviation) or associated estimates of uncertainty (e.g. confidence intervals)
☐	☒	For null hypothesis testing, the test statistic (e.g. F, t, r) with confidence intervals, effect sizes, degrees of freedom and P value noted<br>*Give P values as exact values whenever suitable.*
☒	☐	For Bayesian analysis, information on the choice of priors and Markov chain Monte Carlo settings
☒	☐	For hierarchical and complex designs, identification of the appropriate level for tests and full reporting of outcomes
☒	☐	Estimates of effect sizes (e.g. Cohen's d, Pearson's r), indicating how they were calculated

Our web collection on statistics for biologists contains articles on many of the points above.

Software and code

Policy information about availability of computer code

Data collection	ELISA: Tecan i-control 2014 1.11<br>qRT-PCR: QuantStudio™ Real-Time PCR Software (v1.7.1), or 7500 Fast System SDS Software Version 1.4<br>Viral titers: manual counting, registered in Microsoft Excel 2016 (16.0.5239.1001)
Data analysis	Relative variant quantification: Bio-Rad CFX Maestro 1.1 Version 4.1.2433.1219<br>Sequence analysis: Geneious Prime ® 2019.2.3<br>Statistical analysis: GraphPad Prism version 8<br>NGS: Genome Sequencer Software Suite (version 2.6; Roche, https://roche.com), variant analysis tool integrated in Geneious Prime (2019.2.3)<br>ELISA: Microsoft Excel 2016 (16.0.5188.1000)<br>Figures: GraphPad Prism 8.4.2 (679) for Windows, Microsoft PowerPoint 2016 (16.0.4266.1001), Adobe Photoshop CC2018, Adobe Illustrator 2022<br>Digital PCR: QuantaSoft Analysis Pro software (version 1.0.596)<br>All data: Microsoft Excel 2016 (16.0518.1000)

For manuscripts utilizing custom algorithms or software that are central to the research but not yet described in published literature, software must be made available to editors and reviewers. We strongly encourage code deposition in a community repository (e.g. GitHub). See the Nature Portfolio guidelines for submitting code & software for further information.

Data

Policy information about availability of data

All manuscripts must include a data availability statement. This statement should provide the following information, where applicable:

- Accession codes, unique identifiers, or web links for publicly available datasets
- A description of any restrictions on data availability
- For clinical datasets or third party data, please ensure that the statement adheres to our policy

All data are available in the main text or the supplementary materials.

Human research participants

Policy information about studies involving human research participants and Sex and Gender in Research.

Reporting on sex and gender	NA
Population characteristics	NA
Recruitment	Lung tissue for the generation of human nasal (hNECs) nd human bronchial epithelial cells (hBECs) was obtained from patients undergoing pulmonary resection at the University Hospital of Bern, Inselspital, Switzerland, and the Cantonal Hospital of St. Gallen, Switzerland, respectively.
Ethics oversight	Written informed consent was obtained for all the patients and the study protocols were approved by the respective local Ethics Commissions (KEK-BE_2018-01801, EKSG 11/044, and EKSG 11/103).

Note that full information on the approval of the study protocol must also be provided in the manuscript.

Field-specific reporting

Please select the one below that is the best fit for your research. If you are not sure, read the appropriate sections before making your selection.

☒ Life sciences ☐ Behavioural & social sciences ☐ Ecological, evolutionary & environmental sciences

For a reference copy of the document with all sections, see nature.com/documents/nr-reporting-summary-flat.pdf

Life sciences study design

All studies must disclose on these points even when the disclosure is negative.

Sample size	Used amounts of samples were based on in-house protocols and are stated in the respective Material and Methods sections.
Data exclusions	No data were excluded from analysis.
Replication	Experiments were performed according to best practices and as described in the methods.
Randomization	PCR analysis and ELISA do not require randomization. Similarly, no randomization was required for any of in vitro and in vivo competition experiments because the viral and host response parameters were measured within each cell culture insert or each animal. Animals were randomly assigned to the respective study groups, no further criteria for assignment were defined.
Blinding	Blinding was not done for in vivo experiments. Investigators were blinded during analysis of viral plaque and qRT-PCR assays of in vitro and in vivo experiments.

Reporting for specific materials, systems and methods

We require information from authors about some types of materials, experimental systems and methods used in many studies. Here, indicate whether each material, system or method listed is relevant to your study. If you are not sure if a list item applies to your research, read the appropriate section before selecting a response.

Materials & experimental systems

n/a	Involved in the study
☐	☒ Antibodies
☐	☒ Eukaryotic cell lines
☒	☐ Palaeontology and archaeology
☐	☒ Animals and other organisms
☒	☐ Clinical data
☒	☐ Dual use research of concern

Methods

n/a	Involved in the study
☒	☐ ChIP-seq
☐	☒ Flow cytometry
☒	☐ MRI-based neuroimaging

Antibodies

Antibodies used

rabbit polyclonal anti-SARS-CoV nucleocapsid antibody (Rockland, 200-401-AS0)
biotinylated goat anti-mouse antibody (Vector Laboratories, Burlingame, CA, USA)
anti M protein of Influenza A virus antibody (ATCC clone HB-64)

Flow cytometry:
Live/Dead Fixable Aqua (Thermofisher)
Avidin (MERCK)
FcR Blocking reagent, mouse, Fc Block (CD16/32) (Miltenyi biotec)
CD8-FITC (53-6.7) (Biolegend)
CD45-Percp (30-F11) (Biolegend)
CD3e-PE (145-2C11) (Biolegend)
CD3- AF647 (compensation) (145-2C11) (Biolegend)
Alexa Fluor 647 conjugated AH-2K(b) SARS-CoV-2 S 539-546 VNFNFNGL (NIH tetramer core facility)
Alexa Fluor 647 conjugated H-2D(b) Influenza A NP 366-374 ASNENMETM

Validation

Relevant validation information can be accessed at
https://www.novusbio.com/products/sars-nucleocapsid-proteinantibody_nb100-56576#reviews-publications
https://www.biolegend.com/en-us/products/fitc-anti-mouse-cd8a-antibody-153
https://www.biolegend.com/en-us/products/percp-anti-mouse-cd45-antibody-4265
https://www.biolegend.com/en-us/products/pe-anti-mouse-cd3epsilon-antibody-25
https://www.biolegend.com/en-us/products/alexa-fluor-647-anti-mouse-cd3epsilon-antibody-2677
https://tetramer.yerkes.emory.edu/reagents/class-i-mhc/4174
https://tetramer.yerkes.emory.edu/reagents/class-i-mhc/4131

Eukaryotic cell lines

Policy information about cell lines and Sex and Gender in Research

Cell line source(s)

Vero E6 cells (FLI): Collection of Cell Lines in Veterinary Medicine CCLV RIE 0929
Vero E6 cells (IVI, IFIK): cells were kindly provided by Doreen Muth, Marcel Müller, and Christian Drosten, Charité, Berlin, Germany (ATCC CRL-1586)
Vero-TMPRSS2 cells were kindly provided by Stefan Pöhlmann, German Primate Center - Leibniz Institute for Primate Research, Göttingen, Germany)

Authentication

in-house authetication for cell lines was not performed

Mycoplasma contamination

in-house Mycoplasma exclusion is performed regularly

Commonly misidentified lines
(See ICLAC register)

NA

Animals and other research organisms

Policy information about studies involving animals; ARRIVE guidelines recommended for reporting animal research, and Sex and Gender in Research

Laboratory animals

Mesocricetus auratus, Syrian hamster, male and female, 4-12 weeks
Mus musculus, mice B6.Cg-Tg(K18-ACE2)2Prlmn/J, male and female, 7-14 weeks

Wild animals

no wild animals were used

Reporting on sex

Sex of the animals was determined by supplier and adspectorally confirmed when recieved. The pre-clinical live vaccine attenuation and efficacy studies involve in total 154 male hamsters.
From previous experiences of experimental inoculations no relevance of sex detected in this model species.

Field-collected samples

field samples were not collected

Ethics oversight	All hamster experiments were evaluated by the responsible ethics committee of the State Office of Agriculture, Food Safety, and Fishery in Mecklenburg-Western Pomerania (LALLF M-V) and gained governmental approval under registration number LVL MV TSD/7221.3-1-041/20. Mouse studies were approved by the Commission for Animal Experimentation of the Cantonal Veterinary Office of Bern and conducted in compliance with the Swiss Animal Welfare legislation and under license BE43/20.

Note that full information on the approval of the study protocol must also be provided in the manuscript.

Flow Cytometry

Plots

Confirm that:

☒ The axis labels state the marker and fluorochrome used (e.g. CD4-FITC).

☒ The axis scales are clearly visible. Include numbers along axes only for bottom left plot of group (a 'group' is an analysis of identical markers).

☒ All plots are contour plots with outliers or pseudocolor plots.

☒ A numerical value for number of cells or percentage (with statistics) is provided.

Methodology

Sample preparation	Blood was collected from mock and OTS- or WT-infected mice, and red blood cells were lyzed as explained in Materials and Methods section. Antibody mixes including the following antibodies were mixed with the cells and incubated for 30 min in dark on ice: anti-mouse anti-CD8-FITC (biolegend), anti-mouse anti-CD45-PerCP (biolegend), anti-mouse anti-CD3e-PE (biolegend), either MHC-1 tetramer against SARS-CoV-2 spike (H-2K(b), SARS-CoV-2 S 539-546, VNFNFNGL) (NIH), or negative control (Influenza A NP, NIH). In addition, a fluorescence minus one (FMO) control without the tetramer or negative control antibody, as well as single antibody stainings were prepared as flow cytometry control and compensation groups. Cells were washed two times with PBS, centrifuged at 350xg, 4°C for 5 min. Finally, PBS+4% paraformaldehyde (PFA) was added on the cells to fix them to take out the samples out of BSL3 for flow cytometry acquisition in FACS Canto II (BD Bioscience) using the DIVA software.
Instrument	FACS Canto II (BD Bioscience)
Software	DIVA software
Cell population abundance	Detected LiveCD45+CD3e+CD8+SpikeTetramer+ cells: 0-4%
Gating strategy	FSC vs SSC: gated on (mainly) lymphocytes<br>SSC-A vs SSC-H: single cells<br>FSC-A vs FSC-H: single cells<br>In single cells: CD3e-PE vs CD45-PerCp: CD45 positive cells<br>In CD45+ cells: CD3e-PE vs CD8-FITC: CD45+CD3e+CD8+ cells<br>In CD45+CD3e+CD8+ cells: CD8-FITC vs Tetramer-A647: Tetramer+ cells<br>Shown in Supplementary Fig.1

☒ Tick this box to confirm that a figure exemplifying the gating strategy is provided in the Supplementary Information.
